# Ewé: a web-based ethnobotanical database for storing and analysing data

**DOI:** 10.1093/database/baz144

**Published:** 2020-02-12

**Authors:** Estevão do Nascimento Fernandes de Souza, Julie A Hawkins

**Affiliations:** School of Biological Sciences, University of Reading, Whiteknights Rd, Reading, Berkshire RG66AS, UK

## Abstract

Ethnobotanical databases serve as repositories of traditional knowledge (TK), either at international or local scales. By documenting plant species with traditional use, and most importantly, the applications and modes of use of such species, ethnobotanical databases play a role in the conservation of TK and also provide access to information that could improve hypothesis generation and testing in ethnobotanical studies. Brazil has a rich medicinal flora and a rich cultural landscape. Nevertheless, cultural change and ecological degradation can lead to loss of TK. Here, we present an online database developed with open-source tools with a capacity to include all medicinal flora of Brazil. We present test data for the Leguminosae comprising a total of 2078 records, referred to here as use reports, including data compiled from literature and herbarium sources. Unlike existing databases, Ewé provides tools for the visualization of large datasets, facilitating hypothesis generation and meta-analyses. The Ewé database is currently available at www.ewedb.com.

## Introduction

The documentation of ethnobotanical information is essential for a better understanding of the relations between humans and plants and to progress related disciplines such as ethnopharmacology (1–3). Ethnobotanical databases can facilitate data management and information sharing with other researchers, optimizing the workflow for analysing different datasets. Ethnobotanical databases, including those of medicinally used plant species, are available and many can be accessed online. These databases may have an international focus, e.g. NAPRALERT, a database of natural products that includes ethnobotanical data (4), or a regional one, e.g. the Prelude database, which is focused on African medicinal plant use (5). Others serve a specific purpose such as the Medicinal Plant Names Services from Kew Royal Botanical Gardens (available in http://mpns.kew.org/) that curates medicinal plant names, enabling correct nomenclature of medicinal species in order to assure safety of use. In 2012, Ningthoujam *et al.* (6) presented an extensive list of ethnopharmacological databases worldwide, reviewing the diversity of approaches to storing ethnopharmacological information. Databases currently available are at the global, regional and national levels, as well as documenting used by people belonging to particular ethnolinguistic groups; some capture unpublished data, others cite published sources (Supplementary information 1).

Most of these databases were developed for particular user groups, non-academic or academic (7). The documentation of traditional knowledge (TK) is associated with the preservation of TK, recognizing that knowledge erosion is being caused by cultural change, modernization and access to western medicine (8–11). The documentation of ongoing traditional medicine practice may also highlight species for which there are concerns about safe use (10–13). Furthermore, it contributes to the protection of intellectual property in the context of bioprospecting (14), where TK is identified as the property of communities that should be the recipients of benefit-sharing (15), both by adding value to TK and identifying ownership. The Access and Benefit Sharing (ABS) mechanism was proposed in 1992 in the Convention on Biological Diversity (CBD) and implemented in the Nagoya protocol (16). Although there is a deficit in studies to track the effectiveness of ABS, a few case studies are currently available, including one in Brazil that shows a positive relationship between industry and TK holders in supplying raw material, where there is a commitment to use biodiversity with permission from the traditional communities (17).

The increasing amount of data produced by ethnobotanists could also allow researchers to address more comprehensive and comparative questions in order to better understand plant use and selection, yet data distribution and availability are barriers to this (18). More collaborative ethnobotanical research could be achieved by bridging the gap between ethnobotanists and modern bioinformatics (7). Ethnobotanical data are quite often spread across different institutions or sources, such as publications, herbarium specimen labels or unpublished theses and governmental reports that are difficult to access. Reducing the time needed for data collection would facilitate large-scale analysis (7,19,20).

Over the past 30 years, the number of publications describing ethnobotanical studies in Brazil has markedly increased (21). The amount of information generated and recorded in peer-reviewed scientific publications has the potential to inform meta-analyses, but the information should be made available in a useful format. In Brazil, Plant Database (22) provides an extensive bibliographical data compilation with more than 2000 papers; many papers focused on pharmacological and drug discovery data, however, without specific information on TK and plant species and are not publicly accessible. There is a need in Brazil for a database that provides easy access and visualization of species information.

Here, we present a web-based ethnobotanical database designed to store medicinal plant data, together with tools to conduct simple analyses and data exploration, including geographical distribution. Open-source tools have been used to build the database, recognizing that while funding for maintenance and development of databases is difficult to secure (23), a community of users is more likely to contribute if the database can be modified to respond to their changing research needs. Also, by compiling data from both literature and herbarium sources, we expect this database to be an important source of information regarding TK of medicinal plant use in order to improve meta-analyses research in ethnobotany. To exemplify the uses of this database, we also discuss two studies that used data from Ewé. The first study tests whether ethnobotanical literature and herbarium label data concerning medicinal plant uses are comparable (20). The second study uses the data from Ewé to compare traditional use and pharmacological research effort (24).

**Figure 1:**
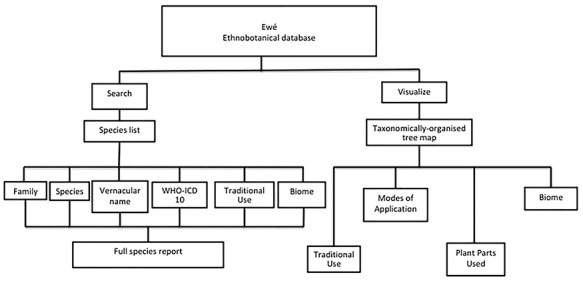
Organization of the Ewé database. The Ewé database has two functions: the search function and the visualize function. The search function queries a species list. Results can be organized according to any of the content elements (categories) or a full use report can be generated, including information on plant parts used, modes of application and the reference/source for that report. The visualize query provides visual information for sets of ethnomedicinally used species according to taxonomic rank (family, genus or species), including interactive histograms of traditional use, modes of application, plant parts used and biomes.

**Figure 2:**
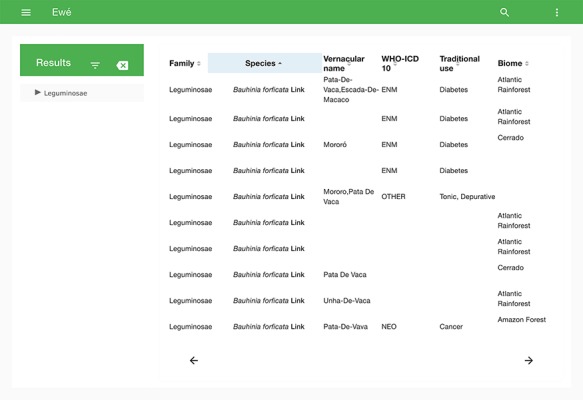
Search tool of Ewé, showing the number of records and data contents of each field of a chosen sample.

## Materials and Methods

### Database development

A web-based app was developed using MEAN (MongoDB, ExpressJS, NodeJS and AngularJS; available at http://meanjs.org/). Information for each species was organized in a spreadsheet with the following fields: family, genus, species, authority, synonym, common name, use category [etic, according to *World Health Organization International Classification of Diseases 10* (*WHO ICD*-*10*)], use category (emic, or as recorded in the field), plant part used, mode of application, country, city, biome, latitude, longitude, reference (paper author or herbarium name), collector and origin (literature or herbarium). Subsequently, it was exported in the JSON format for indexing and searching. The database functionality is summarized in Figure 1.

**Figure 3:**
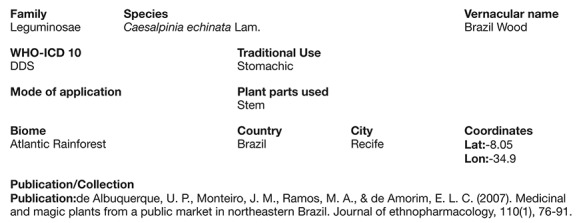
Screen shot of a full record (*Caesalpinia echinata* Lam.) illustrating its described uses, the part used, traditional use, forms of use (modes of application) and the paper reference from where it came from.

The search tool output records as a list that can be organized according to any of the content elements (categories) (Figure 2). The search field also allows the user to filter the search by any term shown in the list. Selecting one record opens the full use report for that record, including a map if the record has coordinates (Figure 3).

**Figure 4:**
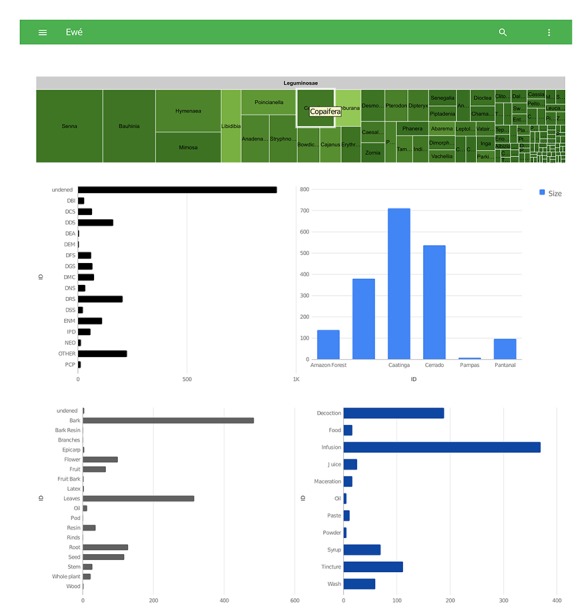
Detail of the visualization tool from Ewé showing graphs with therapeutic applications, biomes, plant parts used and modes of applications.

Data are visualized as a tree map with three interactive levels: family, genus and species. For all levels, the size of the square is proportional to the number of records. Below the tree map, there are four graphs: therapeutic applications (top left), biome distribution (top right), plant parts used (bottom left) and modes of application (bottom right). These four histograms change according to the taxonomic scale of the tree map. Also, user selection of one bar in each histogram is possible (Figure 4).

### Data collection

In order to demonstrate the functions of the database, trial data were sourced from literature and herbarium specimens for the Leguminosae of Brazil where 2078 use reports were generated for databasing. When a species was reported in one publication to be used to treat for influenza and headaches, the two therapeutic applications generated two different use reports, though the species is the same. Where necessary, geographical coordinates were assigned using Google maps and the Brazilian Geographic and Statistical Institute online data (https://cidades.ibge.gov.br/). Generic and species names followed THE PLANT LIST ( http://www.theplantlist.org ) and Missouri Botanical Garden’s Tropicos database ( http://www.tropicos.org), and they were corrected using the Plantminer R script (25).

Publications citing medicinal uses of Leguminosae species in Brazil were identified using Google Scholar and searches of the following journals: *Acta Botanica Brasilica*, *Economic Botany*, *Fitoterapia*, *Flovet*, *Journal of Ethnobiology*, *Journal of Ethnobiology and Ethnomedicine*, *Journal of Ethnopharmacology*, *Journal of Medicinal Plants Research*, *Revista Brasileira de Biociencias*, *Revista Brasileira de Farmacognosia*, *Revista Brasileira de Plantas Medicinais* and *Rodriguesia*. Herbarium data were extracted from the online list of herbarium and biological collections from Brazil, Species Link (  http://splink.cria.org.br/). The file was exported to Excel format, and a search using the keywords ‘medicinal’ and ‘uses’ (in Portuguese) was conducted in order to filter only the specimens with medicinal information. The list of publications and herbaria sourced here are presented in Supplementary information 2.

**Table 1:** Codes for *WHO ICD*-*10* used to classify traditional information

*WHO ICD*-*10*	Code
Certain conditions originating in the perinatal period	CCP
Congenital malformations, deformations and chromosomal abnormalities	CMC
Diseases of the blood and blood-forming organs and certain disorders involving the immune mechanism	DBI
Diseases of the circulatory system	DCS
Diseases of the digestive system	DDS
Diseases of the eye and adnexa	DEA
Diseases of the ear and mastoid process	DEM
Diseases of the femalegenito system	DFS
Diseases of the genitourinary system	DGS
Diseases of the musculoskeletal system and connective tissue	DMC
Diseases of the nervous system	DNS
Diseases of the respiratory system	DRS
Diseases of the skin and subcutaneous tissue	DSS
Endocrine, nutritional and metabolic diseases	ENM
Certain infectious and parasitic diseases	IPD
Mental and behavioural disorders	MBD
Neoplasms	NEO
*WHO ICD*-*10*	CODE
Symptoms, signs and abnormal clinical and laboratory findings, not elsewhere classified	OTHERS
Injury, poisoning and certain other consequences of external causes
External causes of morbidity and mortality
Factors influencing health status and contact with health services
Codes for special purposes
Pregnancy, childbirth and the puerperium	PCP

**Table 2:** Classification of different medicinal preparations into specific modes of application categories

Terms used in database	Terms used in publications or specimens notes
Decoction	Decoction
Infusion	Tea, infusion, with water, with milk
Tincture	Wine, alcoholic infusion, alcoholic extraction
Maceration	Maceration
Paste	Topic use, plaster
Juice	Drink, juice
Wash	Bath, soap, gargle, mouthwash
Food	Edible, raw, *in natura*
Oil	Oil
Syrup	Syrup, with honey
Powder	Powder/ inhale

### Data organization

In order to standardize the traditional uses, we adopted the *WHO ICD*-*10* as described in Table 1, although there are deficiencies associated with standardization (26). Biomes were assigned according to the geographical coordinates from each record, plotted on a map and intersected with the Brazilian Biomes shapefile in QGIS 2.2.0. Mode of application data was classified according to Table 2.

### Database content

The Ewé database is currently available at www.ewedb.com. In total, 2078 records were compiled to date. These were sourced from 108 publications (1331 records) and 54 herbaria (747 records). Until now, Ewé is focused on the Leguminosae from Brazil, with 322 species in 117 genera with medicinal uses. For all the databased records, 1165 (56%) indicate therapeutic applications (traditional/WHO uses), 948 (45%) indicate plant parts used and 790 (38%) indicate the modes of application (Supplementary information 3). All six Brazilian biomes are indicated in our records, with Caatinga and the Cerrado as the best represented.

Peer-reviewed scientific literature provided 87% of therapeutic applications, 95% of modes of application and 95% of parts used. Data sourced from herbarium voucher labels contributed to 86% of cited geographical localities.

## Discussion

The internet has contributed to an explosion of data sharing among scientists, including ethnobotanists, thus contributing toward a better understanding of the relation of humans and plant use (27,28). Nevertheless, much ethnobotanical data is still difficult to access, sometimes as the result of inadequate data management (27). Aside from storing a large dataset, we presented a database capable of data visualization permitting data exploration. To the best of our knowledge, Ewé is the first medicinal plant database to provide tools for data storage and visualization for Brazilian plants. Research can be facilitated using visualization tools, since hypothesis generation is supported by examining patterns and gaining insight into the data (29). Geographical areas poor in data can be identified, so future studies can be directed to areas of deficient knowledge.

In Ewé, we compile herbarium voucher label data and data from ethnobotanical publications. The compilation of these data have already permitted hypothesis testing, exemplifying the value of such data to research programmes. In the first instance, we were able to compare the data from the different sources (20). Our hypothesis was that herbaria hold significant ethnobotanical data that are complementary to the data from the published literature. We found that species reported, their therapeutic applications, modes of application and the plant part used were congruent between herbarium and literature reports. Though herbarium labels are not as rich as literature reports, we were able to use the mapping facility in Ewé to demonstrate that the spatial distribution of the use was greater than previously reported. This study, as well as validating our inclusion of herbarium data in Ewé, has highlighted an underutilized source of data for ethnobotanical studies.

In another study, we use data from Ewé to address a novel hypothesis. Our hypothesis was that ethnomedicinally important plant species and lineages have been more often characterized phytochemically or pharmacologically. With the data provided by Ewé, a phylogenetic investigation showed a phylogenetic overdispersion of medicinal uses of the Legumes in Brazil (24). In support of our hypothesis, this study revealed the relationship between pharmacological research effort and ethnobotanical use, confirming that research effort had followed traditional use. However, because the Ewé database is rich in associated data, we were able to extract the frequency of ethnobotanical reports for each species as well as therapeutic application data to gain a more nuanced understanding of the relationship. The study was possible because of the extensive data compilation for Ewé, presenting data beyond the species list.

Challenges in integrating data from different sources into a database, or even from different regional databases into a more comprehensive one, are related to data standardization and data management (6). Efforts to provide a common standard for ethnobotanical data were made by the International Working Group on Taxonomic Databases for Plant Sciences, who presented the Economic Botany Data Collection Standard (EBDCS) (30). Although not all fields proposed by EBDCS are present in Ewé, many fields such as source of information on use, use, plant part used, organism, vernacular names and modes of application are present here. Fields such as season of use, conservation status, ratings or popularity, problems and potential, though important, can be difficult to source from available material. In the future, Ewé can expand to include primary data, together with information related to season of use and popularity can be included if available.

The therapeutic application categories proposed by EBDCS are the same ones present in *WHO ICD*-*10* and have been successfully applied by ethnobotanists when compiling TK data. At the same time, it has been argued that not all plants and therapeutic applications can follow this standard and that a new classification category such as Cultural Diseases and Disorders should be included (31). The *WHO ICD*-*10* categories were also used in the Ewé database to facilitate data classification so users can consult the WHO website and search for the appropriate term. This classification also allows information from Ewé to be compared with other studies that used, at least in part, the same classification (32–35). Nevertheless, traditional medicinal use that were commonly cited such as pain, fever and inflammation could not be associated with any specific medical condition on the *WHO ICD*-*10* standard and therefore were assigned to ‘others’. While pain or fever describes a symptom that can be related to a diversity of diseases, cultural perception of such diseases might change across different communities, creating one more obstacle for study comparisons. Recognizing the deficiencies of any classification, we also provide the terms or classifications of therapeutic use from the original sources. By recording the emic (original) terminology for a disease or illness, together with the ICD mapping, it is possible to contribute to the conservation of TK even if it is related to a cultural disease not present at *WHO ICD*.

Herbarium vouchers are potential sources of ethnobotanical information to populate databases, as shown in Ewé, where these records were responsible for 86% of geographical localities. As efforts in digitization for herbarium specimens increase (36–40), standardization is needed and the Darwin Core standard is used to achieve this (41). However, core descriptors do not include any ethnobotanical fields so data describing TK present in herbarium vouchers are not captured. At the same time, herbarium vouchers have been described as important sources of ethnobotanical data (20,42–44), thus the inclusion of ethnobotanical information in the core descriptors for digitization could be beneficial.

Database sustainability is recognized as a significant problem for the biological sciences (6,45–47). Of the 80 databases that were listed by Ningthoujam *et al.* (6) in 2012, we found that 53 (66%) were no longer available, highlighting sustainability as a problem for ethnobotanical databases. Similarly, of the ethnobotanical databases cited in Supplementary information 1, during the reviewing process, 26% became inoperable. Being an open-source scheme, Ewé can be used on a private server as a research tool, and users can modify the code for their own needs. Furthermore, as an open-source scheme, the database can evolve to meet the changing requirements. Also, Ewé could function as a collaborative database, where researchers can include their own data for private use but would be encouraged to contribute to the publically available data.

The data currently held in the Ewé database were compiled from publically available sources, either publications or herbaria, the last mainly from the CRIA Species Link and Reflora programmes. Incorporating knowledge already in the public domain into a database might help defend against biopiracy (48). In the case of Ewé, the sites and dates of use can be extracted, providing evidence of the localities and time of use. There are challenges associated with the inclusion of new data as Ewé grows, and potentially primary data, prior informed consent (PIC) should be used as a way to guarantee proper identification of Traditional Knowledge holders in accordance to the Nagoya protocol on ABS.

As an online database, we expect Ewé to be a source of information and knowledge sharing between ethnobotanists in Brazil. Although at the present moment, Ewé is focused on the medicinal use of the Leguminosae, more taxa could be included, perhaps the entire medicinal flora of Brazil.

## Supplementary Material

Supplementary_information_1_baz144

Supplementary_information_2_baz144

Suppplementary_information_3_baz144
