# Supplementary material for: Ewé: a web-based ethnobotanical database for storing and analysing data
Source: Database (Oxford). 2020 Feb 12;2020:baz144. doi: 10.1093/database/baz144 (PMC7015817; doi:10.1093/database/baz144)
Supplement: Supplementary_information_1_baz144 [file supplementary_information_1_baz144.docx]

Supplementary information 1. Online Medicinal Plants Databases classified according to the source of information.

| Database Name | Classification | Description | Website | “Geographical Area” |
| --- | --- | --- | --- | --- |
| A guide to medicinal and aromatic plants | Secondary | Contains monographs including photos of worldwide aromatic species. | [https://www.hort.purdue.edu/newcrop/med-aro/](https://www.hort.purdue.edu/newcrop/med-aro/) | Worldwide |
| A Modern Herbal | Tertiary | A database based on a book with the same name, with more than 800 plants, with uses, dosages and preparations (this last two not for all plants listed). | [http://www.botanical.com/botanical/mgmh/mgmh.html](http://www.botanical.com/botanical/mgmh/mgmh.html) | Worldwide |
| BoDD- Botanical Dermatology Database | Secondary | A rich database with chemical information of many species regarding dermatological uses and treatments. | [http://www.botanical-dermatology-database.info/](http://www.botanical-dermatology-database.info/) | Worldwide |
| CMKb (Customary Medicinal Knowledgebase) | Tertiary | A database containing Australian medicinal plants information, including different common names, uses and chemical information for some species. | [http://biolinfo.org/cmkb/](http://biolinfo.org/cmkb/) | Australia |
| Dictionary of Chinese Herbs | Secondary | A database with Chinese TCM plants, including distribution, properties, uses and chemical compounds. | [http://alternativehealing.org/chinese_herb_dictionary.htm](http://alternativehealing.org/chinese_herb_dictionary.htm) | China |
| Dr.Duke’s Ethnobotany DB | Secondary | Database with many species data regarding phytochemical activities and uses. | [http://www.ars-grin.gov/duke/ethnobot.html](http://www.ars-grin.gov/duke/ethnobot.html) | Worldwide |
| edBD | Secondary/ Primary | A database with a great design, including preparation, dosages, uses, occurrence area and different methods of medicinal plant classification, available in many languages to enable access from different countries and people. Needs access login and password. | [http://www.edBD.org/](http://www.edBD.org/) | South America Andes Region, Kenia and Hawaii. |
| Encyclopaedia of India Medicinal Plants | Tertiary | Medicinal Plant information regarding what medicine system makes use of those plants, ex. Ayurveda. | [http://envis.frlht.org/bot_search.php](http://envis.frlht.org/bot_search.php) | India |
| Ethnobotany of the Peruvian Amazon | Secondary | List of medicinal species with occurrence on the Amazon Forest, and detailed data of a few listed. | [http://www.biopark.org/Plants-Amazon.html](http://www.biopark.org/Plants-Amazon.html) | Amazon Forest |
| Napralert | Secondary | Paid Database with phytochemical, pharmacological and ethnomedicinal data of many species. | [http://www.napralert.org/](http://www.napralert.org/) | Worldwide |
| Prelude | Secondary | Human and veterinary medicinal plant database, with information regarding the location, uses and vernacular names. | [http://www.africamuseum.be/collections/external/prelude](http://www.africamuseum.be/collections/external/prelude) | Africa |
| Prosea | Secondary | East Asia medicinal plant database, with information regarding uses, distribution, and vernacular names. | [http://proseanet.org/prosea/eprosea.php](http://proseanet.org/prosea/eprosea.php) | East Asia |
| Borneo's Biodiversity Information System | Primary/Secondary | A database reporting the diversity of medicinal plants utilized by the Dayak tribe. Plants part used and mode of utilization, chemical constituents are reported. | [https://www.borneodiversity.org/index/medicinal](https://www.borneodiversity.org/index/medicinal) | Borneo |
| MEDDB | Secondary | Contains information of medicial plant uses of tribal people of Madurai (Tamil Nadu), with parts used, modes of preparation and active constituents. | not available | India |
| Plants for a future | Secondary | A large database containing information of plants bioactivity, modes of application and plants parts used. | [https://pfaf.org/user/Default.aspx](https://pfaf.org/user/Default.aspx) | Worldwide |
| Raintree Tropical Plant Database | Secondary | Contains ethnobotanical, phytochemical and clinical research about plants in the Amazon Rainforest | [http://rain-tree.com/plants.htm#.W9IjZBNKiRs](http://rain-tree.com/plants.htm#.W9IjZBNKiRs) | Amazon Forest |
| AMED (Allied and Complementary Medicine Database) | Secondary | Complementary medicine database. Subscription required | [http://www.ovid.com/site/catalog/databases/12.jsp#horizontalTab1](http://www.ovid.com/site/catalog/databases/12.jsp#horizontalTab1) | Europe |
| TRAMIL | Primary/Secondary | Database with botanical description, preparation, posology, toxycity and phytochemistry | [http://www.tramil.net/](http://www.tramil.net/) | Caribbean |
| ETHMED | Secondary | Database with information on japanese traditional medicine | [https://ethmed.toyama-wakan.net/SearchEn/](https://ethmed.toyama-wakan.net/SearchEn/) | Japan/ Asia |
| TradiMed | Secondary | Contains information on applications, efficacy, preparation and safety of use of medicinal plants | [http://www.tradimed.com/index.asp](http://www.tradimed.com/index.asp) | Chinese and Korean |
| Ethnomedicinals for Research and Development | Secondary | Database with medicinal uses on plant species | [http://www.ethnomedicinals.com](http://www.ethnomedicinals.com) | Worldwide |
| GlobinMed (Global Information Hub on Integrated Medicine) | Secondary | Database with medicinal uses on plant species | [http://www.globinmed.com/](http://www.globinmed.com/) | Worldwide |
| HERBA (Historical Eesti rahvameditsiini Botaaniline Andmebaas) | Secondary | Database with traditionally used plant species from Estonia | [http://herba.folklore.ee/](http://herba.folklore.ee/) | Estonia |
| MAROWINA Database | Secondary | Database with medicinal uses on plant species of the Amazon forest | [https://tropilab.com/compmedplant.html](https://tropilab.com/compmedplant.html) | Amazon forest |
| Native American Ethnobotany Database | Secondary | Database with medicinal uses on plant species of North America | [http://naeb.brit.org/](http://naeb.brit.org/) | North America |
| Natural Medicines Comprehensive Database | Secondary | Database with medicinal uses on plant species and other bibliographic information | [http://naturaldatabase.therapeuticresearch.com/home.aspx?cs=&s=ND](http://naturaldatabase.therapeuticresearch.com/home.aspx?cs=&s=ND) | Worldwide |
| Natural Standard | Secondary | Database with medicinal uses on plant species and other bibliographic information | [https://naturalmedicines.therapeuticresearch.com/](https://naturalmedicines.therapeuticresearch.com/) | Worldwide |
