# Supplementary material for: Ewé: a web-based ethnobotanical database for storing and analysing data
Source: Database (Oxford). 2020 Feb 12;2020:baz144. doi: 10.1093/database/baz144 (PMC7015817; doi:10.1093/database/baz144)
Supplement: Supplementary_information_2_baz144 [file supplementary_information_2_baz144.docx]

Supplementary information 2. List of references and herbaria sourced for Ewé.

Agra, M. de F. et al., 2008. Survey of medicinal plants used in the region Northeast of Brazil Revisão. *Brazilian Journal of Pharmacognosy*, 18(August), pp.472–508.

Agra, M.D.F., Freitas, P.F. De & Barbosa-filho, J.M., 2007. Divulgação Synopsis of the plants known as medicinal and poisonous in Northeast of Brazil. , 17(1), pp.114–140.

Agra, M.F. et al., 2007. Medicinal and poisonous diversity of the flora of “Cariri Paraibano”, Brazil. *Journal of ethnopharmacology*, 111(2), pp.383–95. Available at: http://www.ncbi.nlm.nih.gov/pubmed/17236731 [Accessed May 23, 2014].

Aguiar, L.C.G.G. & Barros, R.F.., 2012. Plantas medicinais cultivadas em quintais de comunidades rurais no domínio do cerrado piauiense (Município de Demerval Lobão, Piauí, Brasil). *Revista Brasileira de Plantas Medicinais*, 14(3), pp.419–434.

Aita, A.M. et al., 2009. Espécies medicinais comercializadas como “quebra-pedras” em Porto Alegre, Rio Grande do Sul, Brasil. *Revista Brasileira de Farmacognosia*, 19(September 2007), pp.471–477.

Albuquerque, U.P. et al., 2007. Medicinal and magic plants from a public market in northeastern Brazil. *Journal of ethnopharmacology*, 110(1), pp.76–91. Available at: http://www.ncbi.nlm.nih.gov/pubmed/17056216 [Accessed January 29, 2014].

Albuquerque, U.P. De, 2001. The Use of Medicinal Plants by the Cultural Descendants of African People in Brazil. *Acta Farm. Bonaerense*, 20(2), pp.139–44.

Albuquerque, U.P.D. et al., 2007. Medicinal plants of the caatinga (semi-arid) vegetation of NE Brazil: a quantitative approach. *Journal of ethnopharmacology*, 114(3), pp.325–54. Available at: http://www.ncbi.nlm.nih.gov/pubmed/17900836 [Accessed June 14, 2014].

Albuquerque, U.P.D., 2006. Re-examining hypotheses concerning the use and knowledge of medicinal plants: a study in the Caatinga vegetation of NE Brazil. *Journal of ethnobiology and ethnomedicine*, 2, p.30. Available at: http://www.ncbi.nlm.nih.gov/pubmed/16872499 [Accessed July 31, 2014].

Albuquerque, U.P.D. & de Oliveira, R.F., 2007. Is the use-impact on native caatinga species in Brazil reduced by the high species richness of medicinal plants? *Journal of ethnopharmacology*, 113(1), pp.156–70. Available at: http://www.ncbi.nlm.nih.gov/pubmed/17616289 [Accessed January 28, 2014].

Alencar, N.L. et al., 2010. The Inclusion and Selection of Medicinal Plants in Traditional Pharmacopoeias — Evidence in Support of the Diversification. *Economic Botany*, 64(1), pp.68–79.

Almeida, C.D.F.C.B.R. et al., 2006. Medicinal plants popularly used in the Xingó region - a semi-arid location in Northeastern Brazil. *Journal of ethnobiology and ethnomedicine*, 2, p.15. Available at: http://www.pubmedcentral.nih.gov/articlerender.fcgi?artid=1444943&tool=pmcentrez&rendertype=abstract [Accessed July 31, 2014].

Althaus-ottmann, M.M., Junkes, M. & Cruz, R., 2011. Diversidade e uso das plantas cultivadas nos quintais do Bairro Fanny , Curitiba , PR , Brasil. , pp.39–49.

Amorozo, M.C.D.M., 2004. Pluraristic medicinal settings and medicinal plant use in rural communities, Mato Grosso, Brazil. *Journal of Ethnobiology*, 24(1), pp.139–161.

Amorozo, M.C.D.M., 2001. Uso e diversidade de plantas medicinais em Santo Antonio do Leverger, MT, Brasil. 189. *Acta Botanica Brasilica*, 16(2), pp.189–203.

Barboza da Silva, N.C. et al., 2012. Uso de plantas medicinais na comunidade quilombola da Barra II – Bahia , Brasil. *Boletín Latinoamericano y del Caribe de Plantas Medicinales y Aromáticas*, 11(5), pp.435–453.

Battisti, C. et al., 2013. Plantas medicinais utilizadas no município de Palmeira das Missões , RS , Brasil. , pp.338–348.

Begossi, A., 1993. Plant uses in a Brazilian coastal fishing community (Buzios Island). *Journal of Ethnobiology*, 13(2), pp.233–256.

Borba, A.M. & Macedo, M., 2006. Plantas medicinais usadas para a saúde bucal pela comunidade do bairro Santa Cruz , Chapada dos Guimarães , MT , Brasil 1. , 20(4), pp.771–782.

Botrel, R.T. et al., 2006. Uso da vegetação nativa pela população local no município de Ingaí, MG, Brasil. *Acta Botanica Brasilica*, 20(1), pp.143–156.

Brandão, M.G.L. et al., 2008. Brazilian medicinal plants described by 19th century European naturalists and in the Official Pharmacopoeia. *Journal of ethnopharmacology*, 120(2), pp.141–8. Available at: http://www.ncbi.nlm.nih.gov/pubmed/18762237 [Accessed January 29, 2014].

Brandão, M.G.L. et al., 2012. Useful Brazilian plants listed in the field books of the French naturalist Auguste de Saint-Hilaire (1779-1853). *Journal of ethnopharmacology*, 143(2), pp.488–500. Available at: http://www.ncbi.nlm.nih.gov/pubmed/22800679 [Accessed January 29, 2014].

Bratti, C. et al., 2013. Levantamento de Plantas Medicinais Nativas da Fazenda Azulão em Dourados-MS. *Revista Brasileira de Plantas Medicinais*, 15(4), pp.675–683.

Breitbach, U.B. et al., 2013. Amazonian Brazilian medicinal plants described by C . F . P . von Martius in the 19th century. , 147(November 1817), pp.180–189.

Brito, M.R. De & Senna-valle, L. De, 2012. Diversity of plant knowledge in a “ Caiçara ” community from the Brazilian Atlantic Forest coast 1. , 26(4), pp.735–747.

Bueno, N.R. et al., 2005. Medicinal plants used by the Kaiowá and Guarani indigenous populations in the Caarapó Reserve , Mato Grosso do Sul , Brazil. , 19(1), pp.39–44.

Campelo, C.R. & Ramalho, R. de C., 1989. Contribuição ao estudo das plantas medicinais no estado de alagoas -. *Acta Botanica Brasilica*, 2(1), pp.67–72.

Cartaxo, S.L., Souza, M.M.D.A. & de Albuquerque, U.P., 2010. Medicinal plants with bioprospecting potential used in semi-arid northeastern Brazil. *Journal of ethnopharmacology*, 131(2), pp.326–42. Available at: http://www.ncbi.nlm.nih.gov/pubmed/20621178 [Accessed February 27, 2013].

Castro, J.A. et al., 2011. Ethnobotanical study of traditional uses of medicinal plants : The flora of caatinga in the community of. , 5(10), pp.1905–1917.

Chaves, E.M.F. & Barros, R.F.M., 2012. Diversidade e uso de recursos medicinais do carrasco na APA da Serra da Ibiapaba , Piauí , Nordeste do Brasil. *Revista Brasileira de Plantas Medicinais*, 8(3), pp.476–486.

Christo, A.G., Guedes-bruni, R.R. & Silva, A.G., 2010. Local knowledge on medicinal plant gardens in a rural community near the Atlantic Rain Forest, southeastern Brazil. *Revista Brasileira de Farmacognosia*, 20(4), pp.494–501.

Coelho-Ferreira, M., 2009. Medicinal knowledge and plant utilization in an Amazonian coastal community of Marudá, Pará State (Brazil). *Journal of ethnopharmacology*, 126(1), pp.159–75. Available at: http://www.ncbi.nlm.nih.gov/pubmed/19632314 [Accessed July 21, 2014].

Conceição, G.. et al., 2011. Plantas do cerrado : comercialização , uso e indicação terapêutica fornecida pelos raizeiros e vendedores , Teresina , Piauí. *Scientia Plena*, 7(1), pp.1–6.

Conde, B.E. et al., 2014. Ethnopharmacology in the Vicinity of the Botanical Garden of the Federal University of Juiz de Fora , Brazil. , 12(March), pp.91–111.

Costa, V.P. & Mayworm, M.A.., 2011. Plantas medicinais utilizadas pela comunidade do bairro dos Tenentes - município de Extrema , MG , Brasil. *Revista Brasileira de Plantas Medicinais*, 13(3), pp.282–292.

Cunha, S.A. da & Bortolotto, I.M., 2011. Etnobotânica de Plantas Medicinais no Assentamento Monjolinho , município de Anastácio , Mato Grosso do Sul , Brasil. *Acta Botanica Brasilica*, 25(3), pp.685–698.

David, J.P.J.M. et al., 2007. Radical scavenging, antioxidant and cytotoxic activity of Brazilian Caatinga plants. *FITOTERAPIA*, 78(3), pp.215–218. Available at: http://www.ncbi.nlm.nih.gov/pubmed/17331673.

David, M. De & Pasa, M.C., 2013. O saber popular e as plantas medicinais em Varzea Grande, MT, Brasil. *Flovet*, 5, pp.32–50.

Desmarchelier, C. et al., 1999. Antioxidant and free radical scavenging activities in extracts from medicinal trees used in the “Caatinga” region in northeastern Brazil. *Journal of ethnopharmacology*, 67(1), pp.69–77. Available at: http://www.ncbi.nlm.nih.gov/pubmed/10616962.

Dickel, M.L., Rates, S.M.K. & Ritter, M.R., 2007. Plants popularly used for loosing weight purposes in Porto Alegre, South Brazil. *Journal of ethnopharmacology*, 109(1), pp.60–71. Available at: http://www.ncbi.nlm.nih.gov/pubmed/16963210 [Accessed July 11, 2014].

Dornas, W.C. et al., 2009. Efeitos antidiabéticos de plantas medicinais. *REVISTA BRASILEIRA DE FARMACOGNOSIA*, 19(2), pp.488–500.

Eichemberg, M.T. et al., 2009. Species composition and plant use in old urban homegardens in Rio Claro , Southeast of Brazil. , 23(4), pp.1057–1075.

Feijo, A.M. et al., 2012. Plantas medicinais utilizadas por idosos com diagnóstico de Diabetes mellitus no tratamento dos sintomas da doença. *Revista Brasileira de Plantas Medicinais*, 2006(1), pp.50–56.

Feijo, E.V.R.S. et al., 2013. Levantamento preliminar sobre plantas medicinais utilizadas no bairro Salobrinho no município de Ilhéus , Bahia. *Revista Brasileira de Plantas Medicinais*, 15(4), pp.595–604.

Fernandes, J.M. et al., 2014. Etnobotânica de Leguminosae entre agricultores agroecológicos na Floresta Atlântica, Araponga, Minas Gerais, Brasil. *Rodriguesia*, 65(2), pp.539–554.

Figueiredo, G.M., Leitao-Filho, H.F. & Begossi, A., 1993. Ethnobotany of Atlantic Forest Coastal Communities : Diversity of Plant Uses in Gamboa ( Itacuru ; fi Island , Brazil ). *Human Ecology*, 21(4), pp.419–430.

Fita, D.S., Costa Neto, E.M. & Schiavetti, A., 2010. “Offensive” snakes: cultural beliefs and practices related to snakebites in a Brazilian rural settlement. *Journal of ethnobiology and ethnomedicine*, 6, p.13. Available at: http://www.pubmedcentral.nih.gov/articlerender.fcgi?artid=2853519&tool=pmcentrez&rendertype=abstract.

Franco, E.A.P. & Barros, R.F.M., 2004. Uso e diversidade de plantas medicinais no Quilombo Olho D ’ água dos Pires , Esperantina , Piauí. *Revista Brasileira de Plantas Medicinais*, 8(3), pp.78–88.

Franco, M. et al., 2004. Plantas medicinais e seus usos pelos sitiantes da Reserva Rio das Pedras ,. *Acta Botanica Brasilica*, 18(2), pp.391–399.

Freitas, A.S.H. de J. et al., 2013. Estudo etnobotânico de cipós comercializados como medicinais por raizeiros de Cuiaba, Mato Grosso, Brasil. *Flovet*, 5, pp.71–81.

Freitas, A.V.L. et al., 2012. Plantas medicinais : um estudo etnobotânico nos quintais do Sítio Cruz , São Miguel , Rio Grande do Norte , Brasil. *Revista Brasileira de Biociências*, 10(1), pp.48–59.

Gandolfo, E.S. & Hanazaki, N., 2011. Etnobotânica e urbanização : conhecimento e utilização de plantas de restinga pela comunidade nativa do distrito do Campeche ( Florianópolis , SC ). , 25(1), pp.168–177.

Garcia, D., Domingues, M.V. & Rodrigues, E., 2010. Ethnopharmacological survey among migrants living in the Southeast Atlantic Forest of Diadema, São Paulo, Brazil. *Journal of ethnobiology and ethnomedicine*, 6(1), p.29. Available at: http://www.pubmedcentral.nih.gov/articlerender.fcgi?artid=2987905&tool=pmcentrez&rendertype=abstract [Accessed July 31, 2014].

Gazzaneo, L.R.S., de Lucena, R.F.P. & de Albuquerque, U.P., 2005. Knowledge and use of medicinal plants by local specialists in an region of Atlantic Forest in the state of Pernambuco (Northeastern Brazil). *Journal of ethnobiology and ethnomedicine*, 1, p.9. Available at: http://www.pubmedcentral.nih.gov/articlerender.fcgi?artid=1291389&tool=pmcentrez&rendertype=abstract [Accessed July 13, 2014].

Giraldi, M. & Hanazaki, N., 2010. Uso e conhecimento tradicional de plantas medicinais no Sertão do Ribeirão , Florianópolis , SC , Brasil. *Acta Botanica Brasilica*, 24(2), pp.395–406.

Gomes, T.B. & Bandeira, F.P.S. de, 2012. Uso e diversidade de plantas medicinais em uma comunidade quilombola no Raso da Catarina , Bahia. *Acta Botanica Brasilica*, 26(4), pp.796–809.

Guarim-Neto, G., 2006. O saber tradicional pantaneiro: as plantas medicinais e a educação ambiental. *Rev. eletrônica Mestr. Educ. Ambient.*, 17, pp.71–89.

Guarim-Neto, G. & Morais, R.G., 2003. Recursos medicinais de espécies do Cerrado de Mato Grosso: um estudo bibliográfico. *Acta Botanica Brasilica*, 17(4), pp.561–584.

Guarim-Neto, G. & Pasa, M.C., 2009. Estudo etnobotanico em uma area de cerrado no municipio de acorizal, Mato Grosso. *Flovet*, 1(1997), pp.5–32.

Hirschmann, G.S. & Arias, A.R., 1990. A survey of medicinal plants of Minas Gerais, Brazil. *Journal of ethnopharmacology*, 29, pp.159–172.

Jesus, N.Z.T. de et al., 2009. Divulgação Levantamento etnobotânico de plantas popularmente utilizadas como antiúlceras e antiinflamatórias pela comunidade de Pirizal , Nossa Senhora do Livramento-MT, Brasil. *Revista Brasileira de Farmacognosia*, 19(1), pp.130–139.

Kainer, K. a. & Duryea, M.L., 1992. Tapping women’s knowledge: Plant resource use in extractive reserves, acre, Brazil. *Economic Botany*, 46(4), pp.408–425. Available at: http://link.springer.com/10.1007/BF02866513.

Liporacci, H.S.N. & Simao, D.G., 2013. Levantamento etnobotânico de plantas medicinais nos quintais do Bairro Novo Horizonte , Ituiutaba , MG. *Revista Brasileira de Plantas Medicinais*, 15(4), pp.529–540.

Löbler, L. et al., 2014. Levantamento etnobotânico de plantas medicinais no bairro Três de Outubro da cidade de São Gabriel , RS , Brasil. , (2007), pp.81–89.

Lozano, A. et al., 2014. The apparency hypothesis applied to a local pharmacopoeia in the Brazilian northeast. *Journal of ethnobiology and ethnomedicine*, 10(1), p.2. Available at: http://www.pubmedcentral.nih.gov/articlerender.fcgi?artid=3903438&tool=pmcentrez&rendertype=abstract.

de Lucena, R.F.P. et al., 2008. Local Uses of Native Plants in an Area of Caatinga Vegetation (Pernambuco, NE Brazil). *Ethnobotany Research & Applications*, 6, pp.3–13.

Lucena, R.F.P. de, Araujo, E. de L. & de Albuquerque, U.P., 2007. Does the Local Availability of Woody Caatinga Plants ( Northeastern Brazil ) Explain Their Use Value ? *Economic Botany*, 61(4), pp.347–361.

Macedo, M., Pereira, M.L. de S. & Silva, F.H.B. da, 2011. Plantas com provavel acao antifungica utilizadas pelos moradores do bairo Cidade Verde, Cuiaba, Mato Grosso. *Flovet*, 3.

Maioli-azevedo, V. & Fonseca-Kruel, V.S. da, 2007. Plantas medicinais e ritualísticas vendidas em feiras livres no Município do Rio de Janeiro , RJ , Brasil : estudo de caso nas zonas Norte e Sul 1. *Acta Botanica Brasilica*, 21(2), pp.263–275.

da Mata, N.D.S. et al., 2012. The participation of Wajãpi women from the State of Amapá (Brazil) in the traditional use of medicinal plants--a case study. *Journal of ethnobiology and ethnomedicine*, 8, p.48. Available at: http://www.pubmedcentral.nih.gov/articlerender.fcgi?artid=3541996&tool=pmcentrez&rendertype=abstract.

Meretika, A.H.C., Peroni, N. & Hanazaki, N., 2010. Local knowledge of medicinal plants in three artisanal fishing communities (Itapoá, Southern Brazil), according to gender, age, and urbanization. *Acta Botanica Brasilica*, 24(2), pp.386–394.

Miranda, T.M. et al., 2011. Existe utilização efetiva dos recursos vegetais conhecidos em comunidades caiçaras da Ilha do Cardoso, estado de Sao Paulo, Brasil? *Rodriguesia*, 62(1), pp.153–169.

Monteles, R. & Pinheiro, C.U.B., 2007. Plantas medicinais em um quilombo maranhense : uma perspectiva etnobotânica Medicinal plants in a quilombola community in the State of Maranhão : An ethnobotanical approach. , 7, pp.38–48.

Nunes, G.P. et al., 2003. Plantas medicinais comercializadas por raizeiros no Centro de Campo Grande , Mato Grosso do Sul. *Revista Brasileira de Farmacognosia*, 12(2), pp.83–92.

Oliveira, a K.M. et al., 2011. Ethnobotany and traditional medicine of the inhabitants of the Pantanal Negro sub-region and the raizeiros of Miranda and Aquidauna, Mato Grosso do Sul, Brazil. *Brazilian journal of biology = Revista brasleira de biologia*, 71(1 Suppl 1), pp.283–9. Available at: http://www.ncbi.nlm.nih.gov/pubmed/21537601.

Oliveira, E.C.S. de & Trovao, D.M. de B.M., 2009. O uso de plantas em rituais de rezas e benzeduras: um olhar sobre esta prática no estado da Paraíba. *Revista Brasileira de Biociências*, 7(3), pp.245–251.

Oliveira, E.R. & Menini Neto, L., 2012. Levantamento etnobotânico de plantas medicinais utilizadas pelos moradores do povoado de Manejo , Lima Duarte - MG. *Revista Brasileira de Plantas Medicinais*, 14(2), pp.311–320.

Oliveira, F.C.S., Barros, R.F.M. & Moita Neto, J.M., 2010. Plantas medicinais utilizadas em comunidades rurais de Oeiras, semiárido piauiense. *Revista Brasileira de Plantas Medicinais*, 3(28), pp.282–301.

Oliveira, G.L. De et al., 2010. Plantas medicinais utilizadas na comunidade urbana de Muribeca , Nordeste do Brasil. , 24(2), pp.571–577.

Oliveira, H.B. De, Kffuri, C.W. & Casali, V.W.D., 2010. Ethnopharmacological study of medicinal plants used in Rosário da Limeira, Minas Gerais, Brazil. *Revista Brasileira de Farmacognosia*, 20(2), pp.256–260.

Pasa, M.C., Guarim-Neto, G. & Oliveira, W.A., 2011. A etnobotanica e as plantas usadas como remedio na comunidade bom jardim, MT, Brasil. *Flovet*, 3.

Pasa, M.C., Juares, J. & Guarim, G., 2005. Estudo etnobotânico na comunidade de Conceição-Açu ( alto da bacia do rio Aricá Açu , MT , Brasil ). , 19(2), pp.195–207.

Pereira, R.C., Oliveira, M.T.R. & Lemos, G.C.S., 2005. Plantas utilizadas como medicinais no município de Campos de Goytacazes - RJ. *Revista Brasileira de Farmacognosia*, 14(1), pp.37–40.

Pilla, M.A.C., Amorozo, M.C.D.M. & Furlan, A., 2006. Obtenção e uso das plantas medicinais no distrito de Martim Francisco , Município de Mogi-Mirim , SP , Brasil. *Acta Botanica Brasilica*, 20(4), pp.789–802.

Pinto, A.Z. de L. et al., 2013. Levantamento etnobotânico de plantas medicinais comercializadas no mercado do porto em cuiabá, mato grosso,. *Flovet*, 5, pp.51–70.

Pinto, E. de P.P., Amorozo, M.C.D.M. & Furlan, A., 2006. Conhecimento popular sobre plantas medicinais em comunidades rurais de mata atlantica- Itacare, BA, Brasil. *Acta Botanica Brasilica*, 20(4), pp.751–762.

Ritter, M.R., Sobierajski, G.R.Schenkel, E.. & Mentz, L.A., 2002. Plantas usadas como medicinais no município de Ipê , RS , Brasil. *Revista Brasileira de Farmacognosia*, 12(2), pp.51–62.

Rodrigues, E., 2007. Plants of restricted use indicated by three cultures in Brazil (Caboclo-river dweller, Indian and Quilombola). *Journal of ethnopharmacology*, 111(2), pp.295–302. Available at: http://www.ncbi.nlm.nih.gov/pubmed/17196776.

Roque, A.A., Rocha, R.M. & Loiola, M.I.B., 2010. Uso e diversidade de plantas medicinais da Caatinga na comunidade rural de Laginhas , município de Caicó , Rio Grande do Norte ( nordeste do Brasil ). *Revista Brasileira de Plantas Medicinais*, 12(1), pp.31–42.

Santos, M.M., Nunes, M.S.G. & Martins, R.D., 2012. Uso empírico de plantas medicinais para tratamento de diabetes. *Revista Brasileira de Plantas Medicinais*, 14(2), pp.327–334.

Santos, M.R.A., Lima, M.R. & Ferreira, M. das G., 2008. Uso de plantas medicinais pela população de Ariquemes, em Rondônia. *Horticultura Brasileira*, 26, pp.244–250.

Shanley, P. & Luz, L., 2003. The Impacts of Forest Degradation on Medicinal Plant Use and Implications for Health Care in Eastern Amazonia. *BioScience*, 53(6), p.573. Available at: http://www.jstor.org/stable/1314459.

Shanley, P. & Rosa, N. a., 2004. Cover article: Eroding Knowledge: An Ethnobotanical Inventory in Eastern Amazonia’s Logging Frontier. *Economic Botany*, 58(2), pp.135–160. Available at: http://link.springer.com/10.1663/0013-0001(2004)058[0135:EKAEII]2.0.CO;2.

Silva, A.J. da R. & Andrade, L. de H.C., 2005. Etnobotânica nordestina : estudo comparativo da relação entre comunidades e vegetação na Zona do Litoral - Mata do Estado de. *Acta Botanica Brasilica*, 19(1), pp.45–60.

Silva, C.S.P. da & Proença, C.E.B., 2008. Uso e disponibilidade de recursos medicinais no município de Ouro Verde, GO, Brasil. *Acta Botanica Brasilica*, 22(2), pp.481–492.

Silva, H.C.H. et al., 2014. Evaluating different methods used in ethnobotanical and ecological studies to record plant biodiversity. *Journal of ethnobiology and ethnomedicine*, 10(1), p.48. Available at: http://www.ncbi.nlm.nih.gov/pubmed/24916833 [Accessed July 31, 2014].

Silva, K.M. et al., 2013. Espécies bioativas em áreas úmidas do Planalto Catarinense. *Revista Brasileira de Plantas Medicinais*, 15(4), pp.483–493.

Silva, M.A.B. da et al., 2010. Levantamento etnobotânico de plantas utilizadas como anti-hiperlipidêmicas e anorexígenas pela população de Nova Xavantina-MT , Brasil. *Revista Brasileira de Farmacognosia*, 20(4), pp.549–562.

Silva, T.S. & Freire, E.M.X., 2010. Abordagem etnobotânica sobre plantas medicinais citadas por populações do entorno de uma unidade de conservação da caatinga do Rio Grande do Norte , Brasil. *Revista Brasileira de Plantas Medicinais*, 12(4), pp.427–435.

Siviero, A. et al., 2012. Plantas medicinais em quintais urbanos de Rio Branco, Acre. *Revista Brasileira de Plantas Medicinais*, 14(4), pp.598–610.

Sousa, S. & Hanazaki, N., 2012. Are Gender and Age Important in Understanding the Distribution of Local Botanical Knowledge in Fishing Communities of the Parnaíba Delta Environmental Protection Area ? , 10, pp.551–559.

Souza, C.D. De & Felfili, J.M., 2006. Uso de plantas medicinais na região de Alto Paraíso de Goiás , GO , Brasil. *Acta Botanica Brasilica*, 20(1), pp.135–142.

Souza, L.F., 2007. Recursos vegetais usados na medicina tradicional do Cerrado ( comunidade de Baús , Acorizal , MT , Brasil ). *Revista Brasileira de Plantas Medicinais*, 9(4), pp.44–54.

Ustulin, M. et al., 2009. Revisão Plantas medicinais comercializadas no Mercado Municipal de Campo Grande-MS. , 19(November 2008), pp.805–813.

Vila Verde, G.M., Paula, J.R. & Caneiro, D.M., 2003. Levantamento etnobotânico das plantas medicinais do cerrado utilizadas pela população de Mossâmedes ( GO ). *Revista Brasileira de Farmacognosia Levantamento*, 13, pp.64–66.

Voeks, R.A. & Leony, A., 2004. Forgetting the forest : assessing medicinal plant erosion in eastern brazil I. , 58(Myers 1984).

Zucchi, M.R. et al., 2013. Levantamento etnobotânico de plantas medicinais na cidade de Ipameri - GO. *Revista Brasileira de Plantas Medicinais*, 15(2), pp.273–279.

List of sourced herbaria from CRIA species link.

| Cenargen |
| --- |
| CEPLAC |
| CPAP |
| EBDA |
| FEMACT |
| FURB |
| IBt |
| IEPA |
| IF |
| IFAM - CMZL |
| INPA |
| IPA |
| JBP |
| MBM |
| MBML |
| MCN/FZBRS |
| PUC-RIO |
| UDESC |
| UEFS |
| UEL |
| UEM |
| UESC |
| UFBA |
| UFC |
| UFERSA |
| UFES |
| UFG |
| UFJF |
| UFMA |
| UFMG |
| UFMS |
| UFOP |
| UFPB |
| UFPE |
| UFPI |
| UFPR |
| UFRN |
| UFRPE |
| UFRR |
| UFS |
| UFSC |
| UFSJ |
| UFU |
| UFV |
| UFVJM |
| UnB |
| UNEMAT |
| UNESC |
| UNESPIBB |
| UNESPRC |
| UNICAMP |
| UNICAP |
| UNIVASF |
| UTFPR |
