# Supplementary material for: Ewé: a web-based ethnobotanical database for storing and analysing data
Source: Database (Oxford). 2020 Feb 12;2020:baz144. doi: 10.1093/database/baz144 (PMC7015817; doi:10.1093/database/baz144)
Supplement: Suppplementary_information_3_baz144 [file suppplementary_information_3_baz144.docx]

**Suppplementary information 3. Species, therapeutic applications, plants parts used, modes of application and source for reports of medicinal use of Legumes in Brazil.**

| **Species** | **Therapeutic Applications (WHO-ICD10)** | **Plant Part Used** | **Modes of Application** | **Source^1^** |
| --- | --- | --- | --- | --- |
| *Abarema cochliocarpos (Gomes) Barneby & J.W.Grimes* |  |  |  | P.C. Gadelha Neto (2012) JBRJ |
| *Abarema cochliocarpos (Gomes) Barneby & J.W.Grimes* |  |  |  | G.B. Freitas (2006) JBRJ |
| *Abarema cochliocarpos (Gomes) Barneby & J.W.Grimes* |  |  |  | Rita Lima (2008) UFPB |
| *Abarema cochliocarpos (Gomes) Barneby & J.W.Grimes* |  |  |  | Xavier, L.P. (1969) UFPB |
| *Abarema cochliocarpos (Gomes) Barneby & J.W.Grimes* |  | Bark |  | Xavier, L.P. (1941) UFPB |
| *Abarema cochliocarpos (Gomes) Barneby & J.W.Grimes* |  |  |  | Gadelha Neto, P.C. (2012) UFPB |
| *Abarema cochliocarpos (Gomes) Barneby & J.W.Grimes* |  |  |  | Freitas, G.B. (2006) UFPB |
| *Abarema cochliocarpos (Gomes) Barneby & J.W.Grimes* |  |  |  | Oliveira, M. UFPE |
| *Abarema cochliocarpos (Gomes) Barneby & J.W.Grimes* | DFS, OTHER | Bark | Bath | Agra et al, 2008 |
| *Abarema cochliocarpos (Gomes) Barneby & J.W.Grimes* | DFS, OTHER | Bark, Seeds | Tea, Bath | Barbosa da Silva et al. 2012 |
| *Acacia bahiensis Benth.* |  |  |  | G.T. Soldati (2008) UFRPE |
| *Acacia bahiensis Benth.* | DFS, OTHER | Bark | Decoction | Agra et al, 2008 |
| *Acacia bahiensis Benth.* | DGS, DBI, DSS | Bark, Flowers, | Infusion | Almeida et al., 2006 |
| *Acacia farnesiana (L.) Willd.* |  |  |  | Xavier, L.P. (1942) UFPB |
| *Acacia farnesiana (L.) Willd.* | DDS, DEA, DNS, DMC | Flowers, Fruits, Bark, Roots, Leaves | Infusion With Water | Agra et al, 2008 |
| *Acacia farnesiana (L.) Willd.* | DDS, DRS, OTHER | Leaves, Flowers, Seeds | Infusion Or Alcoholic Infusion | Chaves & Barros, 2012 |
| *Acacia farnesiana (L.) Willd.* | DMC | Seeds, Leaves | Decoction Or Infusion | Costa & Mayworm, 2011 |
| *Acacia farnesiana (L.) Willd.* | DDS, DRS, OTHER | Fruit, Leaves | Tea, Alcoholic Infusion | Franco & Barros, 2006 |
| *Acacia farnesiana (L.) Willd.* |  |  |  | Guarim Neto & Morais, 2003 |
| *Acacia farnesiana (L.) Willd.* |  |  |  | Lucena et al., 2007 |
| *Acacia farnesiana (L.) Willd.* |  |  |  | Lucena et al., 2008 |
| *Acacia glomerosa Benth.* |  |  |  | Silva & Albuquerque, 2005 |
| *Acacia langsdorfii Benth.* | DRS, OTHER | Leaves, Bark | Infusion With Water | Agra et al, 2008 |
| *Acacia langsdorfii Benth.* | OTHER, DRS, DMC | Leaves, Bark | Tea | Franco & Barros, 2006 |
| *Acacia piauhiensis Benth.* | DMC | Leaves | Infusion With Water | Agra et al, 2008 |
| *Acacia tenuiflora (L.) Willd.* | DMC | Leaves | Infusion With Water | Agra et al, 2008 |
| *Acacia tenuiflora (L.) Willd.* |  |  |  | Albuquerque & Oliveira, 2007 |
| *Acacia tenuiflora (L.) Willd.* | DGS, DBI, DMC, OTHER | Leaves | Tea | Bratti et al., 2013 |
| *Acacia tenuiflora (L.) Willd.* |  |  |  | Silva & Albuquerque, 2005 |
| *Acosmium dasycarpum (Vogel) Yakovlev* |  |  |  | Amorozo, 2002 |
| *Acosmium dasycarpum (Vogel) Yakovlev* |  |  |  | Amorozo, 2004 |
| *Acosmium dasycarpum (Vogel) Yakovlev* |  |  |  | Guarim Neto & Morais, 2003 |
| *Acosmium dasycarpum (Vogel) Yakovlev* | OTHER |  |  | Lozano et al., 2014 |
| *Acosmium dasycarpum (Vogel) Yakovlev* | OTHER |  |  | Rodrigues, 2007 |
| *Acosmium dasycarpum (Vogel) Yakovlev* | DCS | Root | Infusion | Silva et al., 2010 |
| *Acosmium dasycarpum subsp. glabratum (Benth.) Yakovlev* |  |  |  | Kuhlmann, J.G. (1954) JBRJ |
| *Acosmium dasycarpum subsp. glabratum (Benth.) Yakovlev* |  |  |  | S.A. Cunha (2009) UFMS |
| *Acosmium dasycarpum subsp. glabratum (Benth.) Yakovlev* |  |  |  | Guarim Neto & Morais, 2003 |
| *Acosmium subelegans (Mohlenbr.) Yakovlev* |  |  |  | Cunha & Bortolotto, 2011 |
| *Acosmium subelegans (Mohlenbr.) Yakovlev* |  |  |  | Guarim Neto & Morais, 2003 |
| *Acosmium subelegans (Mohlenbr.) Yakovlev* | DFS | Root | Tea | Pasa et al, 2011 |
| *Acosmium subelegans (Mohlenbr.) Yakovlev* | IPD, DBI | Root | Tea, Wine | Pinto et al., 2013 |
| *Adenanthera pavonina L.* | DMC |  |  | Montenegro, N.F. (1985) UFPB |
| *Aeschynomene mollicula Kunth* | OTHER |  |  | Rodrigues, 2007 |
| *Albizia inundata (Mart.) Barneby & J.W.Grimes* |  |  |  | Brandão et al, 2012 |
| *Albizia niopoides (Benth.) Burkart* |  |  |  | Silva & Proença, 2008 |
| *Albizia polycephala (Benth.) Killip* |  |  |  | Espinola, M.C. (1942) UFPB |
| *Albizia polycephala (Benth.) Killip* | DMC | Leaves | Infusion With Water | Agra et al, 2008 |
| *Amburana cearensis (Allemao) A.C.Sm.* |  |  |  | Sobrinho, J.S. (1965) IPA |
| *Amburana cearensis (Allemao) A.C.Sm.* | DRS | Bark | Tea | Mendes, I. da C.A. (1992) JBRJ |
| *Amburana cearensis (Allemao) A.C.Sm.* |  |  |  | Mendes, I. da C.A. (1992) JBRJ |
| *Amburana cearensis (Allemao) A.C.Sm.* |  |  |  | Pereira, B.A.S. (1991) JBRJ |
| *Amburana cearensis (Allemao) A.C.Sm.* |  |  |  | Oliveira, O.F. de (1979) UFERSA |
| *Amburana cearensis (Allemao) A.C.Sm.* |  |  |  | Lima-Verde, L.W. (1996) UFERSA |
| *Amburana cearensis (Allemao) A.C.Sm.* |  |  |  | S.A. Cunha (2009) UFMS |
| *Amburana cearensis (Allemao) A.C.Sm.* |  |  |  | Almeida, C. (2006) UFPE |
| *Amburana cearensis (Allemao) A.C.Sm.* | DRS, ENM | Bark, Seeds | Decoction With Water, Syrup, Powder, Inhale | Agra et al, 2007 |
| *Amburana cearensis (Allemao) A.C.Sm.* | DRS, ENM, DGS | Bark | Decoction | Agra et al, 2007 |
| *Amburana cearensis (Allemao) A.C.Sm.* | DRS, ENM | Bark, Seeds | Decoction With Water, Syrup, Powder, Inhale | Agra et al, 2008 |
| *Amburana cearensis (Allemao) A.C.Sm.* |  |  |  | Albuquerque & Oliveira, 2007 |
| *Amburana cearensis (Allemao) A.C.Sm.* | DRS, DDS | Stem, Seed |  | Albuquerque et al, 2007 |
| *Amburana cearensis (Allemao) A.C.Sm.* | DFS, DDS, OTHER | Bark, Seeds, Leaves | Tea | Barbosa da Silva et al. 2012 |
| *Amburana cearensis (Allemao) A.C.Sm.* | DRS, DCS | Fruit, Seeds, Bark | Bath | Cartaxo et al, 2010 |
| *Amburana cearensis (Allemao) A.C.Sm.* | DCS | Seeds | Tea | Castro et al., 2011 |
| *Amburana cearensis (Allemao) A.C.Sm.* | DRS, ENM, DFS | Bark, Seeds | Alcoholic Infusion | Chaves & Barros, 2012 |
| *Amburana cearensis (Allemao) A.C.Sm.* | DRS, DMC, DFS | Bark |  | Conceição et al, 2011 |
| *Amburana cearensis (Allemao) A.C.Sm.* |  |  |  | Cunha & Bortolotto, 2011 |
| *Amburana cearensis (Allemao) A.C.Sm.* | DRS | Bark | Infusion | de Albuquerque, 2006 |
| *Amburana cearensis (Allemao) A.C.Sm.* | OTHER | Seeds | Tea | Fita et al., 2010 |
| *Amburana cearensis (Allemao) A.C.Sm.* | DRS | Bark | Decoction | Freitas et al, 2012 |
| *Amburana cearensis (Allemao) A.C.Sm.* |  |  |  | Guarim Neto & Morais, 2003 |
| *Amburana cearensis (Allemao) A.C.Sm.* |  |  |  | Kainer & Duryea, 1992 |
| *Amburana cearensis (Allemao) A.C.Sm.* | DRS |  |  | Lozano et al., 2014 |
| *Amburana cearensis (Allemao) A.C.Sm.* | DRS | Bark | Tea | Morais et al, 2005 |
| *Amburana cearensis (Allemao) A.C.Sm.* | DRS |  |  | Nunes et al, 2003 |
| *Amburana cearensis (Allemao) A.C.Sm.* | DRS, OTHER | Bark, Seeds, Leaves |  | Oliveira et al., 2010 |
| *Amburana cearensis (Allemao) A.C.Sm.* | DDS | Bark | Tea | Pinto et al., 2013 |
| *Amburana cearensis (Allemao) A.C.Sm.* |  |  |  | Roque et al., 2010 |
| *Amburana cearensis (Allemao) A.C.Sm.* |  |  |  | Silva & Albuquerque, 2005 |
| *Amburana cearensis (Allemao) A.C.Sm.* | DRS, OTHER | Bark | Syrup | Silva & Freire, 2010 |
| *Amburana cearensis (Allemao) A.C.Sm.* |  |  |  | Silva et al., 2014 |
| *Amburana cearensis (Allemao) A.C.Sm.* | DDS, DRS, DSS |  |  | Souza & Felfilli, 2006 |
| *Anadenanthera colubrina (Vell.) Brenan* | DRS, DMC | Bark | Syrup | Mattos Silva, L.A. (1988) JBRJ |
| *Anadenanthera colubrina (Vell.) Brenan* |  |  |  | Emperaire, L. (1984) JBRJ |
| *Anadenanthera colubrina (Vell.) Brenan* |  |  |  | U.M. Resende (2001) UFMS |
| *Anadenanthera colubrina (Vell.) Brenan* |  |  |  | Almeida, C. (2006) UFPE |
| *Anadenanthera colubrina (Vell.) Brenan* | DRS | Bark | Alcoholic Infusion | Agra et al, 2007 |
| *Anadenanthera colubrina (Vell.) Brenan* | DRS | Bark | Alcoholic Infusion, Infusion With Water | Agra et al, 2007 |
| *Anadenanthera colubrina (Vell.) Brenan* | DRS | Bark | Alcoholic Infusion, Infusion With Water | Agra et al, 2008 |
| *Anadenanthera colubrina (Vell.) Brenan* |  |  |  | Albuquerque & Oliveira, 2007 |
| *Anadenanthera colubrina (Vell.) Brenan* | ENM, DRS | Stem |  | Albuquerque et al, 2007 |
| *Anadenanthera colubrina (Vell.) Brenan* |  | Bark |  | Alencar et al., 2010 |
| *Anadenanthera colubrina (Vell.) Brenan* | DRS | Bark | Decoction | Almeida et al., 2006 |
| *Anadenanthera colubrina (Vell.) Brenan* | DRS | Bark | Syrup | Barbosa da Silva et al. 2012 |
| *Anadenanthera colubrina (Vell.) Brenan* | DRS | Leaves |  | Brandao et al, 2008 |
| *Anadenanthera colubrina (Vell.) Brenan* | DRS, DDS, NEO, OTHER |  |  | Cartaxo et al, 2010 |
| *Anadenanthera colubrina (Vell.) Brenan* | DRS | Bark, Leaves | Tea, Syrup | Castro et al., 2011 |
| *Anadenanthera colubrina (Vell.) Brenan* | DRS |  |  | Giraldi & Hanazaki, 2010 |
| *Anadenanthera colubrina (Vell.) Brenan* | DRS | Bark | Tea, Syrup | Gomes & Bandeira, 2012 |
| *Anadenanthera colubrina (Vell.) Brenan* |  |  |  | Guarim Neto & Morais, 2003 |
| *Anadenanthera colubrina (Vell.) Brenan* | OTHER | Bark | Bath | Jesus et al., 2009 |
| *Anadenanthera colubrina (Vell.) Brenan* |  |  |  | Lucena et al., 2007 |
| *Anadenanthera colubrina (Vell.) Brenan* |  |  |  | Lucena et al., 2008 |
| *Anadenanthera colubrina (Vell.) Brenan* | DRS | Bark | Syrup | Oliveira et al., 2010 |
| *Anadenanthera colubrina (Vell.) Brenan* |  |  |  | Roque et al., 2010 |
| *Anadenanthera colubrina (Vell.) Brenan* |  |  |  | Silva & Albuquerque, 2005 |
| *Anadenanthera colubrina (Vell.) Brenan* | OTHER | Bark | Infusion | Silva & Freire, 2010 |
| *Anadenanthera colubrina (Vell.) Brenan* |  |  |  | Silva et al., 2014 |
| *Anadenanthera colubrina var. cebil (Griseb.) Altschul* |  |  |  | Barbosa, J.I.S.; Silva, A. .G.; Silva, M. .V.; Santana, T.; Silva, A. .G.; Silva, M. .V.; Santana, T. (2009) IPA |
| *Anadenanthera colubrina var. cebil (Griseb.) Altschul* |  |  |  | Oliveira, O.F. de (1972) UFERSA |
| *Anadenanthera colubrina var. cebil (Griseb.) Altschul* |  |  |  | Oliveira, O.F. de (1972) UFERSA |
| *Anadenanthera colubrina var. cebil (Griseb.) Altschul* |  |  |  | Netto, D.A.M. (1987) UNICAMP |
| *Anadenanthera colubrina var. cebil (Griseb.) Altschul.* | DRS, DDS, OTHER |  |  | Desmarchelier et al., 1999 |
| *Anadenanthera colubrina var. cebil (Griseb.) Altschul.* | DRS, DMC, OTHER | Bark | Alcoholic Infusion | Franco & Barros, 2006 |
| *Anadenanthera colubrina var. cebil (Griseb.) Altschul.* | DRS | Bark | Syrup | Guarim-Neto, 2006 |
| *Anadenanthera colubrina var. cebil (Griseb.) Altschul.* |  |  |  | Silva e Andrade, 2005 |
| *Anadenanthera peregrina (L.) Speg.* |  |  |  | S.A. Cunha (2009) UFMS |
| *Anadenanthera peregrina (L.) Speg.* |  |  |  | A.S. Penha (2004) UFMS |
| *Anadenanthera peregrina (L.) Speg.* |  |  |  | A.S. Penha (2004) UFMS |
| *Anadenanthera peregrina (L.) Speg.* |  |  |  | Cunha & Bortolotto, 2011 |
| *Anadenanthera peregrina (L.) Speg.* |  |  |  | Guarim Neto & Morais, 2003 |
| *Anadenanthera peregrina* | DRS |  |  | Pasa et al, 2011 |
| *Anadenanthera peregrina var. falcata (Benth.) Altschul* | DSS, DEA | Bark | Infusion | Bueno et al., 2005 |
| *Anadenanthera peregrina var. falcata (Benth.) Altschul* | DRS, DNS, DMC, OTHER | Bark |  | Conceição et al, 2011 |
| *Anadenanthera peregrina var. falcata (Benth.) Altschul* |  |  |  | Guarim Neto & Morais, 2003 |
| *Anadenanthera peregrina var. falcata (Benth.) Altschul* | DFS |  |  | Souza & Felfilli, 2006 |
| *Anadenanthera peregrina var. falcata (Benth.) Altschul* | DRS | Leaves | Decoction | Vila Verde et al, 2003 |
| *Andira cuiabensis Benth.* |  |  |  | Guarim Neto & Morais, 2003 |
| *Andira cuiabensis Benth.* |  |  |  | Guarim Neto & Pasa, 2009 |
| *Andira cuiabensis Benth.* | ENM | Root | Tea | Macedo & Ferreira, 2005 |
| *Andira fraxinifolia Benth.* | IPD | Bark | Powder | Brandao et al, 2012 |
| *Andira fraxinifolia Benth.* | IPD | Bark | Drink | Brandão et al, 2012 |
| *Andira fraxinifolia Benth.* |  |  |  | Guarim Neto & Morais, 2003 |
| *Andira fraxinifolia Benth.* |  |  |  | Silva e Andrade, 2005 |
| *Andira humilis Mart. ex Benth.* |  |  |  | Rita Lima (2008) UFPB |
| *Andira humilis Mart. ex Benth.* |  |  |  | Brandão et al, 2012 |
| *Andira humilis Mart. ex Benth.* | ENM, IPD | Root |  | Conceição et al, 2011 |
| *Andira humilis Mart. ex Benth.* | IPD | Root | Tea | Gomes & Bandeira, 2012 |
| *Andira humilis Mart. ex Benth.* |  |  |  | Guarim Neto & Morais, 2003 |
| *Andira humilis Mart. ex Benth.* | DFS, DRS, IPD |  |  | Souza, 2007 |
| *Andira inermis (Wright) DC.* |  |  |  | Xavier, L.P. (1951) UFPB |
| *Andira inermis (Wright) DC.* | ENM | Root | Tea | Macedo & Ferreira, 2005 |
| *Andira laurifolia Benth.* | IPD, OTHER | Fruits, Bark |  | Hirschmann & Arias, 1990 |
| *Andira vermifuga Benth.* |  |  |  | Guarim Neto & Morais, 2003 |
| *Apuleia leiocarpa (Vogel) J.F.Macbr.* | DRS | Bark | Infusion | Battisti et al, 2013 |
| *Aspalathus linearis (Burm.f.) R.Dahlgren* | ENM |  | Tea | Dornas et al., 2008 |
| *Bauhinia acuruana Moric.* |  |  |  | Amorozo, 2002 |
| *Bauhinia bicolor D.Dietr.* | ENM |  |  | Jangoux, J.J. (1981) INPA |
| *Bauhinia bicolor D.Dietr.* |  |  |  | A. Pott (2001) UFMS |
| *Bauhinia blakeana Dunn.* |  |  |  | A. R. Freitas (2012) UFMS |
| *Bauhinia brevipes Vogel* |  |  |  | ROBERTO FONTES VIEIRA (1991) JBRJ |
| *Bauhinia cheilantha (Bong.) Steud.* |  |  |  | Agra, M.F.; Silva, M.G.; Silva, M.G. (1993) IPA |
| *Bauhinia cheilantha (Bong.) Steud.* | DMC, ENM | Bark | Infusion | Nelson, B.W. (1980) JBRJ |
| *Bauhinia cheilantha (Bong.) Steud.* |  |  |  | Pott, A. (1995) JBRJ |
| *Bauhinia cheilantha (Bong.) Steud.* |  |  |  | S.R. Xavier-JÚnior (2012) JBRJ |
| *Bauhinia cheilantha (Bong.) Steud.* |  |  |  | Oliveira, F.C.S (2008) JBRJ |
| *Bauhinia cheilantha (Bong.) Steud.* |  |  |  | V.J. Pott (2001) UFMS |
| *Bauhinia cheilantha (Bong.) Steud.* |  |  |  | V.J. Pott (2001) UFMS |
| *Bauhinia cheilantha (Bong.) Steud.* |  |  |  | V.J. Pott (2001) UFMS |
| *Bauhinia cheilantha (Bong.) Steud.* |  |  |  | A.S. Penha (2004) UFMS |
| *Bauhinia cheilantha (Bong.) Steud.* |  |  |  | A.S. Penha (2004) UFMS |
| *Bauhinia cheilantha (Bong.) Steud.* | ENM |  |  | Sousa, M.A. (1982) UFPB |
| *Bauhinia cheilantha (Bong.) Steud.* |  |  |  | Rocha, E.A. (2004) UFPB |
| *Bauhinia cheilantha (Bong.) Steud.* |  |  |  | Espinola, M.C. (1942) UFPB |
| *Bauhinia cheilantha (Bong.) Steud.* |  |  |  | Bortolotto, I.M.; Ishii, I.H.; Assis, E.M.; Rosso, C.; Magalhaes, V.; Damasceno Junior, G.A.; G.A (2004) UNICAMP |
| *Bauhinia cheilantha (Bong.) Steud.* | ENM | Bark | Infusion | Agra et al, 2007 |
| *Bauhinia cheilantha (Bong.) Steud.* | ENM | Leaves, Bark | Tea | Agra et al, 2007 |
| *Bauhinia cheilantha (Bong.) Steud.* | ENM | Leaves, Bark | Tea | Agra et al, 2008 |
| *Bauhinia cheilantha (Bong.) Steud.* | NEO | Leaves | Tea | Aguiar & Barros, 2012 |
| *Bauhinia cheilantha (Bong.) Steud.* |  |  |  | Albuquerque & Oliveira, 2007 |
| *Bauhinia cheilantha (Bong.) Steud.* | ENM, DMC, DRS, DNS, ENM | Leaves, Seed, Flower, Root, Bark |  | Albuquerque et al, 2007 |
| *Bauhinia cheilantha (Bong.) Steud.* |  | Leaves |  | Alencar et al., 2010 |
| *Bauhinia cheilantha (Bong.) Steud.* | ENM, DBI, DNS | Leaves, Bark, Seed | Decoction | Almeida et al., 2006 |
| *Bauhinia cheilantha (Bong.) Steud.* | ENM, DGS | Leaves, Bark |  | Campelo & Ramalho, 1989 |
| *Bauhinia cheilantha (Bong.) Steud.* | ENM, OTHER | Leaves, Bark | Decoction, Alcoholic Infusion | Chaves & Barros, 2012 |
| *Bauhinia cheilantha (Bong.) Steud.* | ENM | Leaves | Infusion | Costa & Mayworm, 2011 |
| *Bauhinia cheilantha* | ENM, DRS, other |  |  | de Albuquerque, 2006 |
| *Bauhinia cheilantha (Bong.) Steud.* | ENM, DDS | Leaves, Bark | Tea, Syrup | Freitas et al, 2012 |
| *Bauhinia cheilantha (Bong.) Steud.* |  |  |  | Lucena et al., 2007 |
| *Bauhinia cheilantha (Bong.) Steud.* |  |  |  | Lucena et al., 2008 |
| *Bauhinia cheilantha (Bong.) Steud.* |  |  |  | Roque et al., 2010 |
| *Bauhinia cheilantha (Bong.) Steud.* |  |  |  | Silva & Albuquerque, 2005 |
| *Bauhinia cheilantha (Bong.) Steud.* | DRS | Root | Syrup | Silva & Freire, 2010 |
| *Bauhinia cheilantha (Bong.) Steud.* |  |  |  | Silva et al., 2014 |
| *Bauhinia corniculata Benth.* |  |  |  | Gadelha Neto, P.C. (2012) UFPB |
| *Bauhinia cupulata Benth.* | DDS |  |  | Navarro, A. (1942) UFPB |
| *Bauhinia dubia Vogel* | DGS | Leaves | Tea | Monteles & Pinheiro, 2007 |
| *Bauhinia dubia Vogel* | ENM, DCS, | Leaves, Flowers |  | Oliveira et al., 2010 |
| *Bauhinia flexuosa Moric.* | DGS, DDS, OTHER | Bark | Alcoholic Infusion | Franco & Barros, 2006 |
| *Bauhinia forficata Link* |  |  |  | Rocha, E.A. (2004) UFPE |
| *Bauhinia forficata Link* |  |  |  | Rocha, E.A. (2004) UFPE |
| *Bauhinia forficata Link* |  |  |  | Almeida, C. (2006) UFPE |
| *Bauhinia forficata Link* |  |  |  | Almeida, C. (2006) UFPE |
| *Bauhinia forficata Link* | ENM | Leaves, Bark | Tea | Agra et al, 2007 |
| *Bauhinia forficata Link* | ENM | Leaves, Bark | Tea | Agra et al, 2008 |
| *Bauhinia forficata Link* |  |  |  | Althaus-ottmann et al, 2011 |
| *Bauhinia forficata Link* |  | Leaves |  | Azevedo & Kruel, 2007 |
| *Bauhinia forficata Link* | ENM, DBI | Leaves | Tea | Battisti et al, 2013 |
| *Bauhinia forficata Link* | ENM | Leaves | Tea | Coelho-Ferreira, 2009 |
| *Bauhinia forficata Link* | ENM, DDS, DGS | Leaves, Flower |  | Conceição et al, 2011 |
| *Bauhinia forficata Link* | ENM | Leaves | Infusion | Costa & Mayworm, 2011 |
| *Bauhinia forficata Link* |  |  |  | Eichemberg et al., 2009 |
| *Bauhinia forficata Link* | ENM | Leaves, Bark | Tea | Franco & Barros, 2006 |
| *Bauhinia forficata Link* | ENM, DCS, DGS |  |  | Giraldi & Hanazaki, 2010 |
| *Bauhinia forficata Link* | ENM | Leaves | Tea | Hirschmann & Arias, 1990 |
| *Bauhinia forficata Link* | ENM | Leaves | Tea | Oliveira & Menini Neto, 2012 |
| *Bauhinia forficata Link* | ENM | Leaves | Decoction | Oliveira et al., 2010 |
| *Bauhinia forficata Link* | ENM, DGS, DRS, DCS |  |  | Ritter et al., 2002 |
| *Bauhinia forficata Link* | ENM, NEO, DCS, DGS | Leaves | Infusion Or Decoction | Santos et al, 2008 |
| *Bauhinia forficata Link* | ENM, DGS, DMC |  |  | Souza & Felfilli, 2006 |
| *Bauhinia forficata Link* | ENM | Leaves, Flowers, Bark | Tea, Alcoholic Infusion | Trojan-Rodrigues et al, 2012 |
| *Bauhinia forficata subsp. pruinosa (Vogel) Fortunato & Wunderlin* | ENM, DGS, DCS | Leaves |  | Meretika et al., 2010 |
| *Bauhinia glabra Jacq.* |  |  |  | Miranda, B.; Ataide, M.; Alberto, C.; Mansur, R.J.; Soares, T.J.; Lyra, R.P.; Ataide, M.; Alberto, C.; Mansur, R.J.; Soares, T.J.; Lyra, R.P. (1984) IPA |
| *Bauhinia glabra Jacq.* |  |  |  | A. Pott (2002) UFMS |
| *Bauhinia glabra Jacq.* |  |  |  | A. Pott (2003) UFMS |
| *Bauhinia glabra Jacq.* |  |  |  | A. Pott (2001) UFMS |
| *Bauhinia glabra Jacq.* | ENM, DBI, DCS |  |  | Londoño, P.A. (2010) UFPE |
| *Bauhinia glabra Jacq.* |  |  |  | Amorozo, 2002 |
| *Bauhinia glabra Jacq.* | DGS |  |  | Freitas et al, 2013 |
| *Bauhinia glabra Jacq.* |  |  |  | Guarim Neto & Morais, 2003 |
| *Bauhinia glabra Jacq.* |  |  |  | Guarim Neto & Pasa, 2009 |
| *Bauhinia glabra Jacq.* | DDS | Root | With Water | Guarim-Neto, 2006 |
| *Bauhinia glabra Jacq.* | ENM | Root | Tea | Macedo & Ferreira, 2005 |
| *Bauhinia guianensis Aubl.* | DCS | Stem | Tea, Bath | Coelho-Ferreira, 2009 |
| *Bauhinia guianensis Aubl.* |  |  |  | Guarim Neto & Morais, 2003 |
| *Bauhinia guianensis Aubl.* | DEM |  |  | Lozano et al., 2014 |
| *Bauhinia guianensis Aubl.* | ENM | Bark | Alcoholic Infusion | Monteles & Pinheiro, 2007 |
| *Bauhinia guianensis var. splendens (Kunth) Amshoff* |  |  |  | A. Pott (2001) UFMS |
| *Bauhinia guianensis var. splendens (Kunth) Amshoff* |  |  |  | Guarim Neto & Morais, 2003 |
| *Bauhinia kunthiana Vogel* |  |  |  | Shanley & Rosa, 2004 |
| *Bauhinia leptantha Malme* | DRS, ENM, IPD, OTHER | Root, Bark, Leaves | Tea, Bath | Cortas, A. (1991) INPA |
| *Bauhinia leptantha Malme* | DGS |  |  | Aline Redondo Martins (2002) UNESPSJRP |
| *Bauhinia longicuspis Benth.* |  |  |  | F. A. Sobrinho (2006) JBRJ |
| *Bauhinia longifolia (Bong.) Steud.* |  |  |  | Janssen, A.S. (1980) INPA |
| *Bauhinia longifolia (Bong.) Steud.* | DDS |  |  | Janssen, A. (1980) JBRJ |
| *Bauhinia longifolia (Bong.) Steud.* |  |  |  | Pott, A. (1989) JBRJ |
| *Bauhinia longifolia (Bong.) Steud.* |  |  |  | Leitao, F. (2005) JBRJ |
| *Bauhinia longifolia (Bong.) Steud.* |  |  |  | E.C. Bertazzoni UFMS |
| *Bauhinia longifolia (Bong.) Steud.* |  |  |  | V.J. Pott (2001) UFMS |
| *Bauhinia longifolia (Bong.) Steud.* | ENM | Leaves | Tea | Agra et al, 2007 |
| *Bauhinia longifolia (Bong.) Steud.* | ENM | Leaves | Tea | Agra et al, 2008 |
| *Bauhinia microstachya (Raddi) J.F.Macbr.* | IPD |  |  | Kinupp, V.F.; Ming, L.C.; Vieira, D.A.F.; Araújo, A.A. (2009) FEMACT |
| *Bauhinia microstachya (Raddi) J.F.Macbr.* |  |  |  | A. Pott (1995) UFMS |
| *Bauhinia microstachya (Raddi) J.F.Macbr.* |  | Leaves |  | Azevedo & Kruel, 2007 |
| *Bauhinia microstachya (Raddi) J.F.Macbr.* | ENM | Leaves | Tea, Alcoholic Infusion | Trojan-Rodrigues et al, 2012 |
| *Bauhinia mollis (Bong.) D.Dietr.* | ENM |  |  | Cortas, A. (1991) INPA |
| *Bauhinia mollis (Bong.) D.Dietr.* |  |  |  | E.P. Seleme (2006) UFMS |
| *Bauhinia mollis (Bong.) D.Dietr.* |  |  |  | E.P. Seleme (2007) UFMS |
| *Bauhinia mollis (Bong.) D.Dietr.* |  |  |  | A. Pott (2003) UFMS |
| *Bauhinia mollis (Bong.) D.Dietr.* |  |  |  | Amorozo, 2002 |
| *Bauhinia mollis (Bong.) D.Dietr.* |  |  |  | Cunha & Bortolotto, 2011 |
| *Bauhinia monandra Kurz* | ENM |  |  | Hage, J.L. (1988) JBRJ |
| *Bauhinia monandra Kurz* |  |  |  | A. Pott (2002) UFMS |
| *Bauhinia monandra Kurz* |  |  |  | V.J. Pott (2001) UFMS |
| *Bauhinia monandra Kurz* | ENM | Bark | Tea | Agra et al, 2007 |
| *Bauhinia monandra Kurz* | ENM | Bark | Tea | Agra et al, 2008 |
| *Bauhinia monandra Kurz* | ENM | Leaves | Infusion | Albuquerque, 2001 |
| *Bauhinia nitida Benth.* |  |  |  | Guarim Neto & Morais, 2003 |
| *Bauhinia nitida Benth.* | ENM, DGS | Leaves | Tea | Pasa et al, 2011 |
| *Bauhinia pentandra (Bong.) Steud.* |  |  |  | Leitao, F. (2005) JBRJ |
| *Bauhinia pentandra (Bong.) Steud.* |  |  |  | Silva, I.M. (2005) JBRJ |
| *Bauhinia pentandra (Bong.) Steud.* | ENM |  |  | Neto, E.T. (1982) UNICAMP |
| *Bauhinia pentandra (Bong.) Steud.* | ENM | Bark | Tea | Agra et al, 2007 |
| *Bauhinia pentandra (Bong.) Steud.* | ENM | Bark | Tea | Agra et al, 2008 |
| *Bauhinia pentandra (Bong.) Steud.* |  |  |  | Roque et al., 2010 |
| *Bauhinia pulchella Benth.* | ENM | Bark | Tea | Agra et al, 2007 |
| *Bauhinia pulchella Benth.* | ENM | Bark | Tea | Agra et al, 2008 |
| *Bauhinia pulchella Benth.* | ENM | Bark | Tea | Aguiar & Barros, 2012 |
| *Bauhinia pulchella Benth.* | OTHER |  |  | Rodrigues, 2007 |
| *Bauhinia purpurea L.* | ENM | Leaves | Tea | Agra et al, 2007 |
| *Bauhinia purpurea L.* | ENM | Leaves | Tea | Agra et al, 2008 |
| *Bauhinia purpurea L.* | ENM | Leaves | Infusion | Albuquerque, 2001 |
| *Bauhinia purpurea L.* | ENM |  |  | Conde et al., 2014 |
| *Bauhinia radiata Vell.* |  |  |  | S.A. Cunha (2008) UFMS |
| *Bauhinia radiata Vell.* |  |  |  | V.J. Pott (2007) UFMS |
| *Bauhinia radiata Vell.* |  |  |  | A. Pott (2001) UFMS |
| *Bauhinia radiata Vell.* |  | Leaves |  | Azevedo & Kruel, 2007 |
| *Bauhinia radiata Vell.* | ENM | Leaves |  | Christo et al., 2010 |
| *Bauhinia rufa (Bong.) Steud.* |  |  |  | A. R. Freitas (2012) UFMS |
| *Bauhinia rufa (Bong.) Steud.* |  |  |  | Conceicao, G.M. (1998) UNICAMP |
| *Bauhinia rufa (Bong.) Steud.* |  |  |  | Amorozo, 2002 |
| *Bauhinia rufa (Bong.) Steud.* |  |  |  | Guarim Neto & Morais, 2003 |
| *Bauhinia rufa (Bong.) Steud.* |  |  |  | Guarim Neto & Pasa, 2009 |
| *Bauhinia rufa (Bong.) Steud.* | ENM | Leaves, Bark | Tea | Macedo & Ferreira, 2005 |
| *Bauhinia rufa (Bong.) Steud.* | DCS | Leaves | Infusion | Silva et al., 2010 |
| *Bauhinia rutilans Benth.* |  |  |  | Shanley & Rosa, 2004 |
| *Bauhinia subclavata Benth.* | DDS | Leaves, Flowers | Tea | Gomes & Bandeira, 2012 |
| *Bauhinia ungulata L.* | ENM |  |  | Nelson, B.W. (1980) INPA |
| *Bauhinia ungulata L.* |  |  |  | Skorupa, L.A. (1988) JBRJ |
| *Bauhinia ungulata L.* |  |  |  | Dias, F.F. (2006) UEL |
| *Bauhinia ungulata L.* | ENM |  |  | D.W. Mendonca (2004) UFMS |
| *Bauhinia ungulata L.* |  |  |  | B.F. Leonel (2006) UFMS |
| *Bauhinia ungulata L.* |  |  |  | F.F. Dias (2006) UFMS |
| *Bauhinia ungulata L.* |  |  |  | A. Pott (2002) UFMS |
| *Bauhinia ungulata L.* |  |  |  | A. Pott (2005) UFMS |
| *Bauhinia ungulata L.* |  |  |  | A. Pott (2001) UFMS |
| *Bauhinia ungulata L.* |  |  |  | A. Pott (2002) UFMS |
| *Bauhinia ungulata L.* |  |  |  | Pereira, L.G. (1994) UFPE |
| *Bauhinia ungulata L.* | ENM | Leaves | Tea | Agra et al, 2007 |
| *Bauhinia ungulata L.* | ENM | Leaves | Tea | Agra et al, 2008 |
| *Bauhinia ungulata L.* |  |  |  | Amorozo, 2002 |
| *Bauhinia ungulata L.* | ENM, OTHER | Leaves, Bark | Decoction, Alcoholic Infusion | Chaves & Barros, 2012 |
| *Bauhinia ungulata L.* |  |  |  | Fernandes et al., 2014 |
| *Bauhinia ungulata L.* |  |  |  | Guarim Neto & Morais, 2003 |
| *Bauhinia ungulata L.* | ENM | Leaves | Tea | Morais et al, 2005 |
| *Bauhinia ungulata L.* | ENM, DCS, DDS | Bark, Leaves, Flowers |  | Oliveira et al., 2010 |
| *Bauhinia variegata L.* | ENM |  |  | S.R. Xavier JÚnior (2012) JBRJ |
| *Bauhinia variegata L.* |  |  |  | S.R. Xavier-JÚnior (2012) JBRJ |
| *Bauhinia variegata L.* |  |  |  | S.R. Xavier-JÚnior (2012) JBRJ |
| *Bauhinia variegata L.* |  |  |  | R.F. VIEIRA (1986) JBRJ |
| *Bauhinia variegata L.* |  |  |  | Biavatti, M.W. (1997) JBRJ |
| *Bauhinia variegata L.* |  |  |  | Leitao, F. (2005) JBRJ |
| *Bauhinia variegata L.* |  |  |  | Patzlaff,R. (2000) JBRJ |
| *Bauhinia variegata L.* | DGS |  |  | Equipe J. H. (1992) UFMS |
| *Bauhinia variegata L.* | ENM |  |  | Albuquerque, U.P. (1998) UFPE |
| *Bauhinia variegata L.* |  |  |  | Barros, A.E.; Silva (1994) UFPE |
| *Bauhinia variegata L.* |  | Leaves |  | Azevedo & Kruel, 2007 |
| *Bauhinia variegata L.* | ENM |  |  | Lozano et al., 2014 |
| *Bauhinia variegata L.* | ENM | Leaves | Tea | Pinto et al., 2013 |
| *Bauhinia variegata L.* | ENM | Leaves | Tea | Trojan-Rodrigues et al, 2012 |
| *Bowdichia nitida Benth.* |  |  |  | Guarim Neto & Morais, 2003 |
| *Bowdichia virgilioides Kunth* |  |  |  | Sobrinho, J.S. (1965) IPA |
| *Bowdichia virgilioides Kunth* | DRS |  |  | R.W.N. Borges JBRJ |
| *Bowdichia virgilioides Kunth* |  |  |  | D. Lima (1998) JBRJ |
| *Bowdichia virgilioides Kunth* | DMC |  |  | Xavier, L.P. (1942) UFPB |
| *Bowdichia virgilioides Kunth* |  |  |  | J.S. Sobrinho (1965) UFRPE |
| *Bowdichia virgilioides Kunth* | DFS | Bark | Bath | Agra et al, 2007 |
| *Bowdichia virgilioides Kunth* | DFS | Bark | Bath | Agra et al, 2008 |
| *Bowdichia virgilioides Kunth* | ENM, DMC, DNS | Bark |  | Albuquerque et al, 2007 |
| *Bowdichia virgilioides Kunth* |  |  |  | Amorozo, 2002 |
| *Bowdichia virgilioides Kunth* | DDS | Bark, Seed | Tincture | Borba & Macedo, 2006 |
| *Bowdichia virgilioides Kunth* |  |  |  | Botrel et al., 2006 |
| *Bowdichia virgilioides Kunth* | DSS | Rinds |  | Brandao et al, 2008 |
| *Bowdichia virgilioides Kunth* | DSS, OTHER | Bark |  | Breitbach et al., 2013 |
| *Bowdichia virgilioides Kunth* | DDS | Bark, Resin |  | Campelo & Ramalho, 1989 |
| *Bowdichia virgilioides Kunth* | DRS, OTHER | Bark, Seeds | Alcoholic Infusion | Chaves & Barros, 2012 |
| *Bowdichia virgilioides Kunth* | IPD, DRS | Seed |  | Conceição et al, 2011 |
| *Bowdichia virgilioides Kunth* | OTHER, DMC, DGS | Bark | Alcoholic Infusion | Franco & Barros, 2006 |
| *Bowdichia virgilioides Kunth* | DFS, OTHER | Bark |  | Gazzaneo et al., 2005 |
| *Bowdichia virgilioides Kunth* | DCS | Bark | Tea | Gomes & Bandeira, 2012 |
| *Bowdichia virgilioides Kunth* |  |  |  | Guarim Neto & Morais, 2003 |
| *Bowdichia virgilioides Kunth* |  |  |  | Guarim Neto & Pasa, 2009 |
| *Bowdichia virgilioides Kunth* | DRS | Seeds | Tea | Guarim-Neto, 2006 |
| *Bowdichia virgilioides Kunth* | DMC |  |  | Lozano et al., 2014 |
| *Bowdichia virgilioides Kunth* | ENM | Root | Tea | Macedo & Ferreira, 2005 |
| *Bowdichia virgilioides Kunth* | DRS | Fruit | Tea | Pasa et al, 2011 |
| *Bowdichia virgilioides Kunth* |  |  |  | Silva e Andrade, 2005 |
| *Bowdichia virgilioides f. major (Mart.) Yakovlev* | DDS, DMC, DRS | Seed, Bark |  | Freitas et al, 2012 |
| *Caesalpinia bracteosa Tul.* |  |  |  | Sobrinho, J.S. (1965) IPA |
| *Caesalpinia bracteosa Tul.* |  |  |  | Freitas, M.L. de (1993) UFERSA |
| *Caesalpinia bracteosa Tul.* |  |  |  | Guimaraes, W.R. (1974) UFERSA |
| *Caesalpinia bracteosa Tul.* |  |  |  | Lima-Verde, L.W. (1996) UFERSA |
| *Caesalpinia bracteosa Tul.* |  |  |  | J.S. Sobrinho (1965) UFRPE |
| *Caesalpinia bracteosa Tul.* | OTHER | Fruit, Bark | Decotion With Water | Agra et al, 2007 |
| *Caesalpinia bracteosa Tul.* | OTHER | Fruit, Bark | Decotion With Water | Agra et al, 2008 |
| *Caesalpinia bracteosa Tul.* | DMC | Bark | Alcoholic Infusion | Aguiar & Barros, 2012 |
| *Caesalpinia bracteosa Tul.* | DDS | Leaves | Infusion | Chaves & Barros, 2012 |
| *Caesalpinia bracteosa Tul.* | IPD, DGS, DDS, DRS, OTHER | Bark, Leaves |  | Oliveira et al., 2010 |
| *Caesalpinia echinata Lam.* |  |  |  | Fransclin, B.P. (1978) UNICAMP |
| *Caesalpinia echinata Lam.* | OTHER |  |  | Agra et al, 2007 |
| *Caesalpinia echinata Lam.* | OTHER |  |  | Agra et al, 2008 |
| *Caesalpinia echinata Lam.* | ENM, DDS, DFS | Stem |  | Albuquerque et al, 2007 |
| *Caesalpinia echinata Lam.* |  |  |  | Brandão et al, 2012 |
| *Caesalpinia ferrea C.Mart.* | OTHER |  |  | Oliveira, F.R. de S. de (1994) UFERSA |
| *Caesalpinia ferrea C.Mart.* |  |  |  | Morais, H.R.M.D. (2003) UFERSA |
| *Caesalpinia ferrea C.Mart.* |  |  |  | Oliveira, O.F. de (1973) UFERSA |
| *Caesalpinia ferrea C.Mart.* |  |  |  | Oliveira, O.F. de (1973) UFERSA |
| *Caesalpinia ferrea C.Mart.* |  |  |  | Oliveira, O.F. de (1973) UFERSA |
| *Caesalpinia ferrea C.Mart.* |  |  |  | Oliveira, O.F. de (1973) UFERSA |
| *Caesalpinia ferrea C.Mart.* |  |  |  | Oliveira, O.F. de (1973) UFERSA |
| *Caesalpinia ferrea C.Mart.* | DFS, DDS |  |  | Constãncia Ayres UFPE |
| *Caesalpinia ferrea C.Mart.* | DDS, ENM | Bark | Alcoholic Infusion | Agra et al, 2007 |
| *Caesalpinia ferrea C.Mart.* | DDS, ENM | Bark | Alcoholic Infusion | Agra et al, 2008 |
| *Caesalpinia ferrea C.Mart.* | DNS, DRS, DGS | Bark | Alcoholic Infusion | Aguiar & Barros, 2012 |
| *Caesalpinia ferrea C.Mart.* |  |  |  | Albuquerque & Oliveira, 2007 |
| *Caesalpinia ferrea C.Mart.* | DRS, ENM, DNS | Bark, Leaves, Seeds, Flowers |  | Albuquerque et al, 2007 |
| *Caesalpinia ferrea C.Mart.* |  | Bark |  | Alencar et al., 2010 |
| *Caesalpinia ferrea C.Mart.* | DRS | Bark, Flower, Seed | Decoction | Almeida et al., 2006 |
| *Caesalpinia ferrea C.Mart.* |  |  |  | Amorozo, 2002 |
| *Caesalpinia ferrea C.Mart.* | DRS, ENM, DFS, OTHER | Bark, Fruits, Seeds | Decoction, Alcoholic Infusion | Chaves & Barros, 2012 |
| *Caesalpinia ferrea C.Mart.* | DDS, DFS, DCS, OTHER | Fruit | Syrup, Alcoholic Infusion, Maceration | Coelho-Ferreira, 2009 |
| *Caesalpinia ferrea C.Mart.* | DDS, DRS, DSS, ENM | Seed |  | Conceição et al, 2011 |
| *Caesalpinia ferrea C.Mart.* | IPD | Leaves | Tea | David & Pasa, 2013 |
| *Caesalpinia ferrea C.Mart.* | OTHER | Bark, Fruit |  | Gazzaneo et al., 2005 |
| *Caesalpinia ferrea C.Mart.* |  |  |  | Guarim Neto & Morais, 2003 |
| *Caesalpinia ferrea C.Mart.* | OTHER | Leaves | Decoction | Jesus et al., 2009 |
| *Caesalpinia ferrea C.Mart.* | DRS |  |  | Lozano et al., 2014 |
| *Caesalpinia ferrea C.Mart.* | IPD | Leaves | Bath | Macedo et al, 2011 |
| *Caesalpinia ferrea C.Mart.* | OTHER | Seeds | Alcoholic Infusion | Morais et al, 2005 |
| *Caesalpinia ferrea C.Mart.* | ENM, DDS, DCS, DRS | Bark, Leaves, Flowers |  | Oliveira et al., 2010 |
| *Caesalpinia ferrea C.Mart.* | ENM, OTHER |  |  | Pilla et al, 2006 |
| *Caesalpinia ferrea C.Mart.* | DMC | Bark, Fruit | Tea, Wine | Pinto et al., 2013 |
| *Caesalpinia ferrea C.Mart.* |  |  |  | Roque et al., 2010 |
| *Caesalpinia ferrea C.Mart.* | ENM, OTHER | Fruit Bark | Infusion Or Maceration | Santos et al, 2008 |
| *Caesalpinia ferrea C.Mart.* |  |  |  | Silva & Albuquerque, 2005 |
| *Caesalpinia ferrea C.Mart.* | DMC, OTHER | Fruit | Alcoholic Infusion | Silva & Freire, 2010 |
| *Caesalpinia ferrea C.Mart.* | DMC, DRS | Fruit | Tincture | Siviero et al., 2012 |
| *Caesalpinia ferrea C.Mart.* | ENM | Bark | Tea | Trojan-Rodrigues et al, 2012 |
| *Caesalpinia leiostachya (Benth.) Ducke* |  |  |  | Marques, A.S.C. (2009) UFERSA |
| *Caesalpinia leiostachya (Benth.) Ducke* |  |  |  | G. Teixeira (1965) UFRPE |
| *Caesalpinia leiostachya (Benth.) Ducke* |  |  |  | G. Teixeira (1964) UFRPE |
| *Caesalpinia microphylla G.Don* | DDS, DNS | Bark | Decoction With Water | Agra et al, 2007 |
| *Caesalpinia microphylla G.Don* | DDS, DNS | Bark | Decoction With Water | Agra et al, 2008 |
| *Caesalpinia microphylla G.Don* | DMC, OTHER | Bark, Leaves |  | Albuquerque et al, 2007 |
| *Caesalpinia microphylla Martius* | DDS | Leaves | Tea | Castro et al., 2011 |
| *Caesalpinia microphylla G.Don* |  |  |  | Silva & Albuquerque, 2005 |
| *Caesalpinia pulcherrima (L.) Sw.* | DDS | Leaves | Decoction With Water | Agra et al, 2008 |
| *Caesalpinia pulcherrima (L.) Sw.* | DDS | Leaves | Decoction With Water | Agra et al, 2008 |
| *Caesalpinia pulcherrima (L.) Sw.* | DDS | Leaves, Flowers |  | Albuquerque et al, 2007 |
| *Caesalpinia pulcherrima (L.) Sw.* | DGS | Bark, Leaf | Decoction | Almeida et al., 2006 |
| *Caesalpinia pulcherrima (L.) Sw.* |  |  |  | Amorozo, 2002 |
| *Caesalpinia pulcherrima (L.) Sw.* | DDS | Leaves |  | Breitbach et al., 2013 |
| *Caesalpinia pyramidalis Tul.* | DDS | Bark |  | Ferreira, M. do S.G. (1980) INPA |
| *Caesalpinia pyramidalis Tul.* |  |  |  | Susiany; Camila; Camila (2010) IPA |
| *Caesalpinia pyramidalis Tul.* |  |  |  | Mattos-Silva, L.A. (2004) JBRJ |
| *Caesalpinia pyramidalis Tul.* |  |  |  | S. Ginzbarg (1988) MBML |
| *Caesalpinia pyramidalis Tul.* | DDS, DRS |  |  | Londoño, P.A. (2010) UFPE |
| *Caesalpinia pyramidalis Tul.* |  |  |  | G. Teixeira (1965) UFRPE |
| *Caesalpinia pyramidalis Tul.* | DDS, DRS | Bark | Alcoholic Infusion, Syrup, Decoction With Water | Agra et al, 2007 |
| *Caesalpinia pyramidalis Tul.* | OTHER | Bark | Alcoholic Infusion | Agra et al, 2007 |
| *Caesalpinia pyramidalis Tul.* | DDS, DRS | Bark | Alcoholic Infusion, Syrup, Decoction With Water | Agra et al, 2008 |
| *Caesalpinia pyramidalis Tul.* |  |  |  | Albuquerque & Oliveira, 2007 |
| *Caesalpinia pyramidalis Tul.* | DRS, DDS, ENM, OTHER | Stem, Bark, Leaves, Root |  | Albuquerque et al, 2007 |
| *Caesalpinia pyramidalis Tul.* |  | Bark |  | Alencar et al., 2010 |
| *Caesalpinia pyramidalis Tul.* | DDS | Leaves | Decoction | Almeida et al., 2006 |
| *Caesalpinia pyramidalis Tul.* | DDS, IPD | Leaves | Tea | Barbosa da Silva et al. 2012 |
| *Caesalpinia pyramidalis Tul.* | DRS, DDS, ENM | Flowers, Bark | Drink Or Wash | Cartaxo et al, 2010 |
| *Caesalpinia pyramidalis Tul.* | DRS | Bark | Syrup | de Albuquerque, 2006 |
| *Caesalpinia pyramidalis Tul.* | DRS | Flower |  | Freitas et al, 2012 |
| *Caesalpinia pyramidalis Tul.* |  |  |  | Lucena et al., 2007 |
| *Caesalpinia pyramidalis Tul.* |  |  |  | Lucena et al., 2008 |
| *Caesalpinia pyramidalis Tul.* | DDS, ENM, OTHER | Bark |  | Oliveira et al., 2010 |
| *Caesalpinia pyramidalis Tul.* |  |  |  | Roque et al., 2010 |
| *Caesalpinia pyramidalis Tul.* |  |  |  | Silva & Albuquerque, 2005 |
| *Caesalpinia pyramidalis Tul.* | DMC, DRS, DDS, OTHER | Bark, Fruit | Infusion | Silva & Freire, 2010 |
| *Caesalpinia pyramidalis Tul.* |  |  |  | Silva e Andrade, 2005 |
| *Cajanus cajan (L.) Millsp.* |  |  |  | Stavinski, M.N.R. (1982) INPA |
| *Cajanus cajan (L.) Millsp.* |  |  |  | Silva, I.M. (2005) JBRJ |
| *Cajanus cajan (L.) Millsp.* |  |  |  | Soares, A.; W.Maschio; W.Maschio (1999) UEM |
| *Cajanus cajan (L.) Millsp.* |  |  |  | S.A. Cunha (2008) UFMS |
| *Cajanus cajan (L.) Millsp.* |  |  |  | R. Shardong (1998) UFMS |
| *Cajanus cajan (L.) Millsp.* |  |  |  | M.A. do Prado (2002) UFMS |
| *Cajanus cajan (L.) Millsp.* |  |  |  | Silva, V.A. (2002) UFPE |
| *Cajanus cajan (L.) Millsp.* | DMC | Leaves | Decoction With Water | Agra et al, 2007 |
| *Cajanus cajan (L.) Millsp.* | DMC | Leaves | Decoction With Water | Agra et al, 2008 |
| *Cajanus cajan (L.) Millsp.* |  |  |  | Albuquerque & Oliveira, 2007 |
| *Cajanus cajan (L.) Millsp.* |  |  |  | Amorozo, 2002 |
| *Cajanus cajan (L.) Millsp.* |  |  |  | Amorozo, 2004 |
| *Cajanus cajan (L.) Millsp.* | DRS | Flowers, Leaves | Syrup, Infusion | Barbosa da Silva et al. 2012 |
| *Cajanus cajan (L.) Millsp.* | DDS | Leaves | Tea | Begossi et al, 1993 |
| *Cajanus cajan (L.) Millsp.* | DDS | Leaves | Tea | Borba & Macedo, 2006 |
| *Cajanus cajan (L.) Millsp.* |  |  |  | Brito & Senna-Valle, 2012 |
| *Cajanus cajan (L.) Millsp.* | DCS, OTHER | Leaves | Bath, Alcoholic Infusion | Coelho-Ferreira, 2009 |
| *Cajanus cajan (L.) Millsp.* |  |  |  | Cunha & Bortolotto, 2011 |
| *Cajanus cajan (L.) Millsp.* |  |  |  | Eichemberg et al., 2009 |
| *Cajanus cajan (L.) Millsp.* | IPD | Leaves | Tea | Feijo et al., 2013 |
| *Cajanus cajan (L.) Millsp.* |  |  |  | Gandolfo & Hanazaki, 2011 |
| *Cajanus cajan (L.) Millsp.* | DRS | Leaves | Infusion | Garcia et al., 2010 |
| *Cajanus cajan (L.) Millsp.* | DRS |  |  | Giraldi & Hanazaki, 2010 |
| *Cajanus cajan (L.) Millsp.* | DRS |  |  | Lozano et al., 2014 |
| *Cajanus cajan (L.) Millsp.* | OTHER | Seeds | Plaster | Morais et al, 2005 |
| *Cajanus cajan (L.) Millsp.* | DFS |  |  | Pilla et al, 2006 |
| *Cajanus cajan (L.) Millsp.* |  |  |  | Silva e Andrade, 2005 |
| *Cajanus canjan (L.) Millsp.* |  |  |  | Staviski, M.N.R. (1982) JBRJ |
| *Calliandra aeschynomenoides Benth.* |  |  |  | Silva & Albuquerque, 2005 |
| *Calliandra depauperata Benth.* | ENM, DRS | Leaves, Bark | Decoction | Agra et al, 2007 |
| *Calliandra depauperata Benth.* | ENM, DRS | Leaves, Bark | Decoction | Agra et al, 2008 |
| *Calliandra dysantha Benth.* | DFS | Root, Flower | Decoction | Vila Verde et al, 2003 |
| *Calliandra parviflora Benth.* |  |  |  | Harley, R.M. (1968) JBRJ |
| *Calliandra umbellifera Benth.* |  |  |  | Emperaire, L. (1985) JBRJ |
| *Campsiandra comosa var. laurifolia (Benth.) Cowan* |  |  |  | Roa T., A. (1977) INPA |
| *Campsiandra implexicaulis Stergios* |  |  |  | Martinelli, G. (1980) JBRJ |
| *Camptosema coriaceum Benth.* | OTHER | Root |  | Brandao et al, 2012 |
| *Camptosema coriaceum Benth.* | OTHER | Roots |  | Brandão et al, 2012 |
| *Camptosema ellipticum (Desv.) Burkart* |  |  |  | D.C. Ibrahim (2006) UFMS |
| *Cassia ferruginea (Schrad.) DC.* |  |  |  | Fernandes et al., 2014 |
| *Cassia grandis L.f.* | OTHER | Bark |  | Brandao et al, 2012 |
| *Cassia grandis L.f.* |  |  |  | Brandão et al, 2012 |
| *Cenostigma gardnerianum Tul.* |  |  |  | F. C. S. Oliveira (2007) JBRJ |
| *Cenostigma gardnerianum Tul.* | DMC | Bark, Resin | Decoction With Water | Agra et al, 2007 |
| *Cenostigma gardnerianum Tul.* | DMC | Bark, Resin | Decoction With Water | Agra et al, 2008 |
| *Centrosema bracteosum Benth.* | DDS | Root |  | Alvarenga, D. (1999) JBRJ |
| *Centrosema bracteosum Benth.* |  |  |  | R.C. Mendonca (1991) JBRJ |
| *Centrosema bracteosum Benth.* | ENM | Bark | Tea | Pinto et al., 2013 |
| *Centrosema bracteosum Benth.* | DDS | Root | Decoction | Vila Verde et al, 2003 |
| *Centrosema brasilianum (L.) Benth.* |  |  |  | G.B. Freitas (2006) JBRJ |
| *Centrosema brasilianum (L.) Benth.* |  |  |  | Freitas, G.B. (2006) UFPB |
| *Centrosema brasilianum (L.) Benth.* | IPD | Whole Plant | Decoction | Agra et al, 2007 |
| *Centrosema brasilianum (L.) Benth.* | IPD | Whole Plant | Decoction With Water | Agra et al, 2007 |
| *Centrosema brasilianum (L.) Benth.* | IPD | Whole Plant | Decoction With Water | Agra et al, 2008 |
| *Centrosema plumieri (Pers.) Benth.* |  |  |  | Bahri, S. (1988) INPA |
| *Centrosema pubescens Benth.* |  |  |  | Guarim Neto & Morais, 2003 |
| *Centrosema virginianum (L.) Benth.* |  |  |  | Staviski, M.N.R.; Maciel, M.A.; Maciel, M.A. (1983) IPA |
| *Chamaecrista desvauxii (Collad.) Killip* |  |  |  | Sobrinho, J.S. (1965) IPA |
| *Chamaecrista desvauxii (Collad.) Killip* |  |  |  | Amorozo, 2002 |
| *Chamaecrista desvauxii (Collad.) Killip* |  |  |  | Amorozo, 2004 |
| *Chamaecrista desvauxii (Collad.) Killip* |  |  |  | Guarim Neto & Morais, 2003 |
| *Chamaecrista desvauxii (Collad.) Killip* | IPD | Leaves | Bath | Macedo et al, 2011 |
| *Chamaecrista desvauxii var. latistipula (Benth.) G.P.Lewis* | DGS | Whole Plant | Decoction | Hirschmann & Arias, 1990 |
| *Chamaecrista dyphylla* | DRS |  |  | Castro et al., 2011 |
| *Chamaecrista fagonioides (Vogel) H.S. Irwin & Barneby* |  |  |  | Correa, D.A. (1949) JBRJ |
| *Chamaecrista flexuosa (L.) Greene* | DGS | Root | Decoction With Water | Agra et al, 2008 |
| *Chamaecrista flexuosa (L.) Greene* | DGS | Root | Decoction With Water | Agra et al, 2008 |
| *Chamaecrista flexuosa (L.) Greene* | DGS | Roots |  | Albuquerque et al, 2007 |
| *Chamaecrista flexuosa (L.) Greene* | DDS | Leaves, Flower | Decoction | Almeida et al., 2006 |
| *Chamaecrista neesiana (Benth.) H.S.Irwin & Barneby* | DDS | Leaves | Decoction | Hirschmann & Arias, 1990 |
| *Chamaecrista nictitans (L.) Moench* |  |  |  | D.C. Ibrahim (2006) UFMS |
| *Chamaecrista papillata H.S.Irwin & Barneby* | DDS | Leaves | Decoction | Hirschmann & Arias, 1990 |
| *Chamaecrista pilosa (L.) Greene* | DSS |  |  | Espinola, M.C. (1942) UFPB |
| *Chamaecrista repens (Vogel) H.S.Irwin & Barneby* |  |  |  | David et al., 2007 |
| *Chloroleucon dumosum (Benth.) G.P.Lewis* | ENM, OTHER | Bark | Decoction | Agra et al, 2007 |
| *Chloroleucon dumosum (Benth.) G.P.Lewis* | ENM, OTHER | Bark | Decoction | Agra et al, 2008 |
| *Chloroleucon extortum Barneby & J.W.Grimes* |  | Bark |  | Alencar et al., 2010 |
| *Chloroleucon foliolosum (Benth.) G.P.Lewis* |  |  |  | G.T. Soldati (2008) UFRPE |
| *Chloroleucon foliolosum (Benth.) G.P.Lewis* | ENM, OTHER | Bark | Decoction | Agra et al, 2007 |
| *Chloroleucon foliolosum (Benth.) G.P.Lewis* | ENM, OTHER | Bark | Decoction | Agra et al, 2008 |
| *Chloroleucon foliolosum (Benth.) G.P.Lewis* | OTHER | Bark | Infusion | Silva & Freire, 2010 |
| *Chloroleucon mangense (Jacq.) Britton & Rose* |  |  |  | Silva & Albuquerque, 2005 |
| *Cleobulia multiflora Benth.* |  |  |  | Brandão et al, 2012 |
| *Clitoria guianensis (Aubl.) Benth.* |  |  |  | Guarim Neto & Morais, 2003 |
| *Clitoria guianensis (Aubl.) Benth.* | DGS, OTHER | Root | Infusion | Hirschmann & Arias, 1990 |
| *Clitoria guianensis (Aubl.) Benth.* | DNS |  |  | Souza & Felfilli, 2006 |
| *Clitoria guianensis (Aubl.) Benth.* | DNS | Root | Decoction Or Alcoholic Infusion | Vila Verde et al, 2003 |
| *Clitoria simplicifolia (Kunth) Benth.* | OTHER |  |  | Rodrigues, 2007 |
| *Clitoria ternatea L.* | DDS, IPD | Roots, Seeds | Infusion With Water | Agra et al, 2007 |
| *Clitoria ternatea L.* | DDS, IPD | Roots, Seeds | Infusion With Water | Agra et al, 2008 |
| *Copaifera coriacea Mart.* |  |  |  | J.S. Sobrinho (1966) UFRPE |
| *Copaifera duckei Dwyer* |  |  |  | J.S. Sobrinho (1966) UFRPE |
| *Copaifera elliptica Mart.* |  |  |  | Guarim Neto & Morais, 2003 |
| *Copaifera guyanensis Desf.* | DGS | Resin |  | Breitbach et al., 2013 |
| *Copaifera guyanensis Desf.* | OTHER |  |  | Rodrigues, 2007 |
| *Copaifera langsdorffii Desf.* |  |  |  | Teixeira, G. (1965) IPA |
| *Copaifera langsdorffii Desf.* | DRS | Bark | Tea | R. Barros (2007) JBRJ |
| *Copaifera langsdorffii Desf.* |  |  |  | Andrade A.C. da S. (2002) JBRJ |
| *Copaifera langsdorffii Desf.* |  |  |  | Andrade A.C. da S. (2002) JBRJ |
| *Copaifera langsdorffii Desf.* |  |  |  | S A Mori (1978) JBRJ |
| *Copaifera langsdorffii Desf.* |  |  |  | F. A. Sobrinho (2006) JBRJ |
| *Copaifera langsdorffii Desf.* |  |  |  | M.C. de Souza (2006) UFMS |
| *Copaifera langsdorffii Desf.* |  |  |  | A.L.B. Sartori (2002) UFMS |
| *Copaifera langsdorffii Desf.* | OTHER | Bark | Decoction With Water | Agra et al, 2007 |
| *Copaifera langsdorffii Desf.* | OTHER | Bark | Decoction With Water | Agra et al, 2008 |
| *Copaifera langsdorffii Desf.* | OTHER | Bark, Resin |  | Albuquerque et al, 2007 |
| *Copaifera langsdorffii Desf.* | DRS | Bark | Infusion | Bueno et al., 2005 |
| *Copaifera langsdorffii Desf.* | DDS, DRS, DMC, OTHER | Leaves, Seed |  | Conceição et al, 2011 |
| *Copaifera langsdorffii Desf.* |  |  |  | Cunha & Bortolotto, 2011 |
| *Copaifera langsdorffii Desf.* |  |  |  | Fernandes et al., 2014 |
| *Copaifera langsdorffii Desf.* |  |  |  | Guarim Neto & Morais, 2003 |
| *Copaifera langsdorffii Desf.* | OTHER , DDS | Bark | Tea, Bath | Guarim-Neto, 2006 |
| *Copaifera langsdorffii Desf.* | DMC |  |  | Lozano et al., 2014 |
| *Copaifera langsdorffii Desf.* | DMC | Bark | Alcoholic Infusion | Monteles & Pinheiro, 2007 |
| *Copaifera langsdorffii Desf.* | OTHER | Resin |  | Pasa et al, 2011 |
| *Copaifera langsdorffii Desf.* | DDS | Resin | Oil | Pinto et al., 2013 |
| *Copaifera langsdorffii Desf.* | DGS, DRS | Bark, Oil | Decoction Or Oil | Santos et al, 2008 |
| *Copaifera langsdorffii Desf.* | NEO, DEA, DDS, OTHER |  |  | Souza & Felfilli, 2006 |
| *Copaifera lucens Dwyer* | DCS, DEA, DRS, OTHER | Oil | Oil | Barbosa da Silva et al. 2012 |
| *Copaifera martii Hayne* |  |  |  | Nave, A.G.; Carneiro, P.H.M.; Dario, F.R.; Lopes, R.F. (1997) UNICAMP |
| *Copaifera martii Hayne* |  |  |  | Nave, A.G.; Carneiro, P.H.M.; Dario, F.R.; Lopes, R.F. (1997) UNICAMP |
| *Copaifera martii Hayne* | DGS | Resin |  | Breitbach et al., 2013 |
| *Copaifera martii Hayne* | DRS, NEO, DDS | Oil | With Honey | Chaves & Barros, 2012 |
| *Copaifera martii Hayne* | DFS, OTHER | Bark | Tea, Alcoholic Infusion | Coelho-Ferreira, 2009 |
| *Copaifera martii Hayne* |  |  |  | Guarim Neto & Morais, 2003 |
| *Copaifera multijuga Hayne* | NEO, OTHER | Fruit Oil |  | Agra et al, 2007 |
| *Copaifera multijuga Hayne* | NEO, OTHER | Fruit Oil |  | Agra et al, 2008 |
| *Copaifera reticulata Ducke* | NEO, OTHER | Whole Plant |  | Agra et al, 2007 |
| *Copaifera reticulata Ducke* | NEO, OTHER | Whole Plant |  | Agra et al, 2008 |
| *Copaifera reticulata Ducke* |  |  |  | Shanley & Rosa, 2004 |
| *Copaifera reticulata Ducke* | DRS, OTHER | Resin |  | Shanley and Luz, 2003 |
| *Copaifera trapezifolia Hayne* |  |  |  | F. A. Sobrinho (2005) JBRJ |
| *Copaifera trapezifolia Hayne* |  |  |  | F.A. Sobrinho (2006) JBRJ |
| *Crotalaria incana L.* | DFS |  |  | Lozano et al., 2014 |
| *Crotalaria maypurensis Kunth* | OTHER |  |  | Rodrigues, 2007 |
| *Crotalaria micans Link* |  |  |  | Guarim Neto & Morais, 2003 |
| *Crotalaria micans Link* |  |  |  | Pinto et al, 2006 |
| *Crotalaria pallida Aiton* |  |  |  | S.A. Cunha (2009) UFMS |
| *Crotalaria retusa L.* |  |  |  | S.A. Cunha (2009) UFMS |
| *Crotalaria spectabilis Roth* | IPD | Leaves | Tea | Aguiar & Barros, 2012 |
| *Crudia amazonica Benth.* | IPD |  |  | Prance, G.T. (1975) INPA |
| *Cyclolobium brasiliense Benth.* |  |  |  | Pereira, B.A.S. (1991) JBRJ |
| *Cynometra spruceana Benth.* | DRS | Resin |  | Breitbach et al., 2013 |
| *Dalbergia catingicola Harms* |  |  |  | Silva & Albuquerque, 2005 |
| *Dalbergia cearensis Ducke* |  |  |  | Silva & Albuquerque, 2005 |
| *Dalbergia ecastaphyllum (L.) Taub.* | DFS, ENM, PCP | Bark | Alcoholic Infusion | Coelho-Ferreira, 2009 |
| *Dalbergia monetaria L.f.* |  |  |  | Carvalho, A.M.V. de (1986) JBRJ |
| *Dalbergia monetaria L.f.* | DFS, ENM, PCP | Bark | Alcoholic Infusion | Coelho-Ferreira, 2009 |
| *Dalbergia subcymosa Ducke* | DFS | Bark |  | Shanley and Luz, 2003 |
| *Derris floribunda (Miq.) Benth.* | OTHER | Bark | Juice In Bath | Coelho-Ferreira, 2009 |
| *Desmodium adscendens (Sw.) DC.* |  |  |  | Leitao, F. (2005) JBRJ |
| *Desmodium adscendens (Sw.) DC.* |  |  |  | Leitao, F. (2006) JBRJ |
| *Desmodium adscendens (Sw.) DC.* |  |  |  | Silva, I.M. (2005) JBRJ |
| *Desmodium adscendens (Sw.) DC.* |  |  |  | Silva, M.A. da (1995) JBRJ |
| *Desmodium adscendens (Sw.) DC.* | DGS |  |  | Aline Redondo Martins (2002) UNESPSJRP |
| *Desmodium adscendens (Sw.) DC.* |  |  |  | Hanazaki, N.; Peroni, N.; Verissimo, M.M. (1998) UNICAMP |
| *Desmodium adscendens (Sw.) DC.* |  | Leaves |  | Azevedo & Kruel, 2007 |
| *Desmodium adscendens (Sw.) DC.* |  |  |  | Brito & Senna-Valle, 2012 |
| *Desmodium adscendens (Sw.) DC.* | DGS |  |  | Giraldi & Hanazaki, 2010 |
| *Desmodium adscendens (Sw.) DC.* |  |  |  | Guarim Neto & Morais, 2003 |
| *Desmodium adscendens (Sw.) DC.* |  |  |  | Pinto et al, 2006 |
| *Desmodium adscendens (Sw.) DC.* |  |  |  | Silva & Proença, 2008 |
| *Desmodium adscendens (Sw.) DC.* | DGS | Leaves | Tea | Siviero et al., 2012 |
| *Desmodium barbatum (L.) Benth.* |  |  |  | Patzlaff,R. (2000) JBRJ |
| *Desmodium barbatum (L.) Benth.* |  |  |  | Figueiredo et al., 1993 |
| *Desmodium incanum DC.* |  |  |  | R. Schardong (1998) UFMS |
| *Desmodium incanum DC.* |  |  |  | M.A. do Prado (2002) UFMS |
| *Desmodium incanum DC.* | DBI, DGS |  |  | Aita et al., 2009 |
| *Desmodium incanum DC.* |  |  |  | Amorozo, 2002 |
| *Desmodium incanum DC.* | OTHER |  |  | Conde et al., 2014 |
| *Desmodium incanum DC.* |  |  |  | Guarim Neto & Morais, 2003 |
| *Desmodium incanum DC.* |  |  |  | Silva et al., 2013 |
| *Desmodium triflorum (L.) DC.* | OTHER | Leaves | Bath | Medeiros et al, 2004 |
| *Dialium guianense (Aubl.) Sandwith* |  |  |  | S. Tavares (1962) UFRPE |
| *Dimorphandra gardneriana Tul.* |  |  |  | Assumpcao, S.P. (1977) INPA |
| *Dimorphandra gardneriana Tul.* | DCS, ENM | Fruits | Decoction With Water | Agra et al, 2007 |
| *Dimorphandra gardneriana Tul.* | DCS, ENM | Fruits | Decoction With Water | Agra et al, 2008 |
| *Dimorphandra gardneriana Tul.* | DMC |  |  | Lozano et al., 2014 |
| *Dimorphandra gardneriana Tul.* | DRS | Flowers |  | Oliveira et al., 2010 |
| *Dimorphandra mollis Benth.* |  |  |  | S.A. Cunha (2009) UFMS |
| *Dimorphandra mollis Benth.* |  |  |  | A. Oliveira (1990) UFMS |
| *Dimorphandra mollis Benth.* |  |  |  | Rita (1989) UFMS |
| *Dimorphandra mollis Benth.* | DMC | Bark | Infusion | Bueno et al., 2005 |
| *Dimorphandra mollis Benth.* |  |  |  | Cunha & Bortolotto, 2011 |
| *Dimorphandra mollis Benth.* |  |  |  | Guarim Neto & Morais, 2003 |
| *Dimorphandra mollis Benth.* |  |  |  | Guarim Neto & Pasa, 2009 |
| *Dimorphandra mollis Benth.* | DFS, OTHER |  |  | Souza & Felfilli, 2006 |
| *Dimorphandra mollis Benth.* | DFS, OTHER | Bark | Bath, Tea, Alcoholic Infusion | Souza, 2007 |
| *Dimorphandra mollis Benth.* | OTHER | Fruit | Infusion | Vila Verde et al, 2003 |
| *Dioclea grandiflora Benth.* |  |  |  | Agra, M.F.; Mugeut, B.R.; Mugeut, B.R. (1986) IPA |
| *Dioclea grandiflora Benth.* |  |  |  | S.A. Cunha (2009) UFMS |
| *Dioclea grandiflora Benth.* | DGS | Roots | Decoction | Agra et al, 2007 |
| *Dioclea grandiflora Benth.* | DGS | Roots | Decoction With Water | Agra et al, 2007 |
| *Dioclea grandiflora Benth.* | DGS | Roots | Decoction With Water | Agra et al, 2008 |
| *Dioclea grandiflora Benth.* | DNS, DRS | Leaves, Flower, Fruit | Macerated | Almeida et al., 2006 |
| *Dioclea grandiflora Benth.* |  |  |  | Cunha & Bortolotto, 2011 |
| *Dioclea grandiflora Benth.* | DMC |  |  | Lozano et al., 2014 |
| *Dioclea grandiflora Benth.* |  |  |  | Roque et al., 2010 |
| *Dioclea violacea Benth.* |  |  |  | Agra, M.F. (1987) UFPB |
| *Dioclea violacea Benth.* | DCS |  |  | Aline Redondo Martins (2002) UNESPSJRP |
| *Dioclea violacea Benth.* | DCS | Seeds | Decoction With Water | Agra et al, 2008 |
| *Dioclea violacea Benth.* | DCS | Seeds | Decoction With Water | Agra et al, 2008 |
| *Dioclea violacea Benth.* | DBI, DMC | Seeds | Tea | Bratti et al., 2013 |
| *Dioclea violacea Benth.* | DRS | Latex | Juice | Franco & Barros, 2006 |
| *Dioclea violacea Benth.* | DRS | Latex |  | Oliveira et al., 2010 |
| *Dioclea virgata (Rich.) Amshoff* | OTHER | Leaves | Decoction With Water | Agra et al, 2007 |
| *Dioclea virgata (Rich.) Amshoff* | OTHER | Leaves | Decoction With Water | Agra et al, 2008 |
| *Diplotropis ferruginea Benth.* | DFS, OTHER | Bark | Bath | Agra et al, 2007 |
| *Diplotropis ferruginea Benth.* | DFS, OTHER | Bark | Bath | Agra et al, 2008 |
| *Dipteryx alata Vogel* |  |  |  | S.A. Cunha (2009) UFMS |
| *Dipteryx alata Vogel* |  |  |  | G. Camargo (2004) UFMS |
| *Dipteryx alata Vogel* |  |  |  | Amorozo, 2002 |
| *Dipteryx alata Vogel* |  |  |  | Amorozo, 2004 |
| *Dipteryx alata Vogel* |  |  |  | Cunha & Bortolotto, 2011 |
| *Dipteryx alata Vogel* |  |  |  | Guarim Neto & Morais, 2003 |
| *Dipteryx alata Vogel* | DDS, OTHER | Leaves, Bark | Tea | Guarim-Neto, 2006 |
| *Dipteryx alata Vogel* | DCS | Bark | Infusion | Silva et al., 2010 |
| *Dipteryx alata* | DMC |  |  | Souza & Felfilli, 2006 |
| *Dipteryx alata Vogel* | DMC | Seed | Edible | Vila Verde et al, 2003 |
| *Dipteryx lacunifera Ducke* |  |  |  | Ribeiro, F.S. (2009) IPA |
| *Dipteryx lacunifera Ducke* |  |  |  | Ribeiro, F.S. (2009) IPA |
| *Dipteryx lacunifera Ducke* |  |  |  | Ribeiro, F.S. (2009) IPA |
| *Dipteryx lacunifera Ducke* |  |  |  | Ribeiro, F.S. (2009) IPA |
| *Dipteryx lacunifera Ducke* |  |  |  | Ribeiro, F.S. (2009) IPA |
| *Dipteryx lacunifera Ducke* |  |  |  | Ribeiro, F.S. (2009) IPA |
| *Dipteryx lacunifera Ducke* |  |  |  | Ribeiro, F.S. (2010) IPA |
| *Dipteryx lacunifera Ducke* |  |  |  | Ribeiro, F.S. (2009) IPA |
| *Dipteryx lacunifera Ducke* |  |  |  | Ribeiro, F.S. (2009) IPA |
| *Dipteryx lacunifera Ducke* |  |  |  | Ribeiro, F.S. (2009) IPA |
| *Dipteryx lacunifera Ducke* |  |  |  | Ribeiro, F.S. (2009) IPA |
| *Dipteryx lacunifera Ducke* |  |  |  | Ribeiro, F.S. (2009) IPA |
| *Dipteryx lacunifera Ducke* |  |  |  | Ribeiro, F.S. (2009) IPA |
| *Dipteryx lacunifera Ducke* |  |  |  | Ribeiro, F.S. (2009) IPA |
| *Dipteryx lacunifera Ducke* |  |  |  | Ribeiro, F.S. (2009) IPA |
| *Dipteryx lacunifera Ducke* |  |  |  | Ribeiro, F.S. (2009) IPA |
| *Dipteryx lacunifera Ducke* |  |  |  | Ribeiro, F.S. (2009) IPA |
| *Dipteryx lacunifera Ducke* |  |  |  | Ribeiro, F.S. (2009) IPA |
| *Dipteryx lacunifera Ducke* |  |  |  | Ribeiro, F.S. (2009) IPA |
| *Dipteryx lacunifera Ducke* |  |  |  | Ribeiro, F.S. (2009) IPA |
| *Dipteryx lacunifera Ducke* |  |  |  | Ribeiro, F.S. de (2009) JBRJ |
| *Dipteryx lacunifera Ducke* |  |  |  | Ribeiro, F.S. de (2009) JBRJ |
| *Dipteryx lacunifera Ducke* |  |  |  | Ribeiro, F.S. de (2009) JBRJ |
| *Dipteryx lacunifera Ducke* |  |  |  | Ribeiro, F.S. de (2009) JBRJ |
| *Dipteryx lacunifera Ducke* |  |  |  | Ribeiro, F.S. de (2009) JBRJ |
| *Dipteryx lacunifera Ducke* |  |  |  | Ribeiro, F.S. de (2009) JBRJ |
| *Dipteryx lacunifera Ducke* |  |  |  | Ribeiro, F.S. de (2009) JBRJ |
| *Dipteryx lacunifera Ducke* |  |  |  | Ribeiro, F.S. de (2009) JBRJ |
| *Dipteryx lacunifera Ducke* |  |  |  | Ribeiro, F.S. de (2009) JBRJ |
| *Dipteryx lacunifera Ducke* |  |  |  | Ribeiro, F.S. de (2009) JBRJ |
| *Dipteryx lacunifera Ducke* |  |  |  | Ribeiro, F.S. de (2009) JBRJ |
| *Dipteryx lacunifera Ducke* |  |  |  | Ribeiro, F.S. de (2009) JBRJ |
| *Dipteryx lacunifera Ducke* |  |  |  | Ribeiro, F.S. de (2009) JBRJ |
| *Dipteryx lacunifera Ducke* |  |  |  | Ribeiro, F.S. de (2009) JBRJ |
| *Dipteryx lacunifera Ducke* |  |  |  | Ribeiro, F.S. de (2009) JBRJ |
| *Dipteryx lacunifera Ducke* |  |  |  | Ribeiro, F.S. de (2009) JBRJ |
| *Dipteryx lacunifera Ducke* |  |  |  | Ribeiro, F.S. de (2009) JBRJ |
| *Dipteryx lacunifera Ducke* |  |  |  | Ribeiro, F.S. de (2009) JBRJ |
| *Dipteryx lacunifera Ducke* |  |  |  | Ribeiro, F.S. de (2009) JBRJ |
| *Dipteryx lacunifera Ducke* |  |  |  | Ribeiro, F.S. de (2009) JBRJ |
| *Dipteryx odorata (Aubl.) Willd.* | DDS, DNS, DCS | Seeds |  | Breitbach et al., 2013 |
| *Diptychandra aurantiaca Tul.* |  |  |  | Guarim Neto & Morais, 2003 |
| *Diptychandra aurantiaca subsp. epunctata (Tul.) G.P.Lewis* | DDS | Bark | Decoction With Water | Agra et al, 2007 |
| *Diptychandra aurantiaca subsp. epunctata (Tul.) G.P.Lewis* | DDS | Bark | Decoction With Water | Agra et al, 2008 |
| *Entadopsis polystachya (L.) Britton* | DSS | Root | In Natura | Coelho-Ferreira, 2009 |
| *Enterolobium contortisiliquum (Vell.) Morong* | OTHER | Bark | Decoction | Agra et al, 2007 |
| *Enterolobium contortisiliquum (Vell.) Morong* | OTHER | Bark, Fruits | Decoction | Agra et al, 2007 |
| *Enterolobium contortisiliquum (Vell.) Morong* | OTHER | Bark, Fruits | Decoction | Agra et al, 2008 |
| *Enterolobium contortisiliquum (Vell.) Morong* |  |  |  | Amorozo, 2002 |
| *Enterolobium contortisiliquum (Vell.) Morong* |  |  |  | Guarim Neto & Morais, 2003 |
| *Enterolobium contortisiliquum (Vell.) Morong* | DNS, DMC | Bark, Roots | Tea | Guarim-Neto, 2006 |
| *Enterolobium contortisiliquum (Vell.) Morong* | DDS |  |  | Lozano et al., 2014 |
| *Enterolobium ellipticum Benth.* |  |  |  | Guarim Neto & Morais, 2003 |
| *Enterolobium gummiferum (Mart.) J.F.Macbr.* | DCS | Bark | Decoction | Silva et al., 2010 |
| *Eriosema benthamianum Benth.* |  |  |  | Macedo, A. (1944) JBRJ |
| *Eriosema benthamianum Benth.* |  |  |  | Guarim Neto & Morais, 2003 |
| *Eriosema benthamianum Benth.* | OTHER | Leaves, Root | Infusion | Hirschmann & Arias, 1990 |
| *Eriosema campestre Benth.* |  |  |  | Amorozo, 2002 |
| *Eriosema campestre Benth.* |  |  |  | Amorozo, 2004 |
| *Eriosema crinitum (Kunth) G.Don* | OTHER |  |  | Rodrigues, 2007 |
| *Eriosema platycarpon Micheli* |  |  |  | Guarim Neto & Morais, 2003 |
| *Erythrina crista-galli L.* | OTHER | Bark | Infusion | Battisti et al, 2013 |
| *Erythrina dominguezii Hassl.* | DDS, IPD, ENM |  |  | Nunes et al, 2003 |
| *Erythrina falcata Benth.* |  |  |  | Botrel et al., 2006 |
| *Erythrina speciosa Andrews* |  |  |  | Fernandes et al., 2014 |
| *Erythrina velutina Willd.* |  |  |  | Susiany; Camila; Camila (2010) IPA |
| *Erythrina velutina Willd.* |  |  |  | Oliveira, O.F. de (1980) UFERSA |
| *Erythrina velutina Willd.* | DSS, DNS | Leaves | Decoction | Silva, S.I. (1985) UFPE |
| *Erythrina velutina Willd.* | DNS, DRS, DEM, IPD | Bark | Infusion | Agra et al, 2007 |
| *Erythrina velutina Willd.* | DNS, DRS, IPD | Bark | Infusion With Water | Agra et al, 2007 |
| *Erythrina velutina Willd.* | DNS, DRS, IPD | Bark | Infusion With Water | Agra et al, 2008 |
| *Erythrina velutina Willd.* |  |  |  | Albuquerque & Oliveira, 2007 |
| *Erythrina velutina Willd.* | DNS, DRS | Leaves, Stem |  | Albuquerque et al, 2007 |
| *Erythrina velutina Willd.* |  | Bark |  | Alencar et al., 2010 |
| *Erythrina velutina Willd.* | DNS, DFS, OTHER | Bark, Flower | Decoction | Almeida et al., 2006 |
| *Erythrina velutina* | other, DNS |  |  | de Albuquerque, 2006 |
| *Erythrina velutina Willd.* |  |  |  | Roque et al., 2010 |
| *Erythrina velutina Willd.* |  |  |  | Silva e Andrade, 2005 |
| *Erythrina verna Vell.* | DRS, OTHER |  |  | Londoño, P.A. (2010) UFPE |
| *Erythrina verna Vell.* |  |  |  | Fernandes et al., 2014 |
| *Erythrina verna Vell.* | DNS | Bark | Tea | Pinto et al., 2013 |
| *Erythrina verna Vell.* | DRS, DDS |  |  | Souza & Felfilli, 2006 |
| *Galactia glaucescens Kunth* |  |  |  | A.L. Minari (2006) UFMS |
| *Galactia glaucescens Kunth* |  |  |  | Guarim Neto & Morais, 2003 |
| *Galactia martii DC.* | DDS | Whole Plant | Decoction | Hirschmann & Arias, 1990 |
| *Geoffroea spinosa Jacq.* |  |  |  | Pickel, B. (1929) IPA |
| *Geoffroea spinosa Jacq.* |  |  |  | Roque et al., 2010 |
| *Gleditsia amorphoides (Griseb.) Taub.* | ENM | Pod | Syrup | Trojan-Rodrigues et al, 2012 |
| *Glycine max (L.) Merr.* | ENM, DCS | Seeds |  | Campelo & Ramalho, 1989 |
| *Hymenaea courbaril L.* | DRS | Resin |  | Cortas, A. (1991) INPA |
| *Hymenaea courbaril L.* | DBI, DCS |  |  | L.P. de Queiroz; et al. (2000) UEL |
| *Hymenaea courbaril L.* |  |  |  | S.A. Cunha (2009) UFMS |
| *Hymenaea courbaril L.* |  |  |  | R. Shardong (1997) UFMS |
| *Hymenaea courbaril L.* | DBI, DCS |  |  | Queiroz, L.P. de (2000) UFPB |
| *Hymenaea courbaril L.* |  |  |  | Rocha, E.A. (2005) UFPB |
| *Hymenaea courbaril L.* |  |  |  | Agra, M.F. (1997) UFPB |
| *Hymenaea courbaril L.* | DFS | Bark | Syrup | Suzene I. da Silva (1985) UFPE |
| *Hymenaea courbaril L.* | ENM, DRS | Bark, Resin, Fruit | Decoction With Water, Syrup | Agra et al, 2007 |
| *Hymenaea courbaril L.* | ENM, DRS | Bark, Resin, Fruit | Decoction With Water, Syrup | Agra et al, 2008 |
| *Hymenaea courbaril L.* |  |  |  | Albuquerque & Oliveira, 2007 |
| *Hymenaea courbaril L.* | DRS, ENM, DDS | Bark, Leaves |  | Albuquerque et al, 2007 |
| *Hymenaea courbaril L.* |  | Bark |  | Alencar et al., 2010 |
| *Hymenaea courbaril L.* |  |  |  | Amorozo, 2002 |
| *Hymenaea courbaril L.* |  |  |  | Amorozo, 2004 |
| *Hymenaea courbaril L.* | DRS, DDS | Bark | Syrup | Bratti et al., 2013 |
| *Hymenaea courbaril L.* | DRS |  |  | Breitbach et al., 2013 |
| *Hymenaea courbaril L.* |  |  |  | Brito & Senna-Valle, 2012 |
| *Hymenaea courbaril L.* | DDS, DRS | Bark | Infusion | Bueno et al., 2005 |
| *Hymenaea courbaril L.* | ENM, DGS, DRS, NEO | Fruit, Seeds, Bark | Drink, Bath | Cartaxo et al, 2010 |
| *Hymenaea courbaril L.* | DRS, OTHER | Bark, Flowers, Resin | Plaster | Chaves & Barros, 2012 |
| *Hymenaea courbaril L.* |  |  |  | Cunha & Bortolotto, 2011 |
| *Hymenaea courbaril L.* | DRS | Bark | Syrup | de Albuquerque, 2006 |
| *Hymenaea courbaril L.* |  |  |  | Fernandes et al., 2014 |
| *Hymenaea courbaril L.* | DRS | Bark | Syrup, Inhale, Tea | Gomes & Bandeira, 2012 |
| *Hymenaea courbaril L.* |  |  |  | Guarim Neto & Morais, 2003 |
| *Hymenaea courbaril L.* | DRS, DDS, DMC | Bark, Resin | Inhale, Tea | Guarim-Neto, 2006 |
| *Hymenaea courbaril L.* |  |  |  | Kainer & Duryea, 1992 |
| *Hymenaea courbaril L.* | ENM | Bark | Alcoholic Infusion | Monteles & Pinheiro, 2007 |
| *Hymenaea courbaril L.* |  |  |  | Morais et al, 2005 |
| *Hymenaea courbaril L.* | DRS, ENM | Bark |  | Oliveira et al., 2010 |
| *Hymenaea courbaril L.* | DRS, ENM, OTHER | Bark | Decoction | Santos et al, 2008 |
| *Hymenaea courbaril L.* |  |  |  | Silva & Albuquerque, 2005 |
| *Hymenaea courbaril L.* |  |  |  | Silva & Proença, 2008 |
| *Hymenaea courbaril L.* | DRS |  |  | Souza, 2007 |
| *Hymenaea courbaril var. altissima (Ducke) Lee & Langenh.* |  |  |  | F. A. Sobrinho (2006) JBRJ |
| *Hymenaea eriogyne Benth.* | DDS | Bark |  | Oliveira et al., 2010 |
| *Hymenaea intermedia Ducke* | ENM, DRS | Bark, Resin, Fruit | Decoction With Water, Syrup | Agra et al, 2007 |
| *Hymenaea intermedia Ducke* | ENM, DRS | Bark, Resin, Fruit | Decoction With Water, Syrup | Agra et al, 2008 |
| *Hymenaea martiana Hayne* |  |  |  | Heringer, E.P. (1974) JBRJ |
| *Hymenaea martiana Hayne* |  |  |  | S.A. Cunha (2008) UFMS |
| *Hymenaea martiana Hayne* | ENM, DRS | Bark, Resin, Fruit | Decoction With Water, Syrup | Agra et al, 2007 |
| *Hymenaea martiana Hayne* | ENM, DRS | Bark, Resin, Fruit | Decoction With Water, Syrup | Agra et al, 2008 |
| *Hymenaea martiana Hayne* | DRS, OTHER | Bark | Alcoholic Infusion Or Syrup | Chaves & Barros, 2012 |
| *Hymenaea martiana Hayne* |  |  |  | Cunha & Bortolotto, 2011 |
| *Hymenaea martiana Hayne* | OTHER | Bark, Seed |  | Gazzaneo et al., 2005 |
| *Hymenaea martiana Hayne* |  |  |  | Silva e Andrade, 2005 |
| *Hymenaea oblongifolia Huber* | ENM, DRS | Bark, Resin, Fruit | Decoction With Water, Syrup | Agra et al, 2007 |
| *Hymenaea oblongifolia Huber* | ENM, DRS | Bark, Resin, Fruit | Decoction With Water, Syrup | Agra et al, 2008 |
| *Hymenaea parvifolia Huber* | ENM, DRS | Bark, Resin, Fruit | Decoction With Water, Syrup | Agra et al, 2007 |
| *Hymenaea parvifolia Huber* | ENM, DRS | Bark, Resin, Fruit | Decoction With Water, Syrup | Agra et al, 2008 |
| *Hymenaea parvifolia Huber* | DRS | Bark | Syrup | Coelho-Ferreira, 2009 |
| *Hymenaea parvifolia Huber* |  |  |  | Shanley & Rosa, 2004 |
| *Hymenaea rubriflora Ducke* |  |  |  | Agra, M.F. (1988) UFPB |
| *Hymenaea stigonocarpa Hayne* |  |  |  | A.L.A. Arruda (2006) UFMS |
| *Hymenaea stigonocarpa Hayne* |  |  |  | C.N. da Cunha (1986) UFMS |
| *Hymenaea stigonocarpa Hayne* |  |  |  | Rocha, E.A. (2005) UFPE |
| *Hymenaea stigonocarpa Hayne* |  |  |  | Rocha, E.A. (2005) UFPE |
| *Hymenaea stigonocarpa Hayne* | ENM, DRS | Bark, Resin, Fruit | Decoction With Water, Syrup | Agra et al, 2007 |
| *Hymenaea stigonocarpa Hayne* | ENM, DRS | Bark, Resin, Fruit | Decoction With Water, Syrup | Agra et al, 2008 |
| *Hymenaea stigonocarpa Hayne* | DBI, ENM, DGS, DDS | Bark | Alcoholic Infusion | Aguiar & Barros, 2012 |
| *Hymenaea stigonocarpa Hayne* |  |  |  | Amorozo, 2002 |
| *Hymenaea stigonocarpa Hayne* | DGS, DRS, DDS | Bark | Tea | Barbosa da Silva et al. 2012 |
| *Hymenaea stigonocarpa Hayne* | DDS | Bark | Tea | Borba & Macedo, 2006 |
| *Hymenaea stigonocarpa Hayne* | IPD | Resin |  | Brandao et al, 2012 |
| *Hymenaea stigonocarpa Hayne* | DGS | Resin |  | Brandão et al, 2012 |
| *Hymenaea stigonocarpa Hayne* | DRS, OTHER | Bark | Syrup | Chaves & Barros, 2012 |
| *Hymenaea stigonocarpa Hayne* | DDS, ENM | Bark, Resin |  | Conceição et al, 2011 |
| *Hymenaea stigonocarpa Hayne* | DRS, OTHER | Bark | Alcoholic Infusion | Franco & Barros, 2006 |
| *Hymenaea stigonocarpa Hayne* |  |  |  | Guarim Neto & Morais, 2003 |
| *Hymenaea stigonocarpa Hayne* |  |  |  | Guarim Neto & Pasa, 2009 |
| *Hymenaea stigonocarpa Hayne* | DRS, DDS, DMC | Bark, Resin | Inhale, Tea | Guarim-Neto, 2006 |
| *Hymenaea stigonocarpa Hayne* | DDS, DRS | Bark Resin | Raw | Hirschmann & Arias, 1990 |
| *Hymenaea stigonocarpa Hayne* | OTHER | Bark | Syrup | Jesus et al., 2009 |
| *Hymenaea stigonocarpa Hayne* | DRS |  |  | Lozano et al., 2014 |
| *Hymenaea stigonocarpa Hayne* | DRS, ENM, DGS |  |  | Nunes et al, 2003 |
| *Hymenaea stigonocarpa Hayne* | DBI | Flower | Tea, Syrup | Pasa et al, 2011 |
| *Hymenaea stigonocarpa Hayne* | DRS | Bark, Resin | Tea, Syrup | Pinto et al., 2013 |
| *Hymenaea stigonocarpa Hayne* | OTHER |  |  | Rodrigues, 2007 |
| *Hymenaea stigonocarpa Hayne* | OTHER |  |  | Souza & Felfilli, 2006 |
| *Hymenaea stigonocarpa Hayne* | DRS |  |  | Souza, 2007 |
| *Hymenaea stigonocarpa Hayne* | DBI, DRS, OTHER | Bark, Resin, Fruit | Decoction | Vila Verde et al, 2003 |
| *Hymenolobium excelsum Ducke* |  |  |  | Guarim Neto & Morais, 2003 |
| *Indigofera hirsuta L.* |  |  |  | Cunha & Bortolotto, 2011 |
| *Indigofera suffruticosa Mill.* | OTHER | Leaves | Tea | Cortas, A. (1991) INPA |
| *Indigofera suffruticosa Mill.* |  |  |  | Vasconcelos, J.O.M. de (1974) UFERSA |
| *Indigofera suffruticosa Mill.* |  |  |  | Oliveira, O.F. de (1973) UFERSA |
| *Indigofera suffruticosa Mill.* |  |  |  | Oliveira, O.F. de (1973) UFERSA |
| *Indigofera suffruticosa Mill.* | DRS |  |  | A. Borges (1990) UFMS |
| *Indigofera suffruticosa Mill.* |  |  |  | S.A. Cunha (2009) UFMS |
| *Indigofera suffruticosa Mill.* |  |  |  | A. Oliveira (1990) UFMS |
| *Indigofera suffruticosa Mill.* | DDS, DGS | Leaves, Bark | Infusion | Agra et al, 2007 |
| *Indigofera suffruticosa Mill.* | DDS, DGS | Leaves, Roots | Infusion With Water | Agra et al, 2007 |
| *Indigofera suffruticosa Mill.* | DDS, DGS | Leaves, Roots | Infusion With Water | Agra et al, 2008 |
| *Indigofera suffruticosa Mill.* | OTHER | Leaves | Bath, Tea | Begossi et al, 1993 |
| *Indigofera suffruticosa Mill.* | DDS | Root | Tea | Coelho-Ferreira, 2009 |
| *Indigofera suffruticosa Mill.* |  |  |  | Fernandes et al., 2014 |
| *Indigofera suffruticosa Mill.* |  |  |  | Guarim Neto & Morais, 2003 |
| *Indigofera suffruticosa mill* |  |  |  | Guarim Neto & Morais, 2003 |
| *Inga acuminata Benth.* |  |  |  | Milliken, W. (1993) INPA |
| *Inga alba (Sw.) Willd.* | DDS | Bark | Decoction | Agra et al, 2007 |
| *Inga alba (Sw.) Willd.* | DDS | Bark | Decoction | Agra et al, 2008 |
| *Inga heterophylla Willd.* | DCS, DDS | Bark | Tea | Coelho-Ferreira, 2009 |
| *Inga heterophylla Willd.* |  |  |  | Guarim Neto & Morais, 2003 |
| *Inga ingoides (Rich.) Willd.* | DRS |  |  | Pickel, B. (1928) IPA |
| *Inga semialata (Vell.) C.Mart.* |  |  |  | Brandão et al, 2012 |
| *Inga thibaudiana DC.* |  |  |  | Shanley & Rosa, 2004 |
| *Inga vera Willd.* |  |  |  | S.A. Cunha (2009) UFMS |
| *Inga vera Willd.* |  |  |  | Cunha & Bortolotto, 2011 |
| *Leucaena leucocephala (Lam.) de Wit* |  |  |  | Sobrinho, J.S. (1965) IPA |
| *Leucaena leucocephala (Lam.) de Wit* |  |  |  | Silva, W.B. da (1973) UFERSA |
| *Leucaena leucocephala (Lam.) de Wit* |  |  |  | J.S. Sobrinho (1965) UFRPE |
| *Leucaena leucocephala (Lam.) de Wit* | OTHER | Oil Of Seeds |  | Agra et al, 2007 |
| *Leucaena leucocephala (Lam.) de Wit* | OTHER | Oil Of Seeds |  | Agra et al, 2008 |
| *Leucaena leucocephala (Lam.) de Wit* | ENM |  |  | Lozano et al., 2014 |
| *Libidibia ferrea (Mart. ex Tul.) L.P.Queiroz* | DDS, NEO, DRS, OTHER | Bark, Root, Leaves, Fruit | Tea | Cortas, A. (1989) INPA |
| *Libidibia ferrea (Mart. ex Tul.) L.P.Queiroz* | DRS | Bark | Tea | Cortas, A. (1991) INPA |
| *Libidibia ferrea (Mart. ex Tul.) L.P.Queiroz* |  |  |  | Rech, A.R. (2009) INPA |
| *Libidibia ferrea (Mart. ex Tul.) L.P.Queiroz* | OTHER |  |  | Borges, O.B. (1993) JBRJ |
| *Libidibia ferrea (Mart. ex Tul.) L.P.Queiroz* | OTHER | Tincture |  | Vasconcellos Sobrinho (1936) JBRJ |
| *Libidibia ferrea (Mart. ex Tul.) L.P.Queiroz* |  |  |  | Gadelha Neto, P.C. (2012) UFPB |
| *Libidibia ferrea (Mart. ex Tul.) L.P.Queiroz* |  |  |  | Gadelha Neto, P.C. (2012) UFPB |
| *Libidibia ferrea (Mart. ex Tul.) L.P.Queiroz* |  |  |  | Gadelha Neto, P.C. (2012) UFPB |
| *Libidibia ferrea (Mart. ex Tul.) L.P.Queiroz* |  |  |  | Almeida, C. (2006) UFPE |
| *Libidibia ferrea (Mart. ex Tul.) L.P.Queiroz* | DRS | Bark | Tea | Barbosa da Silva et al. 2012 |
| *Lonchocarpus costatus Benth.* |  |  |  | Brandão et al, 2012 |
| *Lonchocarpus nicou (Aubl.) DC.* | DSS, OTHER | Root | Juice, In Natura | Coelho-Ferreira, 2009 |
| *Machaerium aculeatum Raddi* |  |  |  | Amorozo, 2002 |
| *Machaerium aculeatum Raddi* |  |  |  | Amorozo, 2004 |
| *Machaerium eriocarpum Benth.* | OTHER | Bark | Tea | Jesus et al., 2009 |
| *Machaerium isadelphum (E.Mey.) Standl.* |  |  |  | F.F. Dias (2006) UFMS |
| *Machaerium isadelphum (E.Mey.) Standl.* |  |  |  | Silva e Andrade, 2005 |
| *Machaerium lunatum (L.f.) Ducke* | DFS | Root | Tea | Coelho-Ferreira, 2009 |
| *Machaerium opacum Vogel* | IPD | Leaves | Infusion With Water | Agra et al, 2008 |
| *Machaerium opacum Vogel* | IPD | Leaves | Infusion With Water | Agra et al, 2008 |
| *Machaerium stipitatum (DC.) Vogel* |  |  |  | Brandão et al, 2012 |
| *Macroptilium lathyroides (L.) Urb.* | DCS | Leaves | Decoction With Water | Agra et al, 2007 |
| *Macroptilium lathyroides (L.) Urb.* | DCS | Leaves | Decoction With Water | Agra et al, 2008 |
| *Macroptilium lathyroides (L.) Urb.* | DBI, OTHER | Bark, Flowers, | Decoction | Almeida et al., 2006 |
| *Macroptilium lathyroides (L.) Urb.* |  |  |  | Guarim Neto & Morais, 2003 |
| *Martiodendron mediterraneum (Benth.) R.C.Koeppen* |  |  |  | F. C. S. Oliveira (2007) JBRJ |
| *Martiodendron mediterraneum (Benth.) R.C.Koeppen* | OTHER |  |  | Rodrigues, 2007 |
| *Medicago sativa L.* |  |  |  | Veneziani, R.C.S.; Furtado, N.A.J.C. (2006) UEL |
| *Melanoxylon brauna Schott* | DDS, DFS |  |  | Souza & Felfilli, 2006 |
| *Mimosa acutistipula Benth.* |  |  |  | Agra, M.F.; Silva, M.G.; Silva, M.G. (1993) IPA |
| *Mimosa acutistipula Benth.* |  |  |  | Minari, A.L.; et al. (2006) UEL |
| *Mimosa acutistipula Benth.* |  |  |  | G. Teixeira (1965) UFRPE |
| *Mimosa acutistipula Benth.* | DFS |  | Tea | Setz, E.Z.F. (1979) UNICAMP |
| *Mimosa acutistipula Benth.* | DNS, DDS | Bark | Decoction | Agra et al, 2007 |
| *Mimosa acutistipula Benth.* | DNS, DDS | Bark | Decoction | Agra et al, 2008 |
| *Mimosa acutistipula Benth.* | DDS, DNS | Bark | Infusion | Chaves & Barros, 2012 |
| *Mimosa adenocarpa Benth.* | DDS | Branches | Tea | Borba & Macedo, 2006 |
| *Mimosa adenocarpa Benth.* |  |  |  | Guarim Neto & Morais, 2003 |
| *Mimosa arenosa (Willd.) Poir.* | DMC | Leaves | Decoction | Agra et al, 2007 |
| *Mimosa arenosa (Willd.) Poir.* | DMC | Leaves | Decoction | Agra et al, 2008 |
| *Mimosa arenosa (Willd.) Poir.* |  |  |  | Silva & Albuquerque, 2005 |
| *Mimosa bimucronata (DC.) Kuntze* |  |  |  | Patzlaff, R. (2000) JBRJ |
| *Mimosa bimucronata (DC.) Kuntze* |  |  |  | F. A. Sobrinho (2006) JBRJ |
| *Mimosa caesalpiniifolia Benth.* |  |  |  | Oliveira, O.F. de (1973) UFERSA |
| *Mimosa caesalpiniifolia Benth.* |  |  |  | Oliveira, O.F. de (1973) UFERSA |
| *Mimosa caesalpiniifolia Benth.* |  |  |  | Oliveira, O.F. de (1973) UFERSA |
| *Mimosa caesalpiniifolia Benth.* |  |  |  | Oliveira, O.F. de (1972) UFERSA |
| *Mimosa caesalpiniifolia Benth.* |  |  |  | Oliveira, O.F. de (1973) UFERSA |
| *Mimosa caesalpiniifolia Benth.* | DRS | Bark | Syrup | Agra et al, 2007 |
| *Mimosa caesalpiniifolia Benth.* | DRS | Bark | Syrup | Agra et al, 2008 |
| *Mimosa caesalpiniifolia Benth.* | DRS, DDS | Bark | Juice | Aguiar & Barros, 2012 |
| *Mimosa caesalpiniifolia Benth.* | DCS, OTHER | Bark, Flowers | Decoction | Almeida et al., 2006 |
| *Mimosa caesalpiniifolia Benth.* | OTHER |  |  | Cartaxo et al, 2010 |
| *Mimosa caesalpiniifolia Benth.* | DRS | Bark | Syrup | Chaves & Barros, 2012 |
| *Mimosa caesalpiniifolia Benth.* | DCS | Bark |  | Oliveira et al., 2010 |
| *Mimosa caesalpiniifolia Benth.* |  |  |  | Sousa et al., 2012 |
| *Mimosa invisa Colla* |  |  |  | Silva e Andrade, 2005 |
| *Mimosa nuda Benth.* |  |  |  | Oliveira, O.F. de (1972) UFERSA |
| *Mimosa ophthalmocentra Benth.* | DRS | Bark | Decoction | Agra et al, 2007 |
| *Mimosa ophthalmocentra Benth.* | DRS | Bark | Decoction | Agra et al, 2007 |
| *Mimosa ophthalmocentra Benth.* | DRS | Bark | Decoction | Agra et al, 2008 |
| *Mimosa ophthalmocentra Benth.* | OTHER | Bark | Syrup | Silva & Freire, 2010 |
| *Mimosa pudica L.* |  |  |  | Mendonca, S. (1976) INPA |
| *Mimosa pudica L.* |  |  |  | Sobrinho, J.S. (1965) IPA |
| *Mimosa pudica L.* |  |  |  | Oliveira, O.F. de (1972) UFERSA |
| *Mimosa pudica L.* |  | Leaves |  | Azevedo & Kruel, 2007 |
| *Mimosa pudica L.* |  |  |  | Brito & Senna-Valle, 2012 |
| *Mimosa pudica L.* |  |  |  | Figueiredo et al., 1993 |
| *Mimosa pudica L.* | other | Leaves | Plaster | Garcia et al., 2010 |
| *Mimosa pudica L.* |  |  |  | Guarim Neto & Morais, 2003 |
| *Mimosa pudica L.* | DEM |  |  | Lozano et al., 2014 |
| *Mimosa pudica var. tetrandra L.* |  |  |  | S.A. Cunha (2009) UFMS |
| *Mimosa quadrivalvis var. leptocarpa (DC.) Barneby* |  |  |  | Mendonca, S. (1976) INPA |
| *Mimosa quadrivalvis var. leptocarpa (DC.) Barneby* |  |  |  | Oliveira, O.F. de (1973) UFERSA |
| *Mimosa quadrivalvis var. leptocarpa (DC.) Barneby* | DGS | Roots | Decoction | Agra et al, 2008 |
| *Mimosa quadrivalvis var. leptocarpa (DC.) Barneby* | DGS | Roots | Decoction | Agra et al, 2008 |
| *Mimosa quadrivalvis var. leptocarpa (DC.) Barneby* |  |  |  | Silva e Andrade, 2005 |
| *Mimosa sensitiva L.* | DNS |  |  | Aline Redondo Martins (2002) UNESPSJRP |
| *Mimosa sensitiva L.* |  |  |  | Silva & Albuquerque, 2005 |
| *Mimosa setosa Benth.* |  |  |  | Carneiro, F. (1953) UFPB |
| *Mimosa setosa Benth.* |  |  |  | Cunha & Bortolotto, 2011 |
| *Mimosa tenuiflora (Willd.) Poir.* |  |  |  | Oliveira, O.F. de (1972) UFERSA |
| *Mimosa tenuiflora (Willd.) Poir.* |  |  |  | Oliveira, O.F. de (1972) UFERSA |
| *Mimosa tenuiflora (Willd.) Poir.* |  |  |  | Oliveira, O.F. de (1972) UFERSA |
| *Mimosa tenuiflora (Willd.) Poir.* |  |  |  | Moura, O.T. (1993) UFPB |
| *Mimosa tenuiflora (Willd.) Poir.* |  |  |  | G.T. Soldati (2008) UFRPE |
| *Mimosa tenuiflora (Willd.) Poir.* |  |  |  | J.S. Sobrinho (1965) UFRPE |
| *Mimosa tenuiflora (Willd.) Poir.* | DRS | Bark | Decoction | Agra et al, 2007 |
| *Mimosa tenuiflora (Willd.) Poir.* | DRS | Bark | Decoction | Agra et al, 2007 |
| *Mimosa tenuiflora (Willd.) Poir.* | DRS | Bark | Decoction | Agra et al, 2008 |
| *Mimosa tenuiflora (Willd.) Poir.* |  |  |  | Albuquerque & Oliveira, 2007 |
| *Mimosa tenuiflora (Willd.) Poir.* | DNS | Bark | Infusion | Albuquerque, 2001 |
| *Mimosa tenuiflora (Willd.) Poir.* |  | Barl |  | Alencar et al., 2010 |
| *Mimosa tenuiflora (Willd.) Poir.* | DFS, OTHER , | Bark, Flowers | Decoction | Almeida et al., 2006 |
| *Mimosa tenuiflora (Willd.) Poir.* | OTHER | Bark | Drink, Wash, Bath | Cartaxo et al, 2010 |
| *Mimosa tenuiflora (Willd.) Poir.* | OTHER | Bark | Bath | Castro et al., 2011 |
| *Mimosa tenuiflora (Willd.) Poir.* | ENM, DDS | Root | Alcoholic Infusion | Chaves & Barros, 2012 |
| *Mimosa tenuiflora (Willd.) Poir.* | OTHER | Bark | Plaster | Gomes & Bandeira, 2012 |
| *Mimosa tenuiflora (Willd.) Poir.* |  |  |  | Roque et al., 2010 |
| *Mimosa tenuiflora (Willd.) Poir.* |  |  |  | Silva & Albuquerque, 2005 |
| *Mimosa velloziana Mart.* | DCS, OTHER | Root |  | Oliveira et al., 2010 |
| *Mimosa verrucosa Benth.* | DNS | Bark | Decoction Or Syrup | Agra et al, 2007 |
| *Mimosa verrucosa Benth.* | DNS | Bark | Decoction Or Syrup | Agra et al, 2008 |
| *Mimosa verrucosa Benth.* | DFS | Bark | Tea | Aguiar & Barros, 2012 |
| *Mimosa verrucosa Benth.* | DNS, OTHER , |  |  | Desmarchelier et al., 1999 |
| *Mimosa xanthocentra var. subsericea (Benth.) Barneby* |  |  |  | A.L. Minari (2006) UFMS |
| *Mucuna pruriens (L.) DC.* |  |  |  | Lima, V.C. (1991) IPA |
| *Mucuna pruriens (L.) DC.* |  |  |  | Lima, V.C. (1991) UNICAMP |
| *Myrocarpus frondosus Allemao* |  |  |  | Than, R.M. (2001) JBRJ |
| *Myrocarpus frondosus Allemao* |  |  |  | Than, R.M. (2001) JBRJ |
| *Myrocarpus frondosus Allemao* | DRS |  |  | Ritter et al., 2002 |
| *Myroxylon balsamum (L.) Harms* |  |  |  | Kainer & Duryea, 1992 |
| *Myroxylon peruiferum L.f.* |  |  |  | U.M. Resende (2002) UFMS |
| *Myroxylon peruiferum L.f.* | DRS |  |  | Xavier, L.P. (1942) UFPB |
| *Myroxylon peruiferum L.f.* |  |  |  | Espinola, M.C. (1945) UFPB |
| *Myroxylon peruiferum L.f.* | OTHER | Bark | Wash | Cartaxo et al, 2010 |
| *Myroxylon peruiferum L.f.* |  |  |  | Roque et al., 2010 |
| *Myroxylon peruiferum L.f.* |  |  |  | Zucchi et al., 2013 |
| *Ormosia coutinhoi Ducke* | DDS, OTHER | Bark | Tea, Alcoholic Infusion | Coelho-Ferreira, 2009 |
| *Parapiptadenia rigida (Benth.) Brenan* | DRS | Bark | Infusion | Battisti et al, 2013 |
| *Parapiptadenia rigida (Benth.) Brenan* | DNS, DMC, OTHER | Bark |  | Conceição et al, 2011 |
| *Parapiptadenia rigida (Benth.) Brenan* |  |  |  | Guarim Neto & Morais, 2003 |
| *Parapiptadenia rigida (Benth.) Brenan* | DRS |  |  | Ritter et al., 2002 |
| *Parapiptadenia zehntneri (Harms) M.P.Lima & H.C.Lima* | DRS, DFS | Bark | Decoction | Almeida et al., 2006 |
| *Parapiptadenia zehntneri (Harms) M.P.Lima & H.C.Lima* |  |  |  | Silva & Albuquerque, 2005 |
| *Parkia pendula (Willd.) Walp.* |  |  |  | Matins, M.V. (1995) UFG |
| *Parkia platycephala Benth.* | OTHER |  |  | Lozano et al., 2014 |
| *Parkinsonia aculeata L.* | OTHER | Seeds | Tea | Agra et al, 2007 |
| *Parkinsonia aculeata L.* | OTHER | Seeds | Tea | Agra et al, 2007 |
| *Parkinsonia aculeata L.* | OTHER | Seeds | Tea | Agra et al, 2008 |
| *Parkinsonia aculeata L.* | ENM, DCS, DNS, OTHER | Bark, Leaves |  | Albuquerque et al, 2007 |
| *Parkinsonia aculeata L.* | DRS, ENM, DCS | Bark, Flower | Decoction | Almeida et al., 2006 |
| *Parkinsonia aculeata L.* | DDS, DCS | Seeds, Leaves |  | Oliveira et al., 2010 |
| *Peltogyne lecointei Ducke* |  |  |  | Shanley & Rosa, 2004 |
| *Peltogyne pauciflora Benth.* | DDS, DNS | Leaves | Decoction With Water | Agra et al, 2008 |
| *Peltogyne pauciflora Benth.* | DDS, DRS, DNS | Leaves |  | Albuquerque et al, 2007 |
| *Peltogyne pauciflora Benth.* | DNS, DRS | Leaves, Flower | Decoction | Almeida et al., 2006 |
| *Peltogyne pauciflora Benth.* |  |  |  | Silva & Albuquerque, 2005 |
| *Peltophorum dubium (Spreng.) Taub.* | others | Bark |  | Bueno et al., 2005 |
| *Pentaclethra macroloba (Willd.) Kuntze* |  |  |  | Pires, M.J. (1985) INPA |
| *Periandra coccinea (Schrad.) Benth.* | DFS | Leaves | Tea | Voeks & Leony, 2004 |
| *Periandra mediterranea (Vell.) Taub.* |  |  |  | Guedes, T.N. (1957) INPA |
| *Periandra mediterranea (Vell.) Taub.* |  |  |  | Guedes, T.N. (1957) IPA |
| *Periandra mediterranea (Vell.) Taub.* | DRS | Root |  | Barbosa, M.R. (2002) UFPB |
| *Periandra mediterranea (Vell.) Taub.* | DRS | Root |  | Felix, L.P. (1988) UFPB |
| *Periandra mediterranea (Vell.) Taub.* | DRS | Root |  | Felix, L.P. (1989) UFPB |
| *Periandra mediterranea (Vell.) Taub.* | DRS | Root |  | Silva, A.L. da (1900) UFPB |
| *Periandra mediterranea (Vell.) Taub.* |  |  |  | Rita Lima (2008) UFPB |
| *Periandra mediterranea (Vell.) Taub.* |  |  |  | Moura, O.T. (1991) UFPB |
| *Periandra mediterranea (Vell.) Taub.* |  |  |  | Xavier, L.P. (1951) UFPB |
| *Periandra mediterranea (Vell.) Taub.* | DRS, ENM | Roots | Decoction | Agra et al, 2007 |
| *Periandra mediterranea (Vell.) Taub.* | DRS, ENM | Roots | Decoction | Agra et al, 2008 |
| *Periandra mediterranea (Vell.) Taub.* | DRS | Root | Tea | Barbosa da Silva et al. 2012 |
| *Periandra mediterranea (Vell.) Taub.* |  |  |  | Brandão et al, 2012 |
| *Periandra mediterranea (Vell.) Taub.* | DRS | Root | Tea | Gomes & Bandeira, 2012 |
| *Periandra mediterranea (Vell.) Taub.* |  |  |  | Guarim Neto & Morais, 2003 |
| *Periandra mediterranea (Vell.) Taub.* | DRS, DGS |  |  | Souza & Felfilli, 2006 |
| *Phanera radiata (Vell.) Vaz* |  |  |  | Fernandes et al., 2014 |
| *Phanera riedeliana (Bong.)Vaz* |  |  |  | A. Pott (2002) UFMS |
| *Phanera riedeliana (Bong.)Vaz* |  |  |  | A. Pott (2002) UFMS |
| *Phanera splendens (Kunth) Vaz* | IPD |  |  | Kinupp, V.F.; Ming, L.C.; Vieira, D.A.F.; Araújo, A.A. (2009) UEL |
| *Phaseolus vulgaris L.* | IPD | Seed | Tea | Coelho-Ferreira, 2009 |
| *Phaseolus vulgaris L.* | ENM |  |  | Feijo et al., 2012 |
| *Piptadenia gonoacantha (Mart.) J.F.Macbr.* |  |  |  | Guarim Neto & Morais, 2003 |
| *Piptadenia moniliformis Benth.* | DRS | Bark | Tea | Aguiar & Barros, 2012 |
| *Piptadenia moniliformis Benth.* | DDS, IPD | Bark | Infusion Or Plaster | Chaves & Barros, 2012 |
| *Piptadenia obliqua (Pers.) J.F.Macbr.* |  |  |  | LondoÑo, P.A. (2010) UFPE |
| *Piptadenia obliqua (Pers.) J.F.Macbr.* | DDS, ENM | Bark | Decoction | Agra et al, 2008 |
| *Piptadenia obliqua (Pers.) J.F.Macbr.* |  |  |  | Silva & Albuquerque, 2005 |
| *Piptadenia stipulacea (Benth.) Ducke* |  |  |  | Barbosa, J.I.S.; Silva, A. .G.; Silva, M. .V.; Santana, T.; Silva, A. .G.; Silva, M. .V.; Santana, T. (2009) IPA |
| *Piptadenia stipulacea (Benth.) Ducke* |  |  |  | Agra, M.F. (1993) UFPB |
| *Piptadenia stipulacea (Benth.) Ducke* |  |  |  | G.T. Soldati (2008) UFRPE |
| *Piptadenia stipulacea (Benth.) Ducke* |  |  |  | G. Teixeira (1965) UFRPE |
| *Piptadenia stipulacea (Benth.) Ducke* |  |  |  | J.S. Sobrinho (1965) UFRPE |
| *Piptadenia stipulacea* | other |  |  | de Albuquerque, 2006 |
| *Piptadenia stipulacea (Benth.) Ducke* | DDS, DFS | Bark, Root | Tea, Bath | Gomes & Bandeira, 2012 |
| *Piptadenia stipulacea (Benth.) Ducke* |  |  |  | Silva & Albuquerque, 2005 |
| *Piptadenia viridiflora (Kunth) Benth.* |  |  |  | Teixeira, G. (1965) IPA |
| *Piptadenia viridiflora (Kunth) Benth.* | DDS, DRS | Bark | Decoction | Agra et al, 2007 |
| *Piptadenia viridiflora (Kunth) Benth.* | DDS, DRS | Bark | Decoction | Agra et al, 2008 |
| *Piptadenia viridiflora (Kunth) Benth.* | DCS, DMC, DGS, OTHER | Root | Tea, Maceration | Gomes & Bandeira, 2012 |
| *Piptadenia viridiflora (Kunth) Benth.* |  |  |  | Silva & Albuquerque, 2005 |
| *Pithecellobium cochliacarpum (Gomes) J.F. Macbr.* | DFS, OTHER | Bark, Latex |  | Gazzaneo et al., 2005 |
| *Pithecellobium cochliacarpum (Gomes) J.F. Macbr.* |  |  |  | Silva e Andrade, 2005 |
| *Pithecellobium diversifolium Benth.* | ENM, DRS, DFS | Bark | Decoction Or Bath | Agra et al, 2007 |
| *Pithecellobium diversifolium Benth.* | ENM, DRS, DFS | Bark | Decoction Or Bath | Agra et al, 2008 |
| *Pithecellobium diversifolium Benth.* | ENM, DRS, DFS | Bark | Decoction | Almeida et al., 2006 |
| *Pityrocarpa moniliformis (Benth.) Luckow & R. W. Jobson* |  |  |  | Rocha, E.A.; Bezerra, E.; Bezerra, E. (2005) IPA |
| *Pityrocarpa moniliformis (Benth.) Luckow & R. W. Jobson* |  |  |  | Rocha, E.A. (2005) UFPE |
| *Plathymenia reticulata Benth.* |  |  |  | U.M. Resende (2002) UFMS |
| *Plathymenia reticulata Benth.* | DDS | Bark | Gargle | Borba & Macedo, 2006 |
| *Plathymenia reticulata Benth.* |  |  |  | Guarim Neto & Morais, 2003 |
| *Plathymenia reticulata Benth.* |  |  |  | Guarim Neto & Pasa, 2009 |
| *Plathymenia reticulata Benth.* | DCS | Bark | Bath | Guarim-Neto, 2006 |
| *Plathymenia reticulata Benth.* | OTHER |  |  | Rodrigues, 2007 |
| *Plathymenia reticulata Benth.* | DCS | Bark | Infusion | Silva et al., 2010 |
| *Platycyamus regnellii Benth.* | DCS | Bark | Infusion | Silva et al., 2010 |
| *Platymiscium floribundum Vogel* |  |  |  | Brandão et al, 2012 |
| *Platypodium elegans Vogel* |  |  |  | Guarim Neto & Morais, 2003 |
| *Poeppigia procera C.Presl* |  |  |  | Silva & Albuquerque, 2005 |
| *Poincianella bracteosa (Tul.) L.P.Queiroz* |  |  |  | Almeida, C. (2006) UFPE |
| *Poincianella bracteosa (Tul.) L.P.Queiroz* |  |  |  | Almeida, C. (2006) UFPE |
| *Poincianella pyramidalis (Tul.) L.P.Queiroz* | DDS | Leaves, Bark | Tea | Lima, H.C. de (1990) JBRJ |
| *Poincianella pyramidalis (Tul.) L.P.Queiroz* |  |  |  | ROBERTO PAULO ORLANDI (1988) JBRJ |
| *Poincianella pyramidalis (Tul.) L.P.Queiroz* |  |  |  | P. CAMPOS PORTI (1920) JBRJ |
| *Poincianella pyramidalis (Tul.) L.P.Queiroz* | DDS | Leaves, Roots, Bark, Flowers | Tea, Maceration | Gomes & Bandeira, 2012 |
| *Poiretia latifolia Vogel* | DDS, OTHER | Leaves, Root | Tea | s.c. (1991) UEM |
| *Prosopis juliflora (Sw.) DC.* |  |  |  | Almeida, C. (2006) UFPE |
| *Prosopis juliflora (Sw.) DC.* | DDS, DEA | Leaves | Decoction | Agra et al, 2007 |
| *Prosopis juliflora (Sw.) DC.* | DDS, DEA | Leaves | Decoction | Agra et al, 2008 |
| *Pterocarpus villosus (Benth.) Benth.* | DNS, ENM | Flowers | Infusion | Chaves & Barros, 2012 |
| *Pterocarpus villosus (Benth.) Benth.* | OTHER | Bark |  | Oliveira et al., 2010 |
| *Pterocarpus violaceus Vogel* | DRS, OTHER | Bark | Decoction | Agra et al, 2007 |
| *Pterocarpus violaceus Vogel* | DRS, OTHER | Bark | Decoction | Agra et al, 2008 |
| *Pterodon emarginatus Vogel* | IPD |  |  | Melo, L.R.V. (2008) INPA |
| *Pterodon emarginatus Vogel* |  |  |  | R. C. Mendonca (1998) JBRJ |
| *Pterodon emarginatus Vogel* | DRS | Seed |  | Sartori, A.L.B.; Pott, A.; Lima, L.C.P.; Moraes, M.D.; Aragaki, S.; Pott, V.J. (2001) UEL |
| *Pterodon emarginatus Vogel* |  |  |  | Carvalho, M.G.; Silva, S.T. (1991) UEL |
| *Pterodon emarginatus Vogel* |  |  |  | Borges, R.W.W.; et al. (1988) UEL |
| *Pterodon emarginatus Vogel* |  |  |  | S.A. Cunha (2009) UFMS |
| *Pterodon emarginatus Vogel* | DMC | Bark, Seeds | Decoction | Agra et al, 2007 |
| *Pterodon emarginatus Vogel* | DMC | Bark, Seeds | Decoction | Agra et al, 2008 |
| *Pterodon emarginatus Vogel* | ENM, DRS | Seed | Maceration | Borba & Macedo, 2006 |
| *Pterodon emarginatus Vogel* |  |  |  | Brandão et al, 2012 |
| *Pterodon emarginatus Vogel* | others |  |  | Bueno et al., 2005 |
| *Pterodon emarginatus Vogel* | ENM, DNS, DRS | Leaves, Flowers, Root |  | Conceição et al, 2011 |
| *Pterodon emarginatus Vogel* | DMC |  |  | Conde et al., 2014 |
| *Pterodon emarginatus Vogel* |  |  |  | Cunha & Bortolotto, 2011 |
| *Pterodon emarginatus Vogel* |  |  |  | Guarim Neto & Morais, 2003 |
| *Pterodon emarginatus Vogel* |  |  |  | Guarim Neto & Pasa, 2009 |
| *Pterodon emarginatus Vogel* | ENM | Leaves, Oil | Tea | Macedo & Ferreira, 2005 |
| *Pterodon emarginatus Vogel* | IPD | Bark, Seeds | Tea, Bath | Macedo et al, 2011 |
| *Pterodon emarginatus Vogel* | DEM, DRS, ENM, DDS |  |  | Nunes et al, 2003 |
| *Pterodon emarginatus Vogel* | DRS | Fruit | Syrup | Pasa et al, 2011 |
| *Pterodon emarginatus Vogel* | DRS, DMC | Seed, Leaves | Tea, Syrup | Pinto et al., 2013 |
| *Pterodon emarginatus Vogel* | DRS |  |  | Souza & Felfilli, 2006 |
| *Pterodon emarginatus Vogel* | IPD, DRS | Seed | Tincture | Vila Verde et al, 2003 |
| *Rhynchosia phaseoloides (Sw.) DC.* |  |  |  | Botrel et al., 2006 |
| *Schizolobium parahyba (Vell.) S.F.Blake* |  |  |  | Miranda et al., 2011 |
| *Sclerolobium aureum (Tul.) Baill.* |  |  |  | Guarim Neto & Morais, 2003 |
| *Sclerolobium aureum (Tul.) Baill.* | OTHER |  |  | Rodrigues, 2007 |
| *Sclerolobium paniculatum Vogel* | OTHER | Bark |  | Conceição et al, 2011 |
| *Sclerolobium paniculatum Vogel* | OTHER | Bark | Topic Use | Franco & Barros, 2006 |
| *Senegalia martiusiana (Steud.) Seigler & Ebinger* |  |  |  | Fernandes et al., 2014 |
| *Senegalia polyphylla (DC.) Britton* |  |  |  | Veloso, T.M.G. (2000) UFPB |
| *Senegalia polyphylla (DC.) Britton* |  |  |  | Espinola, M.C. (1941) UFPB |
| *Senegalia polyphylla (DC.) Britton* |  |  |  | J.S. Sobrinho (1965) UFRPE |
| *Senna aculeata (Benth.) H.S.Irwin & Barneby* | DRS, DFS, ENM |  |  | LondoÑo, P.A. (2010) UFPE |
| *Senna aculeata (Benth.) H.S.Irwin & Barneby* | IPD | Leaves | Tea, Bath | Macedo et al, 2011 |
| *Senna alata (L.) Roxb.* |  |  |  | Oliveira, F.C.S (2007) JBRJ |
| *Senna alata (L.) Roxb.* |  |  |  | Katia Ferreira (2007) JBRJ |
| *Senna alata (L.) Roxb.* |  |  |  | T.M.R. dos Santos (2009) UFMS |
| *Senna alata (L.) Roxb.* | DCS |  |  | Silva, S.I. (1985) UFPE |
| *Senna alata (L.) Roxb.* | DSS | Leaves | Decoction With Water | Agra et al, 2007 |
| *Senna alata (L.) Roxb.* | DSS | Leaves | Decoction With Water | Agra et al, 2008 |
| *Senna alata (L.) Roxb.* | DSS | Bark | Alcoholic Infusion | Aguiar & Barros, 2012 |
| *Senna alata (L.) Roxb.* | DCS, ENM | Leaves |  | Albuquerque et al, 2007 |
| *Senna alata (L.) Roxb.* | ENM | Flowers | Infusion | Albuquerque, 2001 |
| *Senna alata (L.) Roxb.* |  |  |  | Amorozo, 2002 |
| *Senna alata (L.) Roxb.* | DRS | Root | Tea, Syrup | Castro et al., 2011 |
| *Senna alata (L.) Roxb.* |  |  |  | Cunha & Bortolotto, 2011 |
| *Senna alata (L.) Roxb.* | DRS | Flowers | Tea | Franco & Barros, 2006 |
| *Senna alata (L.) Roxb.* |  |  |  | Guarim Neto & Morais, 2003 |
| *Senna alata (L.) Roxb.* | DFS | Leaves | Tea | Oliveira & Menini Neto, 2012 |
| *Senna alata (L.) Roxb.* | OTHER |  |  | Rodrigues, 2007 |
| *Senna alata (L.) Roxb.* |  |  |  | Silva e Andrade, 2005 |
| *Senna alexandrina Mill.* |  |  |  | Oliveira, F.C.S (2007) JBRJ |
| *Senna alexandrina Mill.* | DDS | Leaves | Infusion With Water | Agra et al, 2008 |
| *Senna alexandrina Mill.* | DDS, ENM | Leaves |  | Albuquerque et al, 2007 |
| *Senna alexandrina Mill.* | ENM, DRS | Leaves | Drink | Cartaxo et al, 2010 |
| *Senna alexandrina Mill.* | ENM | Leaves |  | Dickel et al, 2007 |
| *Senna alexandrina Mill.* | DDS |  |  | Giraldi & Hanazaki, 2010 |
| *Senna alexandrina Mill.* | OTHER | Leaves | Decoction | Liporacci & Simao, 2013 |
| *Senna alexandrina Mill.* | DDS | Leaves | Tea | Pinto et al., 2013 |
| *Senna auriculata (L.) Roxb.* | ENM | Flower | Alcoholic Extraction | Dornas et al., 2008 |
| *Senna cana (Nees & Mart.) H.S.Irwin* |  |  |  | Andrade-Lima (1956) IPA |
| *Senna cana (Nees & Mart.) H.S.Irwin* |  |  |  | Silva & Albuquerque, 2005 |
| *Senna cernua (Balb.) H.S.Irwin & Barneby* | DMC, OTHER |  |  | Londoño, P.A. (2010) UFPE |
| *Senna chrysocarpa (Desv.) H.S.Irwin & Barneby* |  |  |  | Ferreira, K. (2006) JBRJ |
| *Senna corifolia (Benth.) H.S.Irwin & Barneby* |  |  |  | Barata, L.E.S. (2007) UNICAMP |
| *Senna corymbosa (Lam.) H.S.Irwin & Barneby* |  |  |  | A.C.S. Brito (2008) UFMS |
| *Senna corymbosa (Lam.) H.S.Irwin & Barneby* | DFS, DBI, other | Seed | Tincture | Oliveira et al., 2010 |
| *Senna cumingii var. alcaparra (Phil.) H.S.Irwin & Barneby* |  |  |  | Sobrinho, J.S. (1965) IPA |
| *Senna georgica H.S.Irwin & Barneby* |  |  |  | A.C.S. Brito (2009) UFMS |
| *Senna georgica H.S.Irwin & Barneby* | DDS | Leaves | Infusion With Water | Agra et al, 2008 |
| *Senna georgica H.S.Irwin & Barneby* |  |  |  | Silva e Andrade, 2005 |
| *Senna hirsuta (L.) H.S.Irwin & Barneby* |  |  |  | Guarim Neto & Morais, 2003 |
| *Senna macranthera (Collad.) H.S.Irwin & Barneby* |  |  |  | Yara (1989) JBRJ |
| *Senna macranthera (Collad.) H.S.Irwin & Barneby* |  |  |  | Machado, E.A. (1947) JBRJ |
| *Senna macranthera (Collad.) H.S.Irwin & Barneby* |  |  |  | Xavier, L.P. (1942) UFPB |
| *Senna martiana (Benth.) H.S.Irwin & Barneby* | OTHER | Root |  | Mattos Silva, L.A. (1988) JBRJ |
| *Senna martiana (Benth.) H.S.Irwin & Barneby* | DDS | Leaves | Infusion | Agra et al, 2007 |
| *Senna martiana (Benth.) H.S.Irwin & Barneby* | DDS, DFS | Leaves | Infusion With Water | Agra et al, 2007 |
| *Senna martiana (Benth.) H.S.Irwin & Barneby* | DDS, DFS | Leaves | Infusion With Water | Agra et al, 2008 |
| *Senna martiana (Benth.) H.S.Irwin & Barneby* | DRS, DDS, IPD | Leaves, Flowers |  | Albuquerque et al, 2007 |
| *Senna martiana (Benth.) H.S.Irwin & Barneby* | DRS | Bark | Syrup | de Albuquerque, 2006 |
| *Senna martiana (Benth.) H.S.Irwin & Barneby* |  |  |  | Guarim Neto & Morais, 2003 |
| *Senna multijuga (Rich.) H.S.Irwin & Barneby* | DDS | Leaves |  | Breitbach et al., 2013 |
| *Senna oblongifolia (Vogel) H.S.Irwin & Barneby* |  |  |  | Oliveira, F. de (1977) JBRJ |
| *Senna obtusifolia (L.) H.S.Irwin & Barneby* | OTHER | Root |  | Nelson, B.W. (1980) INPA |
| *Senna obtusifolia (L.) H.S.Irwin & Barneby* |  |  |  | Cassino, M.F. (2010) INPA |
| *Senna obtusifolia (L.) H.S. Irwin & Barneby* |  |  |  | Cardoso, A. (1996) IPA |
| *Senna obtusifolia (L.) H.S.Irwin & Barneby* |  |  |  | Leitao, F. (2005) JBRJ |
| *Senna obtusifolia (L.) H.S.Irwin & Barneby* |  |  |  | Miranda, A.M.; et al. (2002) UEL |
| *Senna obtusifolia (L.) H.S.Irwin & Barneby* |  |  |  | Regalado, L.G. (1973) UFERSA |
| *Senna obtusifolia (L.) H.S.Irwin & Barneby* |  |  |  | Chagas, F.N.; Garcia, J.P.; Alves, N.R. (1976) UNICAMP |
| *Senna obtusifolia (L.) H.S.Irwin & Barneby* | DDS, DFS, DNS, DMC | Leaves | Infusion With Water | Agra et al, 2008 |
| *Senna obtusifolia (L.) H.S.Irwin & Barneby* | DDS, DFS, DNS, DMC | Leaves | Infusion With Water | Agra et al, 2008 |
| *Senna obtusifolia (L.) H.S.Irwin & Barneby* | DRS | Leaves | Syrup | Aguiar & Barros, 2012 |
| *Senna obtusifolia (L.) H.S.Irwin & Barneby* | DDS | Leaves, Root |  | Albuquerque et al, 2007 |
| *Senna obtusifolia (L.) H.S.Irwin & Barneby* | DDS | Leaves, Root | Decoction | Almeida et al., 2006 |
| *Senna obtusifolia (L.) H.S.Irwin & Barneby* |  |  |  | Fernandes et al., 2014 |
| *Senna obtusifolia (L.) H.S.Irwin & Barneby* | DRS, DDS | Seed, Root, Flower | Tea | Franco & Barros, 2006 |
| *Senna obtusifolia (L.) H.S.Irwin & Barneby* | DRS | Root, Leaves |  | Gazzaneo et al., 2005 |
| *Senna obtusifolia (L.) H.S.Irwin & Barneby* |  |  |  | Guarim Neto & Morais, 2003 |
| *Senna obtusifolia (L.) H.S.Irwin & Barneby* |  |  |  | Roque et al., 2010 |
| *Senna occidentalis (L.) Link* | DNS |  |  | Oliveira, E.L.P.G. de (1980) INPA |
| *Senna occidentalis (L.) Link* | OTHER | Root | Tea | Cortas, A. (1991) INPA |
| *Senna occidentalis (L.) Link* |  |  |  | Souza, L.A.G. de (2009) INPA |
| *Senna occidentalis (L.) Link* |  |  |  | S.Col. INPA |
| *Senna occidentalis (L.) Link* |  |  |  | Staviski, M.N.R.; Lyra, R.P.; Lyra, R.P. (1982) IPA |
| *Senna occidentalis (L.) Link* |  |  |  | Staviski, M.N.R. (1982) IPA |
| *Senna occidentalis (L.) Link* |  |  |  | Quimelato, M. (2005) JBRJ |
| *Senna occidentalis (L.) Link* | OTHER |  |  | L. A. Santos Silva (1988) MBML |
| *Senna occidentalis (L.) Link* |  |  |  | Paiva, S.A. (1973) UFERSA |
| *Senna occidentalis (L.) Link* |  |  |  | Holanda, J.S. de (1973) UFERSA |
| *Senna occidentalis (L.) Link* | DRS | Leaves |  | M.C.C. Chagas (2002) UFMS |
| *Senna occidentalis (L.) Link* |  |  |  | S.A. Cunha (2008) UFMS |
| *Senna occidentalis (L.) Link* |  |  |  | F.F. Dias (2006) UFMS |
| *Senna occidentalis (L.) Link* |  |  |  | A. Oliveira (1993) UFMS |
| *Senna occidentalis (L.) Link* |  |  |  | A. Borges (1990) UFMS |
| *Senna occidentalis (L.) Link* |  |  |  | R. Shardong (1999) UFMS |
| *Senna occidentalis (L.) Link* |  |  |  | V.J. de Moura (2002) UFMS |
| *Senna occidentalis (L.) Link* |  |  |  | M.C.C. Chagas (2002) UFMS |
| *Senna occidentalis (L.) Link* |  |  |  | Staviski, M.N.R. (1983) UFPB |
| *Senna occidentalis (L.) Link* |  |  |  | Sousa, M.A. (1976) UFPB |
| *Senna occidentalis (L.) Link* |  |  |  | Agra, M.F. (1900) UFPB |
| *Senna occidentalis (L.) Link* | DFS, DBI |  |  | Campelo, M.J.A. (1996) UFPE |
| *Senna occidentalis (L.) Link* | DFS, DBI |  |  | Campelo, M.J.A. (1996) UFPE |
| *Senna occidentalis (L.) Link* | DSS, DFS, OTHER | Root, Seeds | Decoction | Silva, S.I. (1985) UFPE |
| *Senna occidentalis (L.) Link* |  |  |  | Oliveira, G.L. de (2005) UFPE |
| *Senna occidentalis (L.) Link* |  |  |  | Siqueira-Filho, J.A. (1996) UFPE |
| *Senna occidentalis (L.) Link* | DNS | Leaves | Infusion | Agra et al, 2007 |
| *Senna occidentalis (L.) Link* | DNS | Leaves, Seed | Decoction With Water | Agra et al, 2007 |
| *Senna occidentalis (L.) Link* | DNS | Leaves, Seed | Decoction With Water | Agra et al, 2008 |
| *Senna occidentalis (L.) Link* | DRS | Leaves | Syrup | Aguiar & Barros, 2012 |
| *Senna occidentalis (L.) Link* | DRS, NEO, ENM, OTHER, DFS | Leaves, Root, Seed |  | Albuquerque et al, 2007 |
| *Senna occidentalis (L.) Link* | ENM | Fruit | Infusion | Albuquerque, 2001 |
| *Senna occidentalis (L.) Link* |  | Leaves |  | Alencar et al., 2010 |
| *Senna occidentalis (L.) Link* | DRS, NEO, DDS | Leaves, Root | Decoction | Almeida et al., 2006 |
| *Senna occidentalis (L.) Link* |  |  |  | Amorozo, 2002 |
| *Senna occidentalis (L.) Link* |  |  |  | Amorozo, 2004 |
| *Senna occidentalis (L.) Link* | DRS, DDS, DEM, PCP | Leaves, Root, Flowers | Tea | Barbosa da Silva et al. 2012 |
| *Senna occidentalis (L.) Link* | DDS, OTHER | Flower, Seed, Root | Tea | Borba & Macedo, 2006 |
| *Senna occidentalis (L.) Link* | DDS, DGS | Root, Leaves |  | Brandao et al, 2008 |
| *Senna occidentalis (L.) Link* | DDS, DGS, DSS | Leaves | Tea | Bratti et al., 2013 |
| *Senna occidentalis (L.) Link* | DDS |  |  | Breitbach et al., 2013 |
| *Senna occidentalis (L.) Link* | DRS, DSS | Leaves, Flowers | Infusion Or Plaster | Chaves & Barros, 2012 |
| *Senna occidentalis (L.) Link* | DFS, DCS, NEO, DRS, ENM | Seed, Root | Tea, Alcoholic Infusion | Coelho-Ferreira, 2009 |
| *Senna occidentalis (L.) Link* |  |  |  | Cunha & Bortolotto, 2011 |
| *Senna occidentalis (L.) Link* | DMC | Root | Maceration | David & Pasa, 2013 |
| *Senna occidentalis (L.) Link* | DRS | Root | Tea, Syrup | Feijo et al., 2013 |
| *Senna occidentalis (L.) Link* | DRS, IPD, DSS | Flower, Leaves | Tea | Franco & Barros, 2006 |
| *Senna occidentalis (L.) Link* | DRS | Bark, Seed |  | Gazzaneo et al., 2005 |
| *Senna occidentalis (L.) Link* | DDS | Fruit, Leaves | Tea, Bath | Gomes & Bandeira, 2012 |
| *Senna occidentalis (L.) Link* |  |  |  | Guarim Neto & Morais, 2003 |
| *Senna occidentalis (L.) Link* | DDS, DGS | Roots | Tea | Guarim-Neto, 2006 |
| *Senna occidentalis (L.) Link* | OTHER | Root | Tea | Jesus et al., 2009 |
| *Senna occidentalis (L.) Link* |  |  |  | Kainer & Duryea, 1992 |
| *Senna occidentalis (L.) Link* | PCP |  |  | Lozano et al., 2014 |
| *Senna occidentalis (L.) Link* | DMC | Leaves |  | Oliveira & Trovāo, 2009 |
| *Senna occidentalis (L.) Link* | DRS | Seeds |  | Oliveira et al., 2010 |
| *Senna occidentalis (L.) Link* | IPD, DNS |  | Tea | Oliveira et al., 2011 |
| *Senna occidentalis (L.) Link* |  |  |  | Pasa et al, 2005 |
| *Senna occidentalis (L.) Link* | DDS | Leaves | Tea | Pasa et al, 2011 |
| *Senna occidentalis (L.) Link* |  |  |  | Pinto et al, 2006 |
| *Senna occidentalis (L.) Link* | OTHER |  |  | Rodrigues, 2007 |
| *Senna occidentalis (L.) Link* |  |  |  | Roque et al., 2010 |
| *Senna occidentalis (L.) Link* | DBI | Bark | Maceration | Silva & Freire, 2010 |
| *Senna occidentalis (L.) Link* |  |  |  | Silva & Proença, 2008 |
| *Senna occidentalis (L.) Link* |  |  |  | Silva e Andrade, 2005 |
| *Senna occidentalis (L.) Link* | DCS | Leaves | Infusion | Silva et al., 2010 |
| *Senna occidentalis (L.) Link* | DGS, DDS, DRS | Root, Leaves | Infusion | Siviero et al., 2012 |
| *Senna occidentalis (L.) Link* | DGS, DDS, DSS |  |  | Souza & Felfilli, 2006 |
| *Senna occidentalis (L.) Link* | DDS, IPD |  |  | Souza, 2007 |
| *Senna paradictyon (Vogel) H.S. Irwin & Barneby* |  |  |  | Hanazaki, N.; Barbosa, Z. (1999) UNICAMP |
| *Senna pendula (Willd.) H.S.Irwin & Barneby* |  |  |  | S.A. Cunha (2008) UFMS |
| *Senna pendula (Willd.) H.S.Irwin & Barneby* | DMC | Roots | Alcoholic Infusion | Garcia et al., 2010 |
| *Senna pilifera (Vogel) H.S.Irwin & Barneby* |  |  |  | Sousa, M.A. (1976) UFPB |
| *Senna quinquangulata (Rich.) H.S. Irwin & Barneby* |  |  |  | Lescano, L.E.A.M.; Nunes, G.P.; Noguchi, D.K. (2005) UEL |
| *Senna reticulata (Willd.) H.S.Irwin & Barneby* |  |  |  | E.P. Guedes (1966) UFRPE |
| *Senna reticulata (Willd.) H.S.Irwin & Barneby* | DSS | Leaves | Juice | Coelho-Ferreira, 2009 |
| *Senna reticulata (Willd.) H.S.Irwin & Barneby* | DSS, DDS | Flowers, Leaves |  | Oliveira et al., 2010 |
| *Senna rizzinii H.S.Irwin & Barneby* |  |  |  | Silva & Albuquerque, 2005 |
| *Senna rugosa (G.Don) H.S.Irwin & Barneby* |  |  |  | Cassino, M.F. (2010) INPA |
| *Senna rugosa (G.Don) H.S.Irwin & Barneby* |  |  |  | Guarim Neto & Morais, 2003 |
| *Senna rugosa (G.Don) H.S.Irwin & Barneby* | IPD, DRS | Leaves, Root | Decoction | Vila Verde et al, 2003 |
| *Senna spectabilis (DC.) H.S.Irwin & Barneby* | DRS, DDS, ENM | Leaves, Bark, Root, Seeds | Decoction | Agra et al, 2007 |
| *Senna spectabilis (DC.) H.S.Irwin & Barneby* | DRS, DDS, ENM | Leaves, Root, Seed, Bark | Decoction With Water | Agra et al, 2007 |
| *Senna spectabilis (DC.) H.S.Irwin & Barneby* | DRS, DDS, ENM | Leaves, Root, Seed, Bark | Decoction With Water | Agra et al, 2008 |
| *Senna spectabilis (DC.) H.S.Irwin & Barneby* | DRS, DDS | Bark, Leaves |  | Albuquerque et al, 2007 |
| *Senna spectabilis (DC.) H.S.Irwin & Barneby* | OTHER | Bark | Wash With Decoction | Cartaxo et al, 2010 |
| *Senna spectabilis (DC.) H.S.Irwin & Barneby* | DRS | Bark | Decoction | Chaves & Barros, 2012 |
| *Senna spectabilis (DC.) H.S.Irwin & Barneby* | DRS | Root | Tea | Freitas et al, 2012 |
| *Senna spectabilis (DC.) H.S.Irwin & Barneby* | DSS | Leaves |  | Oliveira et al., 2010 |
| *Senna splendida (Vogel) H.S.Irwin & Barneby* | DGS | Leaves | Decoction With Water | Agra et al, 2008 |
| *Senna splendida (Vogel) H.S.Irwin & Barneby* | DRS, DDS, DGS, DCS | Stem, Bark, Leaves, Root |  | Albuquerque et al, 2007 |
| *Senna splendida (Vogel) H.S.Irwin & Barneby* | DGS, DMC, DDS, DCS, OTHER | Bark, Stem, Leaves, Flower, Root | Decoction | Almeida et al., 2006 |
| *Senna splendida (Vogel) H.S.Irwin & Barneby* |  | Bark |  | Brandao et al, 2012 |
| *Senna splendida (Vogel) H.S.Irwin & Barneby* | ENM | Bark | Decoction | Brandão et al, 2012 |
| *Senna tora (L.) Roxb.* | DDS, DNS | Leaves, Seed | Infusion With Water | Agra et al, 2008 |
| *Senna trachypus (Benth.) H.S.Irwin & Barneby* | DDS | Bark | Decoction With Water | Agra et al, 2008 |
| *Senna trachypus (Benth.) H.S.Irwin & Barneby* | ENM, DRS, OTHER | Leaves, Flowers | Infusion | Chaves & Barros, 2012 |
| *Senna tropica (Vell.) H.S.Irwin & Barneby* | IPD, OTHER |  |  | Giraldi & Hanazaki, 2010 |
| *Senna uniflora (Mill.) H.S.Irwin & Barneby* |  |  |  | Bastos, B.C. (1981) INPA |
| *Senna uniflora (Mill.) H.S.Irwin & Barneby* |  |  |  | Costa, E. de O. (1973) UFERSA |
| *Senna uniflora (Mill.) H.S.Irwin & Barneby* |  |  |  | Oliveira, O.F. de (1972) UFERSA |
| *Senna uniflora (Mill.) H.S.Irwin & Barneby* |  |  |  | Oliveira, O.F. de (1972) UFERSA |
| *Senna uniflora (Mill.) H.S.Irwin & Barneby* |  |  |  | Oliveira, O.F. de (1972) UFERSA |
| *Senna uniflora (Mill.) H.S.Irwin & Barneby* |  |  |  | Oliveira, O.F. de (1972) UFERSA |
| *Senna uniflora (Mill.) H.S.Irwin & Barneby* |  |  |  | Oliveira, O.F. de (1972) UFERSA |
| *Senna uniflora (Mill.) H.S.Irwin & Barneby* | DDS | Leaves, Seed | Infusion With Water | Agra et al, 2007 |
| *Senna uniflora (Mill.) H.S.Irwin & Barneby* | DDS | Leaves, Seed | Infusion With Water | Agra et al, 2008 |
| *Senna uniflora (Mill.) H.S.Irwin & Barneby* |  |  |  | Guarim Neto & Morais, 2003 |
| *Senna uniflora (Mill.) H.S.Irwin & Barneby* | DCS | Root | Tea | Monteles & Pinheiro, 2007 |
| *Senna velutina (Vogel) H.S.Irwin & Barneby* | DSS | Bark | Decoction With Water | Agra et al, 2007 |
| *Senna velutina (Vogel) H.S.Irwin & Barneby* | DSS | Bark | Decoction With Water | Agra et al, 2008 |
| *Sophora tomentosa L.* |  |  |  | Brandão et al, 2012 |
| *Stryphnodendron adstringens (Mart.) Coville* | OTHER | Bark | Bath | Teles, A.M. (2003) INPA |
| *Stryphnodendron adstringens (Mart.) Coville* |  |  |  | Silva, A.P. da (1993) INPA |
| *Stryphnodendron adstringens (Mart.) Coville* |  |  |  | Cid Ferreira, C.A. (1985) JBRJ |
| *Stryphnodendron adstringens (Mart.) Coville* |  |  |  | Matins, M.V. (1992) UFG |
| *Stryphnodendron adstringens (Mart.) Coville* | OTHER |  |  | A. Abadio (2002) UFMS |
| *Stryphnodendron adstringens (Mart.) Coville* |  |  |  | A. Oliveira (1990) UFMS |
| *Stryphnodendron adstringens (Mart.) Coville* |  |  |  | A. Oliveira (1990) UFMS |
| *Stryphnodendron adstringens (Mart.) Coville* |  |  |  | C.A. Conceicao (1985) UFMS |
| *Stryphnodendron adstringens (Mart.) Coville* |  |  |  | R. Conti (2001) UFMS |
| *Stryphnodendron adstringens (Mart.) Coville* |  |  |  | J.S. Sobrinho (1965) UFRPE |
| *Stryphnodendron adstringens (Mart.) Coville* | DDS, ENM | Bark | Decoction | Agra et al, 2008 |
| *Stryphnodendron adstringens (Mart.) Coville* | DDS, DGS, OTHER | Bark | Tea | Aguiar & Barros, 2012 |
| *Stryphnodendron adstringens (Mart.) Coville* | DCS, OTHER | Bark |  | Albuquerque et al, 2007 |
| *Stryphnodendron adstringens (Mart.) Coville* |  |  |  | Amorozo, 2002 |
| *Stryphnodendron adstringens (Mart.) Coville* |  |  |  | Amorozo, 2004 |
| *Stryphnodendron adstringens (Mart.) Coville* |  |  |  | Botrel et al., 2006 |
| *Stryphnodendron adstringens (Mart.) Coville* | DDS, DFS, OTHER | Rinds |  | Brandao et al, 2012 |
| *Stryphnodendron adstringens (Mart.) Coville* | OTHER | Bark |  | Brandao et al, 2012 |
| *Stryphnodendron adstringens (Mart.) Coville* |  |  |  | Brandão et al, 2012 |
| *Stryphnodendron adstringens (Mart.) Coville* | DFS | Bark | Juice | Castro et al., 2011 |
| *Stryphnodendron adstringens (Mart.) Coville* | ENM | Leaves | Tea | David & Pasa, 2013 |
| *Stryphnodendron adstringens (Mart.) Coville* |  |  |  | Guarim Neto & Morais, 2003 |
| *Stryphnodendron adstringens (Mart.) Coville* |  |  |  | Guarim Neto & Pasa, 2009 |
| *Stryphnodendron adstringens (Mart.) Coville* | DFS, OTHER | Bark | Bath | Guarim-Neto, 2006 |
| *Stryphnodendron adstringens (Mart.) Coville* | DDS, DBI, DFS | Bark | Infusion | Hirschmann & Arias, 1990 |
| *Stryphnodendron adstringens (Mart.) Coville* | OTHER |  |  | Nunes et al, 2003 |
| *Stryphnodendron adstringens (Mart.) Coville* | OTHER | Bark | Bath | Oliveira & Menini Neto, 2012 |
| *Stryphnodendron adstringens* | DDS |  |  | Pasa et al, 2011 |
| *Stryphnodendron adstringens (Mart.) Coville* | OTHER | Bark | Soap, Bath | Pinto et al., 2013 |
| *Stryphnodendron adstringens (Mart.) Coville* | DFS, OTHER | Bark |  | Shanley and Luz, 2003 |
| *Stryphnodendron adstringens (Mart.) Coville* | OTHER |  |  | Souza & Felfilli, 2006 |
| *Stryphnodendron adstringens (Mart.) Coville* | DFS, OTHER | Bark | Bath, Tea, Alcoholic Infusion | Souza, 2007 |
| *Stryphnodendron adstringens (Mart.) Coville* | DDS, OTHER | Bark | Decoction | Vila Verde et al, 2003 |
| *Stryphnodendron coriaceum Benth.* | DDS, DFS, ENM | Bark |  | Conceição et al, 2011 |
| *Stryphnodendron obovatum Benth.* |  |  |  | R. Schardong (1999) UFMS |
| *Stryphnodendron obovatum Benth.* |  |  |  | A.C.S. Brito (2008) UFMS |
| *Stryphnodendron obovatum Benth.* |  |  |  | Cunha & Bortolotto, 2011 |
| *Stryphnodendron obovatum Benth.* |  |  |  | Guarim Neto & Morais, 2003 |
| *Stryphnodendron obovatum Benth.* | OTHER | Bark | Decoction | Jesus et al., 2009 |
| *Stryphnodendron obovatum Benth.* | DFS, OTHER, DDS, DSS | Bark |  | Ustulin et al., 2008 |
| *Stryphnodendron polyphyllum Mart.* |  |  |  | Fernandes et al., 2014 |
| *Stryphnodendron rotundifolium Mart.* | OTHER |  | Tea | R.C. Mendonca (1997) JBRJ |
| *Stryphnodendron rotundifolium Mart.* | DRS | Bark | Decoction | Matins, M.V. (1995) UFG |
| *Stryphnodendron rotundifolium Mart.* |  |  |  | S.A. Cunha (2008) UFMS |
| *Stryphnodendron rotundifolium Mart.* | OTHER |  |  | Lozano et al., 2014 |
| *Stylosanthes viscosa Sw.* |  |  |  | Guarim Neto & Morais, 2003 |
| *Styphnolobium japonicum (L.) Schott* | ENM | Bark | Alcoholic Extraction | Dornas et al., 2008 |
| *Swartzia flaemingii Raddi* | DDS, DMC | Leaves, Bark | Decoction | Agra et al, 2007 |
| *Swartzia flaemingii Raddi* | DDS, DMC | Leaves, Bark | Decoction | Agra et al, 2008 |
| *Swartzia flaemingii Raddi* | DMC, DDS, ENM | Bark, Leaves | Alcoholic Infusion | Chaves & Barros, 2012 |
| *Swartzia flaemingii Raddi* | DMC, DGS, DFS, ENM, DCS | Bark, Leaves |  | Oliveira et al., 2010 |
| *Tamarindus indica L.* |  |  |  | Oliveira, O.F. de (1974) UFERSA |
| *Tamarindus indica L.* |  |  |  | Nogueira, J.R. (1973) UFERSA |
| *Tamarindus indica L.* |  |  |  | Lima, J.E. da C. (1973) UFERSA |
| *Tamarindus indica L.* |  |  |  | Silva Neto, J.G. da (1973) UFERSA |
| *Tamarindus indica L.* |  |  |  | S.A. Cunha (2008) UFMS |
| *Tamarindus indica L.* |  |  |  | Gadelha Neto, P.C. (1995) UFPB |
| *Tamarindus indica L.* | DRS, DGS, DDS | Leaves, Fruit | Decoction With Water | Agra et al, 2008 |
| *Tamarindus indica L.* | IPD | Fruit | In Natura | Aguiar & Barros, 2012 |
| *Tamarindus indica L.* |  |  |  | Amorozo, 2002 |
| *Tamarindus indica L.* |  |  |  | Amorozo, 2004 |
| *Tamarindus indica L.* | DRS | Fruit | Drink | Cartaxo et al, 2010 |
| *Tamarindus indica L.* |  |  |  | Cunha & Bortolotto, 2011 |
| *Tamarindus indica L.* | ENM | Seed | Aquous Extraction | Dornas et al., 2008 |
| *Tamarindus indica L.* | IPD | Leaves | Tea | Franco & Barros, 2006 |
| *Tamarindus indica L.* | DGS | Bark |  | Gazzaneo et al., 2005 |
| *Tamarindus indica L.* | OTHER | Leaves | Tinctures | Guarim-Neto, 2006 |
| *Tamarindus indica L.* | DDS | Leaves, Flowers |  | Oliveira et al., 2010 |
| *Tamarindus indica L.* | IPD | Leaves | Tea | Pasa et al, 2011 |
| *Tamarindus indica L.* | DRS | Leaves | Bath | Pinto et al., 2013 |
| *Tamarindus indica L.* |  |  |  | Silva & Proença, 2008 |
| *Tamarindus indica L.* |  |  |  | Silva e Andrade, 2005 |
| *Taralea oppositifolia Aubl.* | DDS, DNS, DCS | Seeds |  | Breitbach et al., 2013 |
| *Tephrosia adunca Benth.* |  |  |  | Guarim Neto & Morais, 2003 |
| *Tephrosia cinerea (L.) Pers.* | OTHER | Whole Plant | Decoction | Agra et al, 2008 |
| *Tephrosia purpurea (L.) Pers.* |  |  |  | Grise Veloso, T.M. (2001) IPA |
| *Tephrosia purpurea (L.) Pers.* |  |  |  | Veloso, T.M.G. (2001) UFPB |
| *Tephrosia purpurea (L.) Pers.* |  | Leaves |  | Alencar et al., 2010 |
| *Tephrosia purpurea (L.) Pers.* | DRS | Leaves | Tea | Barbosa da Silva et al. 2012 |
| *Tipuana tipu (Benth.) Kuntze* | OTHER | Leaves | Infusion | Löbler et al, 2014 |
| *Trigonella foenum-graecum L.* | ENM | Seeds, Leaves | Aquous Extraction | Dornas et al., 2008 |
| *Vatairea guianensis Aubl.* | DSS |  |  | Prance, G.T. (1975) INPA |
| *Vatairea guianensis Aubl.* | DSS |  |  | Lima, H.C. de (1987) INPA |
| *Vatairea guianensis Aubl.* | DSS | Fruit | In Natura | Coelho-Ferreira, 2009 |
| *Vatairea macrocarpa (Benth.) Ducke* |  |  |  | S.A. Cunha (2009) UFMS |
| *Vatairea macrocarpa (Benth.) Ducke* |  |  |  | I.M. Bortolotto (2004) UFMS |
| *Vatairea macrocarpa (Benth.) Ducke* |  |  |  | Amorozo, 2002 |
| *Vatairea macrocarpa (Benth.) Ducke* |  |  |  | Cunha & Bortolotto, 2011 |
| *Vatairea macrocarpa (Benth.) Ducke* |  |  |  | Guarim Neto & Morais, 2003 |
| *Vatairea macrocarpa (Benth.) Ducke* | OTHER | Bark | Decoction | Jesus et al., 2009 |
| *Vatairea macrocarpa (Benth.) Ducke* | NEO | Flowers, Root |  | Oliveira et al., 2010 |
| *Vatairea macrocarpa (Benth.) Ducke* | DDS |  | Tea | Oliveira et al., 2011 |
| *Vatairea macrocarpa (Benth.) Ducke* | DDS | Bark | Maceration | Souza, 2007 |
| *Vicia faba L.* | DSS |  |  | Lozano et al., 2014 |
| *Vigna unguiculata (L.) Walp.* | DSS |  |  | Lozano et al., 2014 |
| *Vouacapoua americana Aubl.* | DCS | Wood | Tea | Coelho-Ferreira, 2009 |
| *Zollernia ilicifolia (Brongn.) Vogel* | DCS, DDS, DGS, OTHER |  |  | Giraldi & Hanazaki, 2010 |
| *Zornia brasiliensis Vogel* | DDS, DGS | Bark | Infusion | Agra, M.F. (1986) UFPB |
| *Zornia brasiliensis Vogel* |  |  |  | Agra, M.F. (1999) UFPB |
| *Zornia brasiliensis Vogel* | DGS | Whole Plant | Decoction | Agra et al, 2007 |
| *Zornia brasiliensis Vogel* | DGS, DFS | Whole Plant | Decoction | Agra et al, 2007 |
| *Zornia brasiliensis Vogel* | DGS, DFS | Whole Plant | Decoction | Agra et al, 2008 |
| *Zornia brasiliensis Vogel* |  |  |  | David et al., 2007 |
| *Zornia diphylla (L.) Pers.* |  |  |  | Luetzelburg (1936) IPA |
| *Zornia diphylla (L.) Pers.* |  |  | Brazil | Felix, L.P. (1990) UFPB |
| *Zornia diphylla (L.) Pers.* | DGS, DFS | Whole Plant | Decoction | Agra et al, 2007 |
| *Zornia diphylla (L.) Pers.* | DGS, DFS | Whole Plant | Decoction | Agra et al, 2008 |
| *Zornia diphylla (L.) Pers.* | DFS, OTHER | Whole Plant |  | Gazzaneo et al., 2005 |
| *Zornia diphylla (L.) Pers.* |  |  |  | Silva & Albuquerque, 2005 |
| *Zornia glabra Desv.* | DGS, DFS | Whole Plant | Decoction | Agra et al, 2007 |
| *Zornia glabra Desv.* | DGS, DFS | Whole Plant | Decoction | Agra et al, 2008 |
| *Zornia latifolia Sm.* |  |  |  | Pinto et al, 2006 |

^1^ Citation of publication or collector date of collection and herbarium.
